# Chemotaxonomic profiling of fungal endophytes of *Solanum mauritianum* (alien weed) using gas chromatography high resolution time-of-flight mass spectrometry (GC-HRTOF-MS)

**DOI:** 10.1007/s11306-021-01790-7

**Published:** 2021-04-20

**Authors:** Sharon Pauline Pelo, Oluwafemi Ayodeji Adebo, Ezekiel Green

**Affiliations:** Department of Biotechnology and Food-Technology, Faculty of Science, University of Johannesburg, P. O. Box 17011, Doornfontein, Johannesburg, 2028 South Africa

**Keywords:** *Solanum mauritianum* Scop., Fungal endophytes, Antimicrobial, Antioxidants, Volatile organic compounds (VOC), Gas chromatography (GC)

## Abstract

**Introduction:**

Since ancient times medicinal plants have been used as medicine in many parts of the world to promote human health and longevity. In recent years many novel secondary metabolites of plants have been isolated and reported to provide lead compounds for new drug discoveries. *Solanum mauritianum* Scopoli is native to South America. It is reported to be used by native South Americans during famine as a vegetable and as medicine to cure various diseases. In South Africa the plant is viewed as weed and is facing eradication, however, this plant is a valuable subject for research into its potential pharmaceutical and chemical uses. This study elucidated the metabolic profile of fungal endophytes that have promising bioactive secondary metabolites against pathogenic microorganisms, including mycobacterium species.

**Material and methods:**

Fungal endophytes from a weed *Solanum mauritianum* Scop. were used to synthesize secondary metabolites. Gas chromatograph high-resolution time-of-flight mass spectrometry (GC-HRTOF-MS) was used to analyse volatile compounds to prove that potentially fungal endophytes could be extracted from this weed. Extracts obtained with ethyl acetate were screened for phytochemicals and analyzed using a gas chromatograph high-resolution time-of-flight mass spectrometry system. Principal component analysis was used to compare the gas chromatograph high-resolution time-of-flight mass spectrometry data for differences/similarities in their clustering. Phytochemical screening was conducted on the crude extracts of fungal endophytes obtained from different parts of *Solanum mauritianum* Scopoli (leaves, ripe fruit, unripe fruit and stems).

**Results:**

Phytochemical screening indicated the presents of alkaloids, flavonoids, glycosides, phenols, quinones and saponins. Quinones were not present in the crude extracts of *Fusarium* sp. A total of 991 compounds were observed in the fungal endophytes, and *Cladosporium* sp. (23.8%) had the highest number of compounds, compared to *Paracamarosporium leucadendri* (1.7%) and *Talaromyces* sp*.* (1.5%). Some volatile compounds such as eicosane, 2-pentadecanone, 2-methyloctacosane, hexacosane and tridecanoic acid methyl ester with antibacterial activity were also observed.

**Conclusion:**

Compositional variations between the plant and fungal endophyte phytochemicals were observed. The results of this study indicate that fungal endophytes from *Solanum mauritianum* Scop. contain compounds that can be exploited for numerous pharmaceutical and medicinal applications.

**Supplementary Information:**

The online version contains supplementary material available at 10.1007/s11306-021-01790-7.

## Introduction

Plants yield a wide range of novel secondary metabolites that are used in medicine, are a rich source of bioactive molecules. It is likely that some of the bioactive molecules from higher plants can be produced by specific endophytes (Bhagat et al., 2012). The ripe fruit and leaves of the *Solanum mauritianum* Scop. were used by native South Americans during famine as a vegetable and as medicine to cure various diseases (Jayakumar & Murugan, 2016). It is also known to be used medicinally by traditional healers in South Africa to treat diarrhoea, dysentery, infertility and menorrhagia (Uche-Okereafor et al., 2019) and colorectal cancer in Kenya (Ochwang’i, 2014). The plant is viewed as a weed in South Africa and is facing extermination (Cowie et al., 2018). A study conducted by Jayakumar and Murugan lends credence to the view of *S. mauritianum* Scop. as a medicinal plant and provides its ethnopharmacological use (Jayakumar & Murugan, 2016). *S. mauritianum* Scop. is reported to have antioxidant activities and to exhibit protective effects against H_2_O_2_-induced oxidative damage (Jayakumar & Murugan, 2016), demonstrating the potential of this plant.

Plants in their natural habitats maintain mutualistic associations with endophytic microbes, where both partners benefit from each other (Sun and Guo, 2012; Pansanit & Pripdeevech, 2018). Notably, endophytes provide protection against pathogens, herbivores and parasites, increase the plant’s tolerance to drought and low soil fertility and enhance plant growth (Shankar et al., 2014). They also help the plant’s resistance to biotic and abiotic stresses (Zhong et al., 2011). Plants harbour distinct microbes that may produce the same metabolites as the plant, which are even more notable secondary metabolites than those of their host (Luo et al., 2016). Endophytes therefore play a significant role in the micro-ecosystem of plants and are recognized as rich sources of bioactive secondary metabolites, with antimicrobial, anti-viral, anti-tumour, insecticidal, antioxidant activities and antifungal and anticancer properties (Bhatia et al., 2016; Bogner et al., 2017; Mendoza and Silva, 2018; Kaddes et al., 2019; Wu et al., 2016). These secondary metabolites can also be applied in various fields such as agriculture and the food and textile industries, and have played a significant role in drug discovery and medicine for decades (Bhatia et al., 2016; Bogner et al., 2017; Dias et al., 2012; Kaddes et al., 2019; Mendoza and Silva, 2018; Wu et al., 2016; Zhong et al., 2011).

Fungal endophytes are known to produce a particularly wide spectrum of secondary metabolites with a variety of useful biological, chemical and physical properties (Kaddes et al., 2019; Shankar et al., 2014; Zhong et al., 2011). They can be applied as bio-fumigators to replace pesticides (Kaddes et al., 2019), in industrial chemicals (benzene), in solvents (acetone, toluene, xylene), in medicine as anti-inflammatory and immunosuppressant drugs (Vasundhara et al., 2019) and in biological controls such as antifungal agents (Tilocca et al., 2020). Secondary metabolites from endophytic fungi, including alkaloids, phenols, flavonoids, hydrocarbons, quinines, volatile organic compounds (VOCs) and terpenes, all have documented anti-microbial, anti-fungal, anti-leishmanial, anti-neoplastic, anti-proliferative, antioxidant and insecticidal activities, and are well known to confer protection to the host plant (Bogner et al., 2017; Pelo et al., 2020; Sánchez-Fernández et al., 2016; Shankar et al., 2014; Vasundhara et al., 2019).

VOCs are low molecular weight compounds with high vapour pressures that cause them to evaporate at ambient temperature (Kaddes et al., 2019; Naik, 2019). The majority of VOCs belong to five chemical groups, namely, amino acids derivatives, benzenoid aromatic compounds, fatty acid derivatives, phenylpropanoids and terpenoids. Although their biosynthesis is dependent on the metabolism of primary metabolites (Kaddes et al., 2019), several studies have reported their presence in fungal endophytes (Kaddes et al., 2019; Naik, 2019; Wonglom et al., 2020). Modern instruments such as GC/MS have improved our ability to characterize fungal endophyte VOCs (Inamdar et al., 2014; Lee et al., 2016; Tilocca et al., 2020). Most, if not all, fungal species produce VOCs as mixtures of acids, alcohols, ethers, hydrocarbons, aldehydes, esters, ketones and sulphur compounds (Hung et al., 2015). This study profiled secondary metabolites produced by eight fungal endophytes isolated from *S. mauritianum.* To the best of our knowledge, this is the first documented study of fungal endophytes isolated from *Solanum mauritianum* Scopoli and the characterization of the secondary metabolites from these isolates.

## Materials and methods

### Sample preparation

*Solanum mauritianum* Scopoli plant material was collected at the University of Johannesburg Doornfontein Campus, in Johannesburg, Gauteng in South Africa (S26.11 32.6 E28.03 28.9.). The plant is part of the vegetation growing on campus. The plant material was collected in summer and winter. Fresh fruit (ripe and unripe), leaves and stems were collected from healthy plants of *S. mauritianum* Scop. and identified by the University of Johannesburg Herbarium. A voucher specimen number BTNPSP02 was issued. Fungal endophytes were isolated within 24 h of collection in the Molecular Pathogenesis and Molecular Epidemiology Research Group (MPMERG) laboratory of the Biotechnology and Food Technology Department at the University of Johannesburg and identified as reported in a previous study (Pelo et al., 2020). The fungal endophytes used in this study were initially isolated and identified in our previous study (Pelo et al., 2020). The eight fungal endophytes investigated in this study were *Paracamarosporium leucadendri, Aureobasidium pullulans, Collectotrichum boninense, Cladosporium* sp., *Fusarium* sp., *Talaromyces* sp*., Hyalodendriella* sp., and *Penicillium chrysogenum* (from the leaves), *Penicillium chrysogenum* (from the unripe fruit) (Pelo et al., 2020).

### Extraction of crude extracts from fungal endophytes

Endophytes were isolated, purified and identified, as previously reported (Huang et al., 2007; Larran et al., 2007; White et al., 1990). Molecular identification of the isolates was performed by Inqaba Biotec Africa’s Genomics Company according to Katoch et al. (2017). The identified fungal endophytes grown on potato dextrose agar (PDA) (Marcellano et al., 2017) were cut into 10 mm plugs as explained by Pelo et al., (2020) and grown on potato dextrose broth (PDB) for 14 days at 25 ℃ ± 2 ℃ under static conditions. Next, two different solvents, ethyl acetate and chloroform, were used for extraction. 100 ml of either ethyl acetate or chloroform were added to bottles, agitated and filtered. The filtrates were then left at room temperature for 24 h. The solvent phase was concentrated with a Rotavapor Labtech EV311H (Kempton Park, SA) under vacuum at 40 ℃. The crude extracts were transferred into McCartney bottles and left under laminar flow to dry.

### Phytochemical analysis

A small portion of the dry crude extract was subjected to phytochemical assays using the methods of Trease and Evans (1983), Adegboye et al. (2008), Chakraborthy et al. (2015) and Alaje et al. (2014) to test for alkaloids, cardiac glycosides, flavonoids, glycosides, phenols, quinones and saponins.

### Alkaloids

About 5 ml of 1% hydrochloric acid (HCL) solution was added to 0.5 g of the crushed plant material and placed in a boiling water bath, then 1 ml was collected into a clean container and a few drops of Dragendroff’s reagent was added. A positive reaction was indicated by turbidity or precipitation.

### Cardiac glycosides

About 2 ml of the crude extract was mixed with 1 ml of glacial acetic acid, then 2 drops of iron (III) chloride (FeCl_3_) was added, which was followed by 1 ml of concentrated sulfuric acid (H_2_SO_4_). A positive reaction was indicated by a brown ring and a ring with a violet colour, which sometimes appears below the brown coloured ring.

### Flavonoids

Approximately 0.5 g of the powdered plant sample was mixed with 10 ml of ethyl acetate and heated over a steaming water bath for 3 min and then filtered. 4 ml was removed, and 1 ml of ammonia solution was added and then shaken. Positive results were determined by a yellow colour that disappeared after a while.

### Glycosides

Approximately 1 ml of the extract was mixed with 1 ml of water in a test tube, and 3 drops of sodium hydroxide (NaOH) were added. A yellow colour indicated the presence of glycosides.

### Phenols

About 1 ml of the extract was mixed with 1 ml of water in a test tube, and 1 to 2 drops of FeCl_3_ was added. The positive presence of phenols was represented by a blue, green, red or purple colour.

### Quinones

About 1 ml of the extract was mixed with 5 ml of HCL. The formation of a yellow-coloured precipitate indicated the presence of quinone.

### Saponins

About 10 ml of all the extracts in different solvents were shaken dynamically to obtain a stable froth. The froth was then mixed with 3 drops of olive oil and shaken vigorously. Positive results were indicated by a stable formation of emulsion and froth after adding the oil.

### GC-HRTOF-MS analysis

About 1 mg of the dry crude extract (secondary metabolites) was weighed and dissolved into 1 ml of analytical grade methanol and thoroughly vortexed, and then filtered with a 0.2 µm filter syringe. Next, the samples were transferred to an autosampler vial and immediately analysed. A gas chromatography high resolution time-of-flight (GC-HRTOF-MS) instrument (LECO Corporation, St. Joseph, MI, USA) was used for the analysis, calibrated before use. Subsequent samples were analyzed using a Pegasus GC-HRTOF-MS instrument (LECO Corporation, St. Joseph, MI, USA), equipped with Agilent 7890A gas chromatography (Agilent technology, Inc, Wilmigton, DE, USA), operating in high resolution with a Gerstel MPS multipurpose auto-sampler (Gerstel Inc, Germany). The column used was a 30 m × 0.25 mm ID × 0.25 µl Rxi^®^-5 ms column (Pennslvania, US). Carrier gas used was helium at a flow rate of 1 ml/min. Samples were injected in a splitless mode, and the injection volume was 1 µl for each sample. The inlet and transfer line temperatures were set at 250 °C and 225 °C, respectively. The oven temperature was set initially to 70 °C, and maintained at this temperature for 0.5 min. It was then increased to 150 °C, at 10 °C per min, held for 2 min, and then raised to 330 °C at 10 °C per min for 3 min. The detector voltage was set at 70 eV for electronic ionization. The recommended MS data acquisition rate of 13 spectra/s *m/z* was used, with a range of 30–700.

The collected data from the GC-HRTOF-MS was formatted and processed on the LECO Chroma TOF-HRT software. Peaks and mass spectra were compared with NIST, Mainlib and Feihn metabolomics libraries. Each identified metabolite was assigned a name when the similarity value (SV) was >70%. Multivariate data analysis based on PCA was done on soft independent modelling of class analogy (SIMCA) software, version 14.1 (Umetrics, Umea, Sweden).

## Results and discussion

### Phytochemical analysis of crude extracts from fungal endophytes

Ethyl acetate and chloroform were the solvents used to extract the alkaloids, as seen in Table 1. Alkaloids were detected in all eight fungal endophyte crude extracts but were most abundant in *Cladosporium* sp., *P. chrysogenum* (leaves) and *Talaromyces* sp. extracts of both chloroform and ethyl acetate. According to Palazón et al., (2006), alkaloids are frequently obtained from bioactive compounds. One of the main characteristics of alkaloids is its ring structure that contains a nitrogen group. It is usually present in plants as salts and is easily extracted with mild acids or water and recovered with a base. Alkaloids are known to be poisonous, and so they play a significant role in ethnomedicine. Formerly, the main sources of alkaloids were the genera *Brugmansia, Datura* and *Solanaceae* (Palazón, et al., 2006). Recently, they were reported in the fungal endophytes isolated from *S. mauritianum,* including caulophyllumine-A with antioxidant activity (Jayakumar, 2016).

**Table 1:** List of phytochemicals that were screened from fungal endophytes of *S. mauritianum*

Phytochemicals	Extraction solvent	*Aureobasidium pullulans*	*Paracamarosporium leucadendri*	*Cladosporium* sp.	*Fusarium* sp.	*Hyalodendriella* sp.	*Penicillium chrysogenum* (F)	*Penicillium chrysogenum* (L)	*Talaromyces* sp.
		Fungal endophytes extracts							
Alkaloids	Ethyl acetate	+ + +	+ + +	+	+ +	+ + +	+ +	+ + + + +	+ + + + +
	Chloroform	+ +	+ +	+ + + + +	+	+	+	+ +	+ + + + +
Cardiac glycosides	Ethyl acetate	–	+ + +	+ +	+ + + + +	+	+	+ +	+ + + + +
	Chloroform	+ +	+ + +	+ + + + +	+ + + + +	+ + + + +	+	+ +	+ + +
Flavonoids	Ethyl acetate	+ + + + +	–	–	+ + + +	+ + + + +	+ + +	+ + +	+ + + + +
	Chloroform	+ + + + +	+ + +	+ + + + +	+ + + + +	+ + + + +	+ + +	+ + +	+ + + + +
Glycosides	Ethyl acetate	–	–	–	+ +	+	–	–	+ + + + +
	Chloroform	+ + + + +	–	+ + +	+ + + +	+ + +	–	+ +	+ + + +
Phenols	Ethyl acetate	+	+ +	+ +	+ +	+ +	+ +	+ +	+ +
	Chloroform	+	+ +	+ +	+ + + + +	+ +	+ +	+ +	+ + + +
Quinones	Ethyl acetate	+ + + + +	–	–	–	+ + + + +	+ + + + +	+ + + + +	+ + + + +
	Chloroform	+ + + + +	–	+ + + + +	–	+ + + + +	+ + + + +	+ + + + +	+ + + + +
Saponins	Ethyl acetate	+ + + + +	+ + + + +	+ + + + +	+ + + + +	+ + + + +	+ + + + +	+ + + + +	+ + + + +
	Chloroform	+ + + + +	+ + + + +	+ + + + +	+ + + + +	+ + + + +	+ + + + +	+ + + + +	+ + + + +

(–) Not present, ( +) least, (+ +) less, (+ + +) moderate, (+ +  + +) more, (+ +  + +) most, (F)- fruits and (L)-leaves

Cardiac glycosides were detected in all eight fungal extracts except in the ethyl acetate extracts of *A. pullulans*. They were abundant in the chloroform extracts of *Cladosporium* sp., *Fusarium* sp., *Hyalodendriella* sp., and in the ethyl acetate extracts of *Talaromyces* sp. Cardiac glycosides are known to increase the capacity of the heart to pump blood, and some, such as digoxin and digitoxin, derived from fungal endophytes of *Digitalis lanata*, are used to treat congestive heart failure, although they are toxic in high doses (Kaul et al., 2013).

Flavonoids are widely distributed as polyphenolics, and are a significant class of secondary plant metabolites, with a broad range of health benefits, including antibacterial, antifungal and antioxidant effects, associated with mitigating several diseases such as atherosclerosis, Alzheimer’s disease and cancer (Bu et al., 2016; Panche et al., 2016). They have anti-carcinogenic, anti-mutagenic, anti-inflammatory and antioxidant properties, with the ability to modulate and inhibit enzymes. They are found in different plants and indeed in different plant parts such as bark, flower, roots and stem, grains, fruits and vegetables, as well as in different beverages such as tea and wine (Shiono et al., 2011). They are also components of various cosmetic, nutraceutical, medicinal and pharmaceutical applications. In this study flavonoids were present in both crude extracts (ethyl acetate and chloroform) of the fungal endophytes *A. pullulans, Fusarium* sp., *Hyalodendriella* sp., *P. chrysogenum* (Fruit)*, P chrysogenum* (leaves) and *Talaromyces* sp*.* They were not present in the ethyl acetate extracts of *Paracamarosporium leucadendri* and *Cladosporium* sp. Overall, the presence of flavonoids in most of the endophytic extracts suggests that it may have potential benefits for human health.

Glycosides were present in more of the chloroform extracts than in the ethyl acetate extracts, and were observed in the crude extracts of *A. pullulans*, *Cladosporium* sp., *Fusarium* sp., *Hyalodendriella* sp., and *Talaromyces* sp., but were not detected in the crude extracts of *P. leucadendri* and *P. chrysogenum* (F). A similar observation was observed with *Paraconiothyrium* sp. MY-42, a fungal endophyte, and in that study the glycosides reportedly displayed anticancer properties against leukaemia cells (Shiono et al., 2011).

Phenols were present in all the crude extracts of the eight fungal endophytes, but were most abundant in the chloroform extracts of *Fusarium* sp. and *Talaromyces* sp. Phenols are known to be the best antioxidants due to their ability to scavenge free radicals in human blood plasma by transferring electrons or hydrogen atoms (Tempesta, 2004). In a study conducted on *Fritillaria unibracteata* var. *wabuensis* fungal endophytes, *Fusarium* spp. were the most abundant endophytes isolated, and produced various bioactive compounds and antioxidant phenolic compounds (Pan et al., 2017).

Quinones were not observed in either the chloroform or the ethyl acetate crude extracts of *Paracamarosporium leucadendri*, *Cladosporium* sp. and *Fusarium* sp. (Table 1). There are numerous reports on quinones regarding their biological activities and some antitumor activities, for example, emodin produced by *Aspergillus* and other fungal species shows antiparasitic activity, and some dyes and pigments contain quinine derivatives. Some of these pigments are cytotoxic, most of which originate from *Ascomycota* (Kaul et al., 2013; Yadav et al., 2019). In this study quinones were present in some of the fungal endophyte extracts, namely, *Aureobasidium pullulans*, *Cladosporium* sp., *Hyalodendriella* sp., *Penicillium chrysogenum* (F), *Penicillium chrysogenum* (L) and *Talaromyces* sp. as seen in Table 1.

Saponins were detected in all samples, which indicates the possibility of using these fungal endophytes in the production of saponin-related compounds, particularly because they are currently used as dietary supplements (Emran et al., 2015; Shan et al., 2012). Saponins are also applied in various pharmaceutical products owing to their pharmacological properties such as antiviral, anti-proliferation, anti-angiogenesis, anti-cancer, anti-inflammatory, anti-metastasis and antimicrobial activity (Wu et al., 2012). They also have cardiovascular protective activity, show reversal of multidrug resistance effects and reduce radiotherapy and chemotherapy side effects (Xu et al., 2016). *S. mauritianum* Scop fungal endophytes therefore merit attention and could be explored as a potential source for drugs in the pharmaceutical industry.

### GC-HRTOF-MS analysis of volatile compounds synthesized from fungal endophytes

**Fig. 1:**
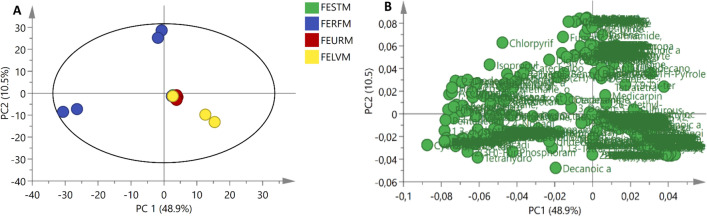
**a** PCA score plot of metabolites of fungal endophytes extracts from different plant parts and **b** PCA loadings of metabolites of fungal endophytes extracts from different plant parts. FESTM—crude extracts of the fungal endophytes isolated from the stem; FERFM—crude extracts of the fungal endophytes isolated from the ripe fruit; FEURM—crude extracts of the fungal endophytes isolated from the unripe fruits and FELVM—crude extracts of the fungal endophytes isolated from the leaves

Metabolites of the crude extracts were analysed with a GC-HRTOF-MS, and data generated was pre-processed on ChromaTOF and subsequently analysed with PCA. The resulting PCA plot (Fig. 1a) shows that the metabolites from the fungal endophytes were grouped, and they were then analysed based on the plant part from which they were isolated. This was done to understand and compare the metabolites based on which plant parts are mostly used to treat various ailments. *A. pullulans* was isolated from the stem*, P. leucadendri*, *Cladosporium* sp. and *Fusarium* sp. were isolated from the ripe fruit, *Hyalodendriella* sp. and *P. chrysogenum* (F) were isolated from the unripe fruit and *P. chrysogenum* (L) and *Talaromyces* sp. were isolated from the leaves.

The PCA score plot explained 59.4% of the data, with PC1 contributing 48.9% and PC2 contributing 10.5%. In the PCA score plot (Fig. 1a) in the PC1 direction, a separation of metabolites of eight fungal endophytes from four different plant parts (FELVM-leaves, FERFM-ripe fruit, FESTM-stem and FEURM-unripe fruit) was observed. The clear separation and clusters suggest the differences and possible similarities in metabolites based on different characteristics. As observed in Fig. 1a, the groupings and clustering of the metabolites could be attributed to the similarities in the investigated metabolites.

The metabolites of the fungal endophytes isolated from the ripe fruit (FERFM) are dispersed and are far from the metabolites from other plant parts (Fig. 1a). This suggests that fungal endophytes isolated from the ripe fruit synthesised different metabolites to those of the other metabolites of fungal endophytes from other plant parts (leaves, unripe fruit and stem). The crude extracts of fungal endophytes isolated from the ripe fruit showed inhibition against pathogenic microorganisms identified in a previous study by the authors (Pelo et al., 2020). We also observed that the metabolites from the fungal endophytes of the leaves (FELVM) are somewhat similar to those of the unripe fruit (FERFM) and stem (FESTM). A much closer clustering was observed for FESTM (*A. pullulans*) and FEURM (*Hyalodendriella* sp. and *P. chrysogenum* (F)). Most studies conducted on medicinal plants focus on the use of leaves and stem, and generally only mention fruit. A study conducted by Cowie et al. concluded that *S. mauritianum* Scop unripe fruit are poisonous as they contain the glycoalkaloid solanine (Cowie et al., 2018). Jayakumar and Murugan (Jayakumar et al., 2016) equated the consumption of ripe fruit to the use of vegetables. Studying the medicinal value of metabolites from the leaves, stem and unripe fruit of *S. mauritianum* Scop. as well as the ripe fruit constitutes a novel approach. The generated PCA loading plot depicted in Fig. 1b shows the identification and selection of metabolites, further illuminating the observed differences and similarities of grouping.

### Comparison of volatile metabolites harvested in Winter and Summer

**Fig. 2:**
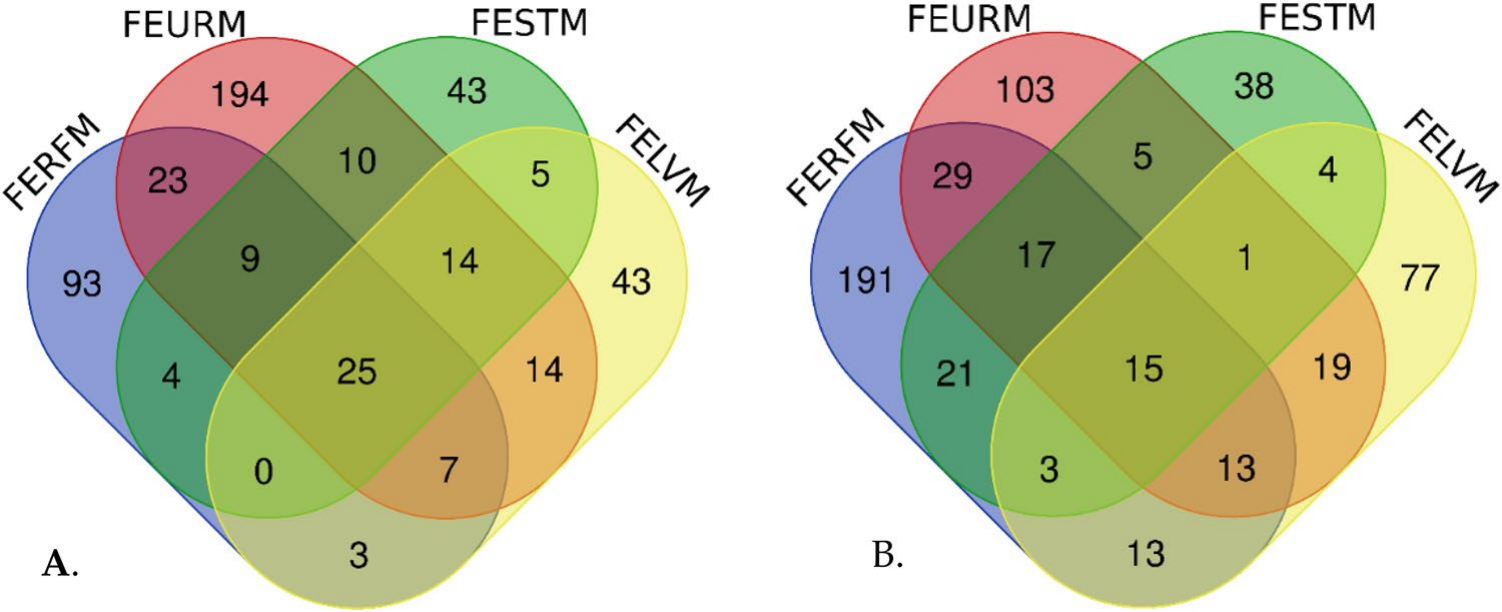
Venn diagram shows the comparison between the metabolite of fungal endophytes extracted in Winter (**a**) and Summer (**b**). The metabolites were classified based on which plant part the endophytes were isolated, FERFM—Ripe fruit fungal endophytes; FESTM—Stem fungal endophytes; FEURM—Unripe fruit fungal endophytes and FELVM—Leaves fungal endophytes

The Venn diagram in Fig. 2 shows several unique and common statistically significant metabolites of fungal endophytes, grouped according to the plant source from which they were isolated, and in which season the most metabolites were produced. The centre, where all points meet, indicates the same metabolites detected from all the 8 fungal endophyte groups according to which plant part the fungal endophytes were isolated. 25 (Fig. 2a) and 15 (Fig. 2b) metabolites were detected during winter and summer, respectively, an indication that the endophytes produced similar secondary metabolites but varied according to the season or temperature (Soni et al., 2015). Some of the similar volatile compounds analysed in winter were amines, alkenes, esters, ketones and alcohols such as 3-Eicosene, (E)-, 7-Hexadecene, (Z)-, Butanedioic acid, diethyl ester, 1-Dodecanol, Ethanol, 2-(2-ethoxyethoxy)-and Maltol. Based on our knowledge this is the first-time metabolites from fungal endophytes of *Solanum mauritianum* Scop. have been analysed. The outer parts of the figures where the colours remain separate represent the metabolites that were discrete. Where the colours mix or merge this represents the metabolites found in two or more fungal endophytes which are similar.

When comparing the metabolites synthesized in summer and in winter, we observed that more metabolites were synthesised by the fungal endophytes in winter than in summer. It could be speculated that during harsh environmental conditions, endophytes tend to offer more protection and upregulate the hosts’ (plant) defence system. This phenomenon has been reported in a study on *Crocus sativus* by Wani et al. (2016), which explains the significant role played by endophytes in protecting the host plant against abiotic and biotic stresses in producing secondary metabolites of biological importance. Other authors have pointed out that fungal endophytes and their metabolites are also known to increase the host’s resistance to pathogens and drought, and to form a defence system to strengthen the host’s immunity and to maintain growth under stress (Katoch et al., 2017; Prabukumar et al., 2015). These results further provide insight into the relationships that occur between plants and their endophytes.

A combined total of 991 VOCs was observed from the GC-HRTOF-MS analysis of the eight crude extracts of fungal endophytes (Supplementary Table A.1). In this study we only focused on the volatile compounds that were similar across all eight fungal endophytes (Table 2) as depicted in the Venn diagrams Fig. 2a and b.

**Table 2:** Some of the similar volatile compounds that were obtained in winter and summer from eight fungal endophytes

Compounds	R.t. (min:sec)	*m/z*	MF	MC
*Winter volatile compounds*				
1,4-Benzenediamine, N-(1,3-dimethylbutyl)-N'-phenyl-	29:01		C_18_H_24_N_2_	Amine
1-Dodecanol	19:31	239,696	C_12_H_26_O	Fatty alcohol
2-Ethoxy-3-chlorobutane	04:07	1,139,739	C_6_H_13_ClO	Ether
2,4-Di-tert-butylphenol	20:06	1,249,951	C_14_H_22_O	Alkylphenols
3-Eicosene, (E)-	27:07	594,283	C_20_H_40_	Alkene
3-Ethoxy-1,1,1,7,7,7-hexamethyl-3,5,5-tris(trimethylsiloxy)tetrasiloxane	19:37	281,169	C_17_H_50_O_7_Si_7_	Siloxane
4H-Pyran-4-one, 2,3-dihydro-3,5-dihydroxy-6-methyl-	15:29	214,378	C_6_H_8_O_4_	Ester
7-Hexadecene, (Z)-	18:24	688,162	C_16_H_32_	Alkene
Acetyl chloride	10:30	15,211	C_2_H_3_CIO	Thioester
Benzoic acid, 4-iodo-2-(2-methyl-1-oxopropylamino)-	08:24	9913	C_11_H_12_INO_3_	Phenylone
Benzothiazole	16:31	1,768,340	C_7_H_5_NS	Heterobicyclic
Butane, 1-ethoxy-	03:49	71,840,113	C_6_H_14_O	Saturated Alkane
Benzeneethanamine, 2,5-difluoro-á,3,4-trihydroxy-N-methyl-	07:53	16,933	C_9_H_11_F_2_NO_3_	Amine
Butanedioic acid, diethyl ester	15:43	451,462	C_8_H_14_O_4_	Ester
Cyclotrisiloxane, hexamethyl-	17:12	140,639	C_6_H_18_O_3_Si_3_	Siloxane
Cyanogen chloride	11:24	13,532	CClN	Hydrocyanic acid
Dimethyl sulfone	13:11	27,026	C_2_H_6_O_2_S	Sulfones
Dimethyl Sulfoxide	08:17	12,030	C_2_H_6_OS	Amide
Dimethylsulfoxonium formylmethylide	07:09	16,778	C_4_H_8_O_2_S	Sulfur
Ethanol, 2-(2-ethoxyethoxy)-	13:49	6,366,698	C_6_H_14_O_3_	Alcohol
Maltol	15:05	130,647	C_6_H_6_O_3_	Ketone
Phenol, 2,5-bis(1,1-dimethylethyl)-	20:44	565,419	C_14_H_22_O	Phenolic
Pentachlorophenyl trans-crotonate	24:35	11,262	C_10_H_5_Cl_5_O_2_	Phenolic
S-Methyl methanethiosulphonate	14:31	1,579,982	C_2_H_6_O_2_S_2_	Sulfonic acid
*Summer volatile compounds*				
4,8,12,16-Tetramethylheptadecan-4-olide	21:27.02	246.1271	C_21_H_40_O_2_	Alkane hydrocarbon
Trichloromethane	3:07.97	117.9140	CHCl_3_	Alkane
Tridecanoic acid, methyl ester	21:09.52	227.2020	C_14_H_28_O_2_	FAME
Benzeneethanamine, 2-fluoro-á,3,4-trihydroxy-N-isopropyl-	26:19.94	176.1519	C_11_H_16_FNO_3_	Amine
Hexacosane	20:01.30	210.2341	C_26_H_54_	Acyclic alkane
Tetratetracontane	22:33.22	314.2602	C_44_H_90_	Acyclic alkane
2-methyloctacosane	27:30.90	378.0494	C_29_H_60_	Acyclic alkane
Octacosane	14:01.42	165.1024	C_28_H_58_	Acyclic alkane
Fumaric acid, ethyl 2,3,5-trichlorophenyl ester	10:06.88	331.0635	C_12_H_9_Cl_3_O_4_	FAME
Glutaric acid, di(but-3-en-2-yl) ester	17:43.51	179.1440	C_13_H_20_O_4_	FAME
2-Pentadecanone	17:04.89	249.9084	C_15_H_30_O	Ketone
Hexadecane	11:31.88	141.1643	C_16_H_34_	Alkane hydrocarbon
2,4-Decadienal, (E,E)-	8:16.43	152.1200	C_10_H_16_O	Fatty aldehyde
Eicosane	15:53.10	255.3004	C_20_H_42_	Acyclic alkanes

*R.t.* Retention time, *m/z* mass to charge ratio, *MF* Molecular formula, *MC* Molecular class

The volatile organic compounds observed in Table 2 were subsequently grouped into aliphatic and aromatic hydrocarbons, alcohols, amides, benzene derivatives, esters, fatty acids, ketones and phenols. Some of the VOCs have known biological and pharmaceutical importance, such as 1,4-benzenediamine, N-(1,3-dimethylbutyl)-N’-phenyl, an antioxidant that has the ability to biologically accumulate in tissues (Prosser et al., 2017) and can protect the plant from ozone (Manning et al., 2011). S-methyl methanethiosulphonate was reported by Makarov et al. (2019) as a novel reversible inhibitor of thiol-based activity in proteins, with the ability to alkylate proteins and enzymes with a thiol group, and to protect proteins without a thiol functional group from irreversible oxidation (Sun and Gou, 2012). 2-Ethoxy-3-chlorobutane is an abundant compound in Quranic plants mixtures and has been proven to have anti-inflammatory properties (Alam, 2015; Makarov et al., 2019).

In an experiment conducted by Gonzalez Audino et al. (2007), 1-dodecanol, a known insecticide, was added in a trial lotion to be used against resistant head lice and was reported to improve the lotion’s effectiveness against the resistant head lice *Pediculus humanus capitis* De Geer. 1-dodecanol was observed in our study, and was detected in all eight fungal endophytic extracts, as seen in Table 2. 3-Eicosene, (E)- was detected in *Calotropis procera* and has been proved to have anti-fungal properties against *Aspergillus fumigatus, A. niger*, *Microsporum canis*, *Microsporum fulvum* and *Trichophyton mentagrophytes* (Verma et al., 2012). 4H-Pyran-4-one, 2,3-dihydro-3,5-dihydroxy-6-methyl-, which was observed in all eight fungal endophytic extracts, is a phytol first detected in methanol extracts of *Citrus unshiu* leaves and is reported to have anticancer properties (Song et al., 2015). Benzothiazole is a bicyclic ring system with multiple applications such as anti-diabetic, analgesic, anti-convulsant, anti-inflammatory, anti-malarial, anti-microbial, anti-thelmintic and antitumour activities (Gill et al., 2015).

4,8,12,16-Tetramethylheptadecan-4-olide is a possible source of vitamin E (Rontani et al., 2007), and has antimicrobial activity (Osama et al., 2017) and anti-breast cancer properties, as reported by Swantara et al. (2019) in a study investigating the volatile compounds of the sponge *Xestospongia testudinaria* against HeLa cancer cells. The study demonstrates the significance of 4,8,12,16-Tetramethylheptadecan-4-olide as a possible anticancer drug, which was synthesised in this study by fungal endophytes *Aureobasidium pullulans*, *Paracamarosporium leucadendri*, *Cladosporium* sp., *Fusarium* sp., *Hyalodendriella* sp., *Penicillium chrysogenum*, *Penicillium chrysogenum* and *Talaromyces* sp. (Swantara et al., 2019).

Benzyl benzoate is an insect repellent and is reported to be one of the oldest drugs to treat scabies (Salavastru et al., 2017; Sharma et al., 2016). This was detected in the crude extracts of *A. pullulans*, *Cladosporium* sp., *Fusarium* sp*., Hyalodendriella* sp. and *P. chrysogenum* (L). Tetratetracontane (Table 2) was detected from all eight fungal endophytes and has been reported to have anti-candidal activity against *Candida albican* and *C. glabrata* (Ngo-Mback et al., 2019). Eicosane has been reported to have antifungal properties and skin regeneration and antibacterial properties (Chuah et al., 2018; Umaru et al., 2019). Eicosane also has anti-inflammatory and proliferation properties and antioxidants (Alsultan et al., 2019). 2-Pentadecanone has been reported to have skin regeneration and antibacterial properties (Siyambwa et al., 2019). Tridecanoic acid methyl ester was reported to have biological activity such as antibacterial and antifungal properties in a study conducted by Belakhdar et al. (2015). Hexacosane was identified in the *Sanseveria liberica* plant and has antibacterial activity (Rukaiyat et al., 2015). 2-methyloctacosane has antimicrobial properties against *A. flavus*, *C. albicans*, *Bacillus subtilis*, *Escherichia coli, Pseudomonas aeruginosa* and *Staphylococcus aureus* (Barretto & Vootla, 2018). A study conducted by Ntalli et al. (2016) reported that 2,4-decadienal, (E,E)- showed strong activity against *Meloidogyne arenaria, Meloidogyne incognita* and *Meloidogyne javanica* parasitic nematodes and promoted tomato growth. Based on our knowledge, this is the first time that volatile compounds produced by fungal endophytes from *S. mauritianum* Scop. have been reported. These volatile compounds are, moreover, bioactive and have been proven to have broad antimicrobial activity (Barretto & Vootla, 2018; Rukaiyat et al., 2015; Siyumbwa et al., 2019; Umaru et al., 2019).

### Comparison of volatile compounds from fungal endophytes

**Fig. 3:**
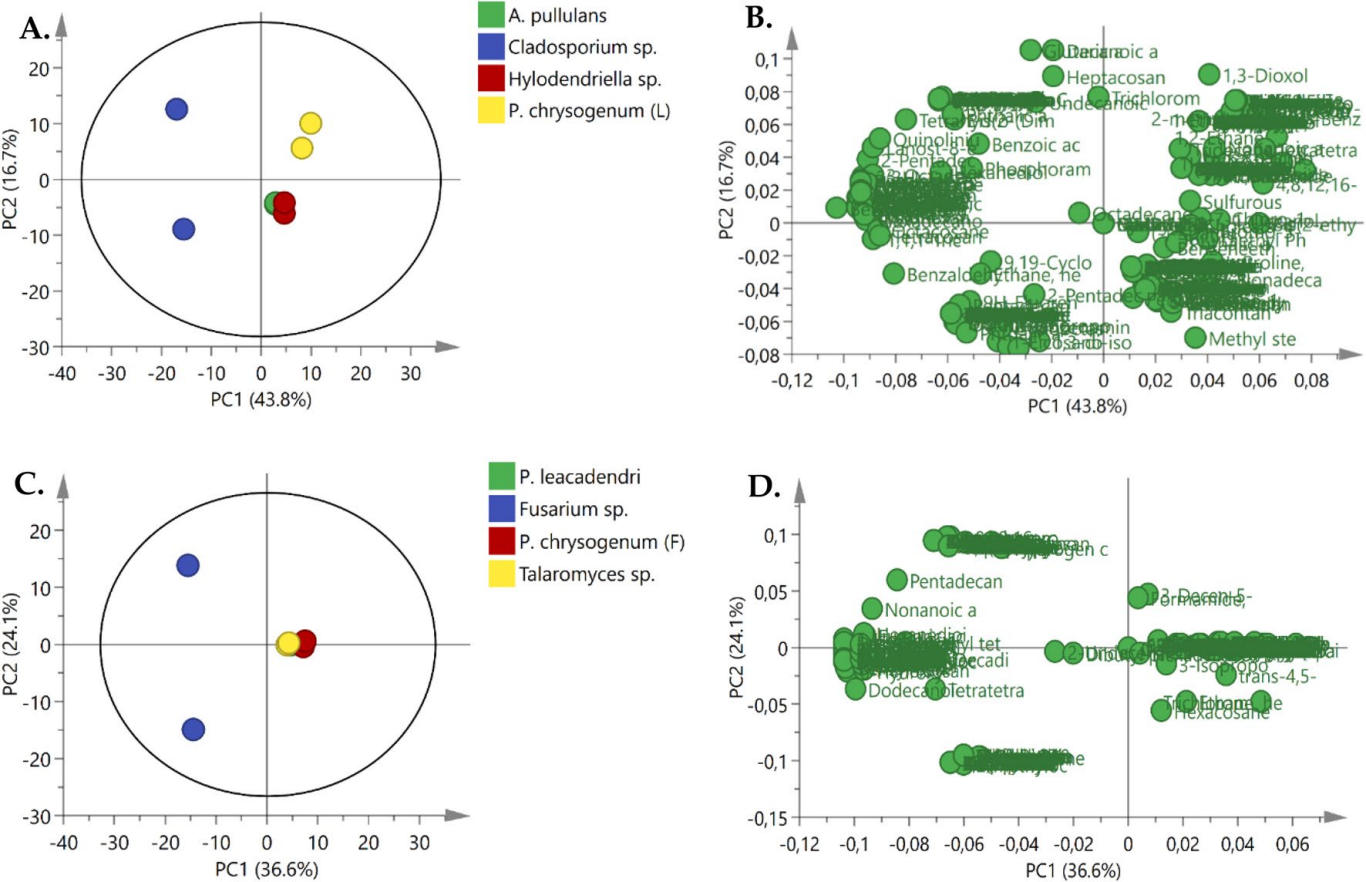
**a** PCA score plot of metabolites of fungal endophytes *A. pullulans*, *Cladosporium* sp*., Hyalodendriella* sp., and *P. chrysogenum* (L) extracts. **c** PCA score plot of metabolites of fungal endophytes *P. leucadendri*, *Fusarium* sp., *P. chrysogenum* **f** and *Talaromyces* sp. extracts. **b** PCA loadings of metabolites of fungal endophyte extracts with the least antimicrobial activity. **d** PCA loadings of metabolites of fungal endophyte extracts with the most antimicrobial activity

**Fig. 4:**
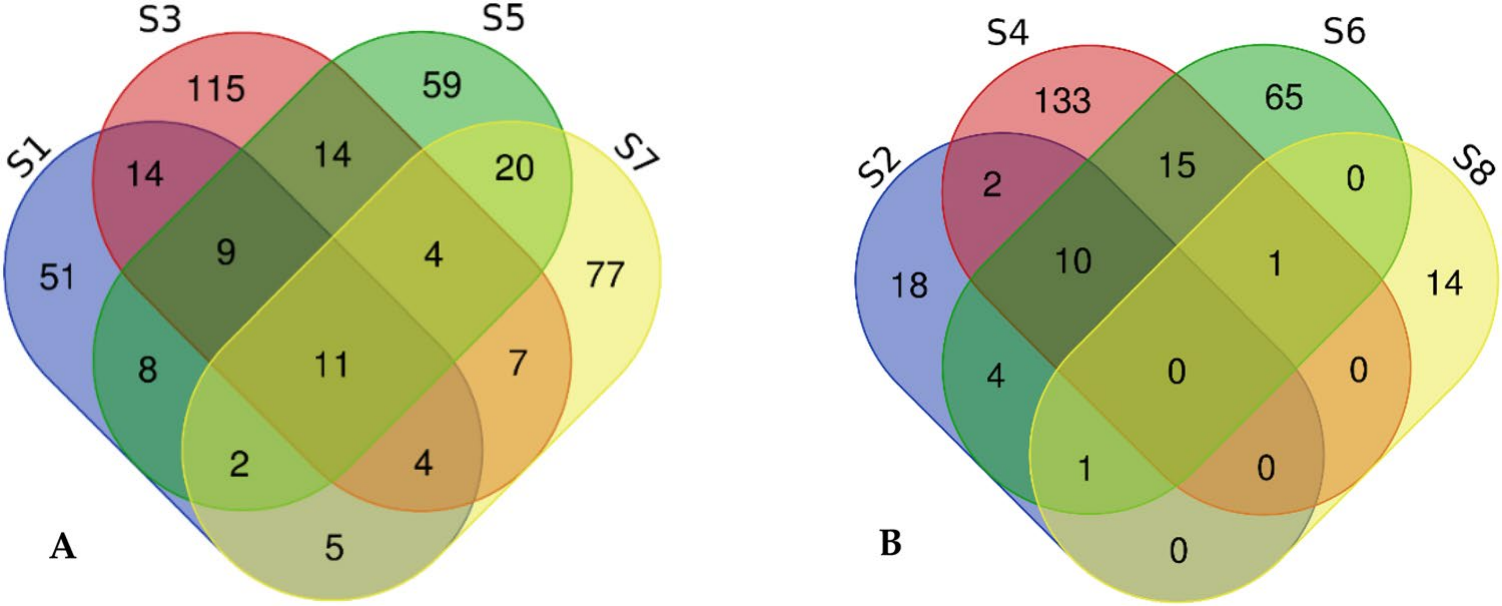
Venn diagram showing a comparison between fungal endophyte metabolites **a** S1—*Aureobasidium pullulans*, S3—*Cladosporium* sp., S5—*Hyalodendriella* sp. S7—*Penicillium chrysogenum* (L) and **b** demonstrates metabolites of S2—*Paracamarosporium leucadendri*; S4—*Fusarium* sp.; S6—*Penicillium chrysogenum* (fruits), and S8—*Talaromyces* sp

We further probed the metabolite distribution of the fungal endophytes *A. pullulans*, *Cladosporium* sp*., Hyalodendriella* sp., and *P. chrysogenum* (L) from the stem, ripe fruit, unripe fruit and leaves respectively, as observed in Fig. 3a and b. Different metabolites are distinctively separated from each other on the PCA score plot, with the PCA explaining 60.5% and 60.7%, respectively. The relatively close clusters of *A. pullulans* and *Hyalodendriella* sp. (Fig. 3a) suggest that these fungal endophytes share some common volatile compounds, in contrast to the volatile compounds of *Cladosporium* sp*.* and *P. chrysogenum* (L). The distribution of *Cladosporium* sp*.* was quite distinct, suggesting that the metabolites of this fungal endophyte might be quite different to the others. In a previous study we observed that *Cladosporium* sp. inhibited most of the pathogenic microorganisms at higher concentrations than *A. pullulans*, *Hyalodendriella* sp., and *P. chrysogenum* (L) (Pelo et al., 2020). This could be related to the number of unique metabolites (115) it has, as observed in Fig. 4a.

The PCA score plot showed the multivariate comparison of the fungal endophytes. The fungal endophytes showed inhibition against pathogenic microorganisms at concentrations below 10 mg/ml for *P. leucadendri*, *Fusarium* sp., *P. chrysogenum* (F) and *Talaromyces* sp. (Fig. 3c and d), compared to *A. pullulans*, *Cladosporium* sp*., Hyalodendriella* sp., and *P. chrysogenum* (L) (Fig. 3a and b), which inhibited pathogenic microorganism at concentrations above 10 mg/ml, as explained in our previous study (Pelo et al., 2020). The results showed a separation of sample clusters, suggesting different metabolites contributing to these variances, summarised in Fig. 3c. Significant metabolites contributed to the variation between the four fungal endophytes metabolites. *P. leucadendri*, *P. chrysogenum* (F) and *Talaromyces* sp. clustered, while *Fusarium* sp. scattered away from the other metabolites. *Fusarium* sp. reveals that the different metabolites have characteristics that are dissimilar to those that clustered together. The PCA loading plot shown in Fig. 3d shows the visualization of metabolites, contributing to the observed differences and similarities of grouping.

The Venn Diagram reveals the relationship of different endophytes isolated from different plant parts that play different or similar roles, and therefore some will synthesize similar metabolites. In Fig. 4a we observe that out of 302 metabolites synthesised by all four fungal endophytes, namely, *Aureobasidium pullulans*, *Cladosporium* sp., *Hyalodendriella* sp. and *Penicillium chrysogenum* (L), only 11 metabolites are similar. *Cladosporium* sp. had 115 metabolites, compared to the other three fungal endophytes, where *Aureobasidium pullulans* had 51 metabolites; *Hyalodendriella* sp. had 59 metabolites and *Penicillium chrysogenum* (L) had 77 metabolites. The 11 metabolites that were similar amongst all four fungal endophytes are tabulated in Table 3.

**Table 3:** Similar volatile compounds from *Aureobasidium pullulans*, *Cladosporium* sp., *Hyalodendriella* sp. and *Penicillium chrysogenum* (L)

Compounds	R.t (min:sec)	*m/z*	MF	MC
4,8,12,16-Tetramethylheptadecan-4-olide	21:27	246.1271		Alkane hydrocarbon
Benzeneethanamine, 2-fluoro-á,3,4-trihydroxy-N-isopropyl-	26:19	176.1519	C_11_H_16_FNO_3_	Amide
Benzyl Benzoate	15:36	212.0834	C_14_H_12_O_2_	Ester
Eicosane	15:53	255.3004	C20H42	Alkane
Fumaric acid, ethyl 2,3,5-trichlorophenyl ester	10:06	331.0635	C_12_H_9_Cl_3_O_4_	FAME
Glutaric acid, di(but-3-en-2-yl) ester	17:43	179.1440	C_13_H_20_O_4_	Ester
Hexacosane	20:01	210.2341	C_26_H_54_	Alkane
Hexadecane	11:31	141.1643	C_16_H_34_	Alkane
Octacosane	14:01	165.1024	C_28_H_58_	Acyclic alkane
Trichloromethane	3:07	117.9140	CHCl_3_	Alkane
Tridecanoic acid, methyl ester	21:09	227.2020	C_14_H_28_O_2_	FAME

*R.t.* Retention time, *m/z* mass to charge ratio, *MF* Molecular formula, *MC* Molecular class, *FAME* Fatty Acid Methyl Ester

*Fusarium* sp. (S4) synthesized 133 metabolites, which was more than the other three fungal endophytes combined, with *Paracamarosporium leucadendri* producing 18, *Penicillium chrysogenum* (fruit), 65 and *Talaromyces* sp., 14. We also observed that *Fusarium* sp. and *Penicillium chrysogenum* (F) from (ripe and unripe fruit, respectively) had 15 similar metabolites, *Paracamarosporium leucadendri*, and *Fusarium* sp. only two had similar metabolites, *Paracamarosporium leucadendri* and *Penicillium chrysogenum* (F) had 4 similar metabolites, *Penicillium chrysogenum* (F) and *Talaromyces* sp*.* had 1 similar metabolite, *Paracamarosporium leucadendri*, *Fusarium* sp. and *Penicillium chrysogenum* (F) had 10 similar metabolites, *Paracamarosporium leucadendri*, *Penicillium chrysogenum* (F) and *Talaromyces* sp*.* only had 1 similar metabolite, and *Fusarium* sp., *Penicillium chrysogenum* (F) and *Talaromyces* sp. also had 1 similar metabolite.

Table 3 shows the similar volatile compounds that were identified amongst *A. pullulans*, *Cladosporium* sp*., Hyalodendriella* sp., and *P. chrysogenum* (L), established in the Venn diagram in Fig. 4a.

When we compared the metabolites *A. pullulans*, *Cladosporium* sp*., Hyalodendriella* sp., and *P. chrysogenum* (L), about eleven metabolites were observed to be similar (Fig. 4a). Hexacosane is a volatile oil which plays a role in plant metabolites (PubChem, 2020a). Tridecanoic acid methyl ester is a fatty acid methyl ester that also plays a role in plant metabolites (PubChem, 2020). Some compounds with bioactivity are shown in Table 2, such as Glutaric acid, di(but-3-en-2-yl) ester; Eicosane, Fumaric acid, ethyl 2,3,5-trichlorophenyl ester, Hexadecane and Benzyl Benzoate.

## Conclusion

Eight fungal endophytes obtained from *Solanum mauritianum* Scop. were investigated in this study. Volatile metabolites were synthesized in different seasons and identified. Phytochemicals and volatile metabolites were found in the crude extracts of the fungal endophytes, with the most abundant being saponins and quinones. A number of volatile secondary metabolites were identified using GC-HRTOF-MS, with PCA assisting with an infographic grouping of the metabolites. In addition, some VOCs were identified to have a broad spectrum of biological activities, such as antibacterial, antifungal, anti-nematode, anti-inflammatory and antioxidant properties. Others were identified to be noteworthy potentials, for example, 1-dodecanol is an insecticide against the resistant head lice *Pediculus humanus capitis* De Geer, Benzyl benzoate is an insect repellent for scabies, 4H-Pyran-4-one, 2,3-dihydro-3,5-dihydroxy-6-methyl-, and 4,8,12,16-Tetramethylheptadecan-4-olide have anti-cancer properties and Benzothiazoles have anti-malarial properties. In conclusion, *Solanum mauritianum* Scop. possesses fungal endophytes with important volatile secondary metabolites which can be useful in the pharmaceutical industries and could be further researched for treatments for cancer and tuberculosis. Fungal endophytes are a key source for novel drugs.

## Supplementary Information

Below is the link to the electronic supplementary material.

Supplementary file1 (DOCX 228 kb)
