# Supplementary material for: Chemotaxonomic profiling of fungal endophytes of Solanum mauritianum (alien weed) using gas chromatography high resolution time-of-flight mass spectrometry (GC-HRTOF-MS)
Source: Metabolomics. 2021 Apr 20;17(5):43. doi: 10.1007/s11306-021-01790-7 (PMC8057964; doi:10.1007/s11306-021-01790-7)
Supplement: Supplementary file 1 — Supplementary file1 (DOCX 228 kb) [file 11306_2021_1790_MOESM1_ESM.docx]

Appendix

Table A.1: Compounds observed from the GC-HRTOF-MS.

| **R.T. (min:sec)** | ***m/z*** | **Name** | **Chemical Formula** | *Aureobasidium pullulans* | *Paracamarosporium leucadendri* | *Cladosporium* sp. | *Fusarium* sp. | *Hyalodendriella* sp. | *Penicillium chrysogenum* Fruit | *Penicillium chrysogenum* Leaves | *Talaromyces* sp. |
| --- | --- | --- | --- | --- | --- | --- | --- | --- | --- | --- | --- |
| 3:03 | 90.0632 | Mercaptoacetone | C_3_H_6_OS | ND | ND | ND | ND | ND | ND | ND | 50915 |
| 3:03 | 88.0520 | Ethyl Acetate | C_4_H_8_O_2_ | ND | ND | ND | ND | ND | ND | ND | 40731398 |
| 3:04 | 193.1013 | 4-Oxo-4-(para-tolyl)-butyric acid | C_11_H_12_O_3_ | ND | ND | ND | ND | ND | ND | ND | 355585 |
| 3:05 | 219.9887 | 2,4,4,6-Tetramethyl-6-phenyl-1-heptene | C_17_H_26_ | ND | ND | ND | ND | ND | ND | ND | 2626472 |
| 3:07 | 266.7170 | Indeno[3a,4-b]oxiren-2-ol, octahydro-4a-methyl-5-[(tetrahydro-2H-pyran-2-yl)oxy]-, (1aà,2á,4aá,5á,7aS)- | C_15_H_24_O_4_ | ND | ND | ND | ND | 27169356 | ND | ND | ND |
| 3:07 | 117.9141 | Trichloromethane | CHCl_3_ | 42828785 | 47752317 | 55500934 | 28319971 | 24778862 | 35470511 | 31721466 | ND |
| 3:08 | 131.8654 | Methane, oxybis[dichloro- | C_2_H_2_Cl_4_O | ND | ND | 34408529 | 27502350 | ND | ND | 102928 | ND |
| 3:09 | 226.9136 | Pyrrolidine-2-carboxamide, N-heptyl-N-methyl- | C_13_H_26_N_2_O | ND | ND | 15684 | ND | ND | ND | 7938460 | ND |
| 3:10 | 112.1248 | (E)-2-Octene | C_8_H_16_ | ND | ND | 11687559 | ND | ND | ND | ND | ND |
| 3:10 | 142.9029 | Propanoic acid, pentyl ester | C_8_H_16_O_2_ | ND | ND | ND | 13900156 | ND | ND | ND | ND |
| 3:10 | 125.8721 | Uracil, 1-methyl- | C_5_H_6_N_2_O_2_ | ND | ND | 4287988 | ND | ND | ND | ND | ND |
| 3:11 | 100.0881 | [2-Pyrrolidinyl]methylamine | C_5_H_12_N_2_ | ND | ND | ND | ND | ND | ND | 61250 | ND |
| 3:11 | 362.6985 | 3-Dimethyl(3-cyanopropyl)silyloxypentadecane | C_21_H_43_NOSi | 3384758 | ND | ND | ND | ND | ND | ND | ND |
| 3:15 | 220.9214 | Heptabarbital | C_13_H_18_N_2_O_3_ | ND | ND | ND | ND | ND | ND | 34433 | ND |
| 3:25 | 54.9377 | Ammonium Chloride | ClH_4_N | ND | ND | 5826809 | 60472184 | ND | ND | ND | ND |
| 3:26 | 74.0319 | 1-Penten-3-ol | C_5_H_10_O | ND | ND | ND | ND | ND | ND | ND | 26764179 |
| 3:26 | 109.9685 | dl-Alanine ethyl ester | C_5_H1_1_NO_2_ | ND | ND | ND | ND | ND | 3062982 | ND | ND |
| 3:26 | 75.0442 | 1-Nitroso-3,5-dinitro-hexahydro-1,3,5-triazine | C_3_H_6_N_6_O_5_ | ND | ND | ND | ND | ND | ND | ND | 9374576 |
| 3:28 | 124.9370 | 1,3-Cyclobutanediol, 2,2,4,4-tetramethyl- | C_8_H_16_O_2_ | ND | ND | ND | ND | ND | 366775 | ND | ND |
| 3:30 | 222.7938 | Manganese, acetylpentacarbonyl-, (OC-6-21)- | C_7_H_3_MnO_6_ | ND | ND | 10422705 | ND | ND | ND | ND | ND |
| 3:41 | 126.9423 | Propanedioic acid, dimethyl ester | C_5_H_8_O_4_ | ND | ND | ND | ND | ND | 3807729 | ND | ND |
| 3:59 | 97.1372 | Cyclopentane, 1,1-dimethyl- | C_7_H_14_ | ND | ND | 48640295 | ND | ND | ND | ND | ND |
| 4:00 | 83.0492 | 1-Methyl-1H-1,2,4-triazole | C_3_H_5_N_3_ | 101344 | ND | ND | ND | ND | ND | ND | ND |
| 4:01 | 129.8548 | 5-Chloro-5-methylnonane | C_10_H_21_Cl | ND | ND | ND | ND | ND | ND | 2926523 | ND |
| 4:01 | 125.9425 | N-[2-Cyanoethyl]-2-mercapto-2-methylpropanamine | C7H_14_N_2_S | 10002093 | ND | ND | ND | ND | ND | ND | ND |
| 4:01 | 145.4276 | 3-Octene, 2,2-dimethyl- | C_10_H_20_ | ND | 2345828 | ND | ND | ND | ND | ND | ND |
| 4:01 | 173.8441 | Cyclohexane, (2-nitro-2-propenyl)- | C_9_H_15_NO_2_ | ND | ND | 60721984 | ND | ND | ND | ND | ND |
| 4:01 | 95.0831 | 1-Pentyn-3-amine, 3-methyl- | C_6_H_11_N | ND | 790924 | ND | ND | ND | ND | ND | ND |
| 4:01 | 122.1092 | 1-Hexene, 3,5-dimethyl- | C_8_H_16_ | ND | ND | 38442245 | ND | ND | ND | ND | ND |
| 4:02 | 84.0570 | 1-Pentene, 3-methyl- | C_6_H_12_ | ND | ND | ND | 24595130 | ND | ND | ND | ND |
| 4:02 | 93.0336 | Cyclohexanone, 3,3,5-trimethyl- | C_9_H_16_O | ND | ND | ND | 23354600 | ND | ND | ND | ND |
| 4:02 | 47.0206 | 3-Methyl-1,2-diazirine | C_2_H_4_N_2_ | ND | ND | ND | ND | ND | ND | 4685465 | ND |
| 4:02 | 112.0885 | 2-Heptenal, (E)- | C_7_H_12_O | ND | ND | ND | ND | ND | 91651360 | ND | ND |
| 4:02 | 100.9964 | 1-[(1-Oxo-2-propenyl)oxy]-2,5-pyrrolidinedione | C_7_H_7_NO_4_ | ND | ND | ND | 148522 | ND | 26082530 | ND | ND |
| 4:11 | 166.8803 | 3-Pentanone, 2,2,4,4-tetramethyl- | C_9_H_18_O | 3771658 | ND | ND | ND | ND | ND | ND | ND |
| 4:13 | 292.8560 | Valylglycine, TMS derivative | C_10_H_22_N_2_O_3_Si | ND | ND | ND | ND | ND | 3252602 | ND | ND |
| 4:14 | 124.8752 | 2H-1,2-Oxazine, 6-(4-chlorophenyl)tetrahydro-2-methyl- | C11H14ClNO | ND | ND | ND | ND | ND | ND | 1053166 | ND |
| 4:14 | 414.9802 | Dipivefrine, N.O-bis(pentafluoropropionyl)- | C_25_H_27_F_10_NO_7_ | ND | ND | 105816274 | ND | ND | 42558103 | ND | ND |
| 4:24 | 139.1074 | (3S,5R,8aR)-3-Butyl-5-methyloctahydroindolizine | C_13_H_25_N | ND | ND | ND | ND | 453115 | ND | ND | ND |
| 4:24 | 71.0128 | Furan, 2-(chloromethyl)tetrahydro- | C_5_H_9_ClO | 27875 | ND | ND | ND | ND | ND | ND | ND |
| 4:24 | 138.1041 | Furan, 2-pentyl- | C_9_H_14_O | ND | ND | ND | 12475071 | 4088721 | ND | ND | ND |
| 4:27 | 295.6927 | Methanone, [4-(2-furfurylthio)-3-nitrophenyl](morpholino)- | C_16_H_16_N_2_O_5_S | 796035 | ND | ND | 556096 | ND | ND | ND | ND |
| 4:28 | 137.0959 | 1-[4-(4-tert-Butyl-spirocyclohexyl)-11-oxa-3-aza-tricyclo[6.2.1.0(1,6)]undec-9-en-3-yl]-2,2,2-trifluoro-ethanone | C_20_H_28_F_3_NO_2_ | ND | ND | 8556266 | ND | 1189073 | ND | ND | ND |
| 4:28 | 125.0959 | 2-(3-methyl-2-cyclopenten-1-yl)-2-methylpropionaldehyde | C1_0_H_16_O | ND | ND | ND | ND | ND | ND | 2408993 | ND |
| 4:31 | 84.0934 | 2,3-Pentanedione | C_5_H_8_O_2_ | ND | ND | ND | ND | ND | ND | ND | 1054953 |
| 4:33 | 313.9832 | Chlorfenapyr | C1_5_H_11_BrClF_3_N_2_O | ND | ND | ND | ND | 797348 | ND | ND | ND |
| 4:34 | 270.8794 | Furoxan, 3,4-bis(2-thienylcarbonyl)- | C_12_H_6_N_2_O_4_S_2_ | ND | ND | 10275 | ND | ND | ND | ND | ND |
| 4:35 | 151.8818 | Hexan-3-yl (E)-2-methylbut-2-enoate | C_11_H_20_O_2_ | ND | ND | ND | ND | ND | 1261940 | ND | ND |
| 4:38 | 100.0884 | p-Dioxin, 2,3-dihydro-5-methyl- | C_5_H_8_O_2_ | ND | ND | ND | 59750 | ND | ND | ND | ND |
| 4:53 | 128.8695 | Butanoic acid, 2-cyano-3-methyl-, ethyl ester | C_8_H_13_NO_2_ | ND | ND | ND | ND | 111657 | ND | ND | ND |
| 4:54 | 255.8893 | Meta-Methoxymethcathinone | C_11_H_15_NO_2_ | ND | ND | ND | ND | ND | 3378320 | ND | ND |
| 4:56 | 140.1199 | 1,2-Ethanediol, 1,2-diphenyl-, [R-(R*,R*)]- | C1_4_H_14_O_2_ | ND | ND | ND | ND | ND | ND | 942110 | ND |
| 4:56 | 126.1040 | 3,5-Octadien-2-ol | C_8_H_14_O | ND | ND | 11834376 | ND | ND | ND | ND | ND |
| 4:56 | 135.9425 | 1,2,4-Triazole, 5-cyclohexanecarboxamido- | C_9_H_14_N_4_O | ND | ND | ND | ND | 2079832 | ND | ND | ND |
| 4:57 | 128.8695 | 3,3-Diethoxy-1-propyne | C_7_H_12_O_2_ | ND | ND | ND | ND | 2305368 | ND | ND | ND |
| 4:57 | 108.0571 | 4-(N-Methylamino)pyridine | C_6_H_8_N_2_ | ND | ND | ND | ND | ND | ND | 913488 | ND |
| 4:57 | 117.0698 | 3-Octen-2-one | C_8_H_14_O | 536756 | ND | ND | ND | ND | ND | ND | ND |
| 4:57 | 201.7902 | Cyclopentanecarboxamide, N-(2-fluorophenyl)- | C1_2_H_14_FNO | ND | ND | 6061657 | ND | ND | ND | ND | ND |
| 4:57 | 126.1040 | 3-Octen-2-one, (E)- | C_8_H_14_O | ND | ND | ND | ND | ND | 14473976 | ND | ND |
| 4:57 | 92.0253 | 2-Propenoic acid, ethenyl ester | C_5_H_6_O_2_ | ND | ND | ND | 20399203 | ND | ND | ND | ND |
| 4:57 | 136.8770 | 2-Pyrrolidinone, 1-(1-oxo-2-propenyl)- | C_7_H_9_NO_2_ | ND | ND | ND | 18470011 | ND | ND | ND | ND |
| 4:58 | 222.7942 | 2-Heptenoic acid, 4-nitrophenyl ester | C_13_H_15_NO_4_ | 513746 | ND | ND | ND | ND | 22153282 | ND | ND |
| 5:01 | 218.9179 | (2-Methyl[1,3]dioxolan-2-yl)thioacetic acid, S-[3-(2-methyl[1,3]dioxolan-2-yl)-2-oxopropyl] ester | C_13_H_20_O_6_S | ND | ND | ND | ND | ND | 15918 | 149769 | ND |
| 5:02 | 121.0613 | 2-Isopropyl-5-methylcyclohexyl 3-(1-(4-chlorophenyl)-3-oxobutyl)-coumarin-4-yl carbonate | C_30_H_33_ClO_6_ | ND | ND | 870537 | ND | ND | ND | ND | ND |
| 5:05 | 113.0588 | 4-(Methylamino)butyric acid | C_5_H_11_NO_2_ | ND | ND | ND | ND | 71953 | ND | ND | ND |
| 5:05 | 187.8254 | d,l-trans-4-Methyl-5-methoxy-1-(1-methoxy-1-isopropyl)cyclohex-3-ene | C_12_H_22_O_2_ | ND | ND | 1069435 | ND | ND | ND | N D | ND |
| 5:10 | 135.9439 | 2-Nonen-4-one | C_9_H_16_O | 217316 | ND | ND | ND | ND | ND | ND | ND |
| 5:11 | 113.0235 | 2,3-Dimethyl-1-hexene | C_8_H_16_ | ND | ND | ND | 5701664 | ND | ND | ND | ND |
| 5:11 | 294.9850 | N-[á-Hydroxy-á-[4-[1-adamantyl-6,8-dichloro]quinolyl]ethyl]piperidine | C_26_H_32_Cl_2_N_2_O | ND | ND | 1058567 | ND | ND | ND | ND | ND |
| 5:11 | 165.8361 | (E)-2-(2-Methoxyethoxy)ethyl 2-methylbut-2-enoate | C_10_H_18_O_4_ | ND | ND | ND | 6536366 | ND | ND | ND | ND |
| 5:12 | 121.0648 | 2-Hexanone, 6-hydroxy- ; 6-Hydroxy-hexan-2-one | C_6_H_12_O_2_ | ND | ND | 23357 | ND | ND | ND | ND | ND |
| 5:13 | 89.0388 | Hydroxylamine, O-(2-methylpropyl)- | C_4_H_11_NO | ND | ND | ND | ND | ND | 18882403 | ND | ND |
| 5:13 | 104.0389 | Propane, 2-methyl-1-nitro- | C_4_H_9_NO_2_ | ND | ND | ND | ND | ND | ND | ND | ND |
| 5:15 | 99.0806 | Propanoic acid, ethenyl ester | C_5_H_8_O_2_ | 890044 | ND | ND | ND | ND | ND | ND | ND |
| 5:16 | 126.0674 | Propane, 1-(chloromethoxy)-2-methyl- | C_5_H_11_ClO | ND | ND | ND | ND | ND | ND | 625212 | ND |
| 5:23 | 85.0523 | Phosphoramidous difluoride | F_2_H_2_NP | ND | ND | ND | ND | ND | ND | ND | ND |
| 5:26 | 195.8772 | Acetic acid, ester with trichloroethanol | C_4_H_5_Cl_3_O_2_ | ND | ND | ND | 308580 | ND | ND | ND | ND |
| 5:27 | 214.9931 | Ethane, hexachloro- | C_2_Cl_6_ | 780288 | 612516 | 533568 | 404320 | 13890 | 450636 | ND | ND |
| 5:27 | 163.8749 | Ethene, tetrachloro- | C_2_Cl_4_ | ND | ND | ND | ND | ND | 197537 | ND | ND |
| 5:28 | 135.0624 | 2-Butenedioic acid (Z)-, dimethyl ester | C_6_H_8_O_4_ | ND | ND | ND | ND | 265102 | ND | ND | ND |
| 5:32 | 254.0181 | Acetic acid, [4-(2,3-dihydro-1-indolylcarbonyl)]phenyl ester | C_17_H_15_NO_3_ | ND | ND | 211853 | ND | ND | ND | ND | ND |
| 5:34 | 99.0680 | 1-Pyrrolidinecarboxaldehyde | C_5_H_9_NO | ND | ND | ND | ND | ND | ND | 401649 | ND |
| 5:34 | 121.0649 | 1-(3-Chloro-4-fluoro-phenyl)-5-(4-methoxy-benzylsulfanyl)-1H-tetrazole | C_15_H_12_ClFN_4_OS | ND | ND | ND | ND | 36556 | ND | ND | ND |
| 5:35 | 129.0696 | 3-Formylamino succinimide | C_5_H_6_N_2_O_3_ | ND | ND | ND | ND | ND | ND | ND | ND |
| 5:36 | 123.0800 | 3-Chloropropionic acid, 2-tetrahydrofurylmethyl ester | C_8_H_13_ClO_3_ | ND | ND | ND | ND | 141330 | ND | ND | ND |
| 5:36 | 220.9213 | N-{2-(2-Chlorophenyl)-2-[(trimethylsilyl)oxy]ethyl}propan-2-amine | C_14_H_24_ClNOSi | ND | ND | ND | ND | ND | 268252 | ND | ND |
| 5:36 | 253.8870 | Pregnan-18-ol, 20-methyl-20-(methylamino)-, (5à)- | C_23_H_41_NO | ND | ND | ND | 200777 | ND | ND | ND | ND |
| 5:37 | 230.9854 | 3-Pentenoic acid, 2-hydroxy-4-methyl-2-(2-methyl-2-propenyl)-, ethyl ester | C1_2_H_20_O_3_ | 249503 | ND | ND | ND | ND | ND | ND | ND |
| 5:39 | 146.0391 | N,N-dimethyl-4-tert-butylamphetamine | C_15_H_25_N | ND | ND | ND | 42247 | ND | ND | ND | ND |
| 5:39 | 169.9917 | 2-Cyclohexylthiacyclohexane | C_11_H_2_0S | ND | ND | ND | 26527 | ND | ND | ND | ND |
| 5:39 | 146.0391 | 2,4-Dioxabicyclo[3.2.0]hept-6-en-3-one, 1,5-dichloro-6,7-dimethyl- | C_7_H_6_Cl_2_O_3_ | ND | ND | ND | 48117 | ND | ND | ND | ND |
| 5:39 | 147.0658 | N,N-Diethyl-N'-formyl-N'-methoxyurea | C_7_H_14_N2O_3_ | ND | ND | 129804 | ND | ND | ND | ND | ND |
| 5:39 | 143.1029 | 3-Methyl-2-butenoic acid, 2-tetrahydrofurylmethyl ester | C_10_H_16_O_3_ | ND | ND | ND | ND | ND | 1692277 | ND | ND |
| 5:43 | 138.8744 | Nonanal | C_9_H_18_O | 219106 | ND | ND | ND | ND | ND | ND | ND |
| 5:44 | 201.7888 | 3,8-Decanedione | C_10_H_18_O_2_ | ND | ND | ND | 1139569 | ND | ND | ND | ND |
| 5:44 | 129.0541 | 3,4-Dihydroxymethamphetamine | C_10_H_15_NO_2_ | ND | ND | ND | ND | ND | ND | 11736 | ND |
| 5:44 | 124.1246 | 8-Chloro-6-[2-piperidino-1-hydroxyethyl]-2-phenylquinoline | C_22_H_23_ClN_2_O | ND | ND | ND | ND | ND | 226584 | ND | ND |
| 5:44 | 133.9404 | 5-(Hydroxymethyl)-1,2-oxazole-3-carboxamide | C_5_H_6_N_2_O_3_ | 207612 | ND | ND | ND | ND | ND | ND | ND |
| 5:44 | 275.9860 | 1H-1,2-Dithiolo[3,4-c]quinoline-1-thione, 4,5-dihydro-8-ethoxy-4,4-dimethyl-5-[1-oxo-2-(1-piperidyl)ethyl]- | C_21_H_26_N2O_2_S_3_ | ND | ND | 467088 | ND | ND | ND | ND | ND |
| 5:45 | 220.9212 | 1,2-Dihydro-3-methoxy-2-oxo-1-piperidinomethylpyridine | C_12_H_18_N_2_O_2_ | ND | ND | ND | ND | 52796 | ND | ND | ND |
| 5:47 | 184.0317 | Furane-2-carboxylic acid, 5-(3-chlorophenoxymethyl)- | C_12_H_9_ClO_4_ | ND | ND | 1628 | ND | ND | ND | ND | ND |
| 5:49 | 273.8819 | Zygadenine 3-acetate | C_29_H_45_NO_8_ | ND | ND | ND | ND | ND | ND | ND | ND |
| 5:50 | 184.0318 | 4-(2-Acetoxyphenyl)-1-ethyl-3-methyl-5-(4-nitrophenyl)pyrazole | C_20_H_19_N_3_O_4_ | ND | ND | ND | ND | ND | ND | 7460680 | ND |
| 5:55 | 123.0800 | (R)-(+)-1-Amino-2-(methoxymethyl)pyrrolidine | C_6_H_14_N_2_O | ND | ND | ND | ND | 538747 | ND | ND | ND |
| 5:55 | 304.8492 | 2H-Pyran, 2-[[(5à,17á)-androstan-17-yl]oxy]tetrahydro- | C_24_H_40_O_2_ | ND | ND | ND | ND | ND | ND | 746615 | ND |
| 5:55 | 101.0234 | 1-Butanol, 3,3-dimethyl- | C_6_H_14_O | ND | ND | ND | ND | ND | ND | 188893 | ND |
| 5:55 | 217.9189 | 3-Methylpentan-3-yl 2-methylbutanoate | C_11_H_22_O_2_ | ND | ND | ND | ND | 523934 | ND | ND | ND |
| 5:56 | 122.0727 | Hydrazine, (phenylmethyl)- | C_7_H_10_N_2_ | ND | ND | ND | ND | ND | ND | ND | 752843 |
| 5:57 | 122.0727 | Phenylethyl Alcohol | C_8_H_10_O | ND | ND | ND | ND | ND | ND | ND | 342472 |
| 5:57 | 127.1119 | Heptanoic acid, 6-methyl, methyl ester | C_9_H_18_O_2_ | ND | ND | ND | ND | 12125 | ND | ND | ND |
| 5:58 | 104.0620 | Dodecanoic acid, 2-methyl- | C_13_H_26_O_2_ | 65510 | ND | ND | 165068 | ND | ND | ND | ND |
| 5:58 | 135.9407 | Dodecanoic acid, 2-methyl- ; 2-Methyldodecanoic acid | C_13_H_26_O_2_ | 78528 | ND | ND | ND | ND | ND | ND | ND |
| 5:59 | 150.9934 | Octanoic acid, methyl ester | C_9_H_18_O_2_ | ND | ND | 653452 | ND | ND | ND | ND | ND |
| 6:01 | 129.9139 | Hexanoic acid, methyl ester | C_7_H_14_O_2_ | ND | ND | ND | 915072 | ND | ND | ND | ND |
| 6:02 | 303.6830 | 2-Thiophenecarboxamide, N-octyl-N-(2-thiophenecarbonyl)- | C_18_H_23_NO_2_S_2_ | ND | ND | ND | ND | ND | 883467 | ND | ND |
| 6:02 | 126.0675 | Thiophene-2-carboxylic acid, 2,5-dichlorophenyl ester | C_11_H_6_Cl_2_O_2_S | ND | ND | ND | ND | ND | 744511 | 183485 | ND |
| 6:13 | 152.0833 | L-Mannitol, 1-deoxy-, cyclic 3,4-(ethylboronate) 2,5,6-triacetate | C_14_H_23_BO_8_ | ND | ND | ND | ND | 88018 | 305309 | 48100 | ND |
| 6:13 | 125.0961 | 1H-Pyrazole, 4-(trimethylsilyl)- ; 4-(Trimethylsilyl)pyrazole | C_6_H_12_N_2_Si | ND | ND | 364981 | ND | ND | ND | ND | ND |
| 6:13 | 176.9965 | Furan, 2-(1,2-diethoxyethyl)- | C_10_H_16_O_3_ | ND | ND | 420456 | ND | ND | ND | ND | ND |
| 6:14 | 220.9200 | Glycine, N-methyl-N-methoxycarbonyl-, hexadecyl ester | C_21_H_41_NO_4_ | ND | ND | ND | 505582 | ND | ND | ND | ND |
| 6:14 | 327.0352 | Glycine, N-methyl-N-methoxycarbonyl-, undecyl ester | C_16_H_31_NO_4_ | ND | ND | ND | 346793 | ND | ND | ND | ND |
| 6:16 | 308.8512 | 3-Methyl-2-butenoic acid, tridec-2-ynyl ester | C_18_H_30_O_2_ | ND | ND | ND | ND | ND | 247353 | ND | ND |
| 6:17 | 357.0689 | Cyclopentasiloxane, decamethyl- | C_10_H_30_O_5_Si_5_ | 50460 | ND | ND | ND | ND | ND | ND | ND |
| 6:18 | 266.9992 | Nicotinic acid, 3,4-dichlorophenyl ester | C_12_H_7_Cl_2_NO_2_ | ND | ND | 60703 | ND | ND | ND | ND | ND |
| 6:19 | 137.0481 | Propan-2-ol, 1-[1-(2,3-dihydrobenzo[1,4]dioxin-2-yl)ethoxy]-3-(piperidin-1-yl)- | C_18_H_27_NO_4_ | ND | ND | ND | ND | ND |  | ND | ND |
| 6:22 | 144.0419 | 2,5-Difluoroanisole | C_7_H_6_F_2_O | ND | ND | 41951 | ND | ND | ND | ND | ND |
| 6:24 | 103.0389 | Butylboronic acid | C_4_H_11_BO_2_ | ND | ND | ND | ND | ND | 717850 | ND | ND |
| 6:24 | 73.0285 | Formamide, N,N-dimethyl- | C_3_H_7_NO | ND | ND | ND | ND | ND | 465871 | ND | ND |
| 6:26 | 143.0939 | Thiophene-2-carboxylic acid,N'-[2-(4-chlorophenoxy) acetyl]hydrazide | C_13_H_11_ClN_2_O_3_S | ND | ND | 42336 | ND | DN | ND | ND | ND |
| 6:27 | 99.0680 | N-Methyl-à-pyrrolidone | C_5_H_9_NO | ND | ND | ND | ND | 265187 | ND | ND | ND |
| 6:28 | 128.0704 | 2-Pyrrolidinemethanamine, 1-ethyl- | C_7_H_16_N_2_ | ND | ND | ND | ND | ND | ND | 216449 | ND |
| 6:28 | 150.1040 | 3-(4-Hydroxyphenyl)propanal | C_9_H_10_O_2_ | ND | ND | ND | 165119 | ND | ND | ND | ND |
| 6:29 | 149.1281 | Benzeneethanamine, N-(1-methylethylidene)- | C_11_H_15_N | ND | ND | ND | ND | ND | 584397 | ND | ND |
| 6:29 | 151.1074 | Glycine, N-benzyloxycarbonyl-N-ethyl-, propyl ester | C_15_H_21_NO_4_ | ND | ND | ND | ND | ND | 406679 | ND | ND |
| 6:30 | 129.0707 | Cyclobutane, 1,1,2,3,3-pentamethyl- | C_9_H_18_ | ND | ND | 978279 | ND | ND | ND | ND | ND |
| 6:31 | 150.1036 | Sulfoxide, methyl phenethyl | C_9_H_12_OS | ND | ND | ND | ND | 65829 | ND | ND | ND |
| 6:31 | 155.1067 | 2,6-Difluorobenzoic acid, oct-3-en-2-yl ester | C_15_H_18_F_2_O_2_ | ND | ND | ND | ND | ND | ND | 164880 | ND |
| 6:32 | 156.1136 | Acetic acid, 3-acetoxy-1-ethyl-2-nitrobutyl ester | C_10_H_17_NO_6_ | ND | ND | ND | ND | ND | ND | 814587 | ND |
| 6:35 | 199.9868 | Thiophene, 2-ethyl-5-(2-methylpropyl)- | C_10_H_16_S | ND | ND | ND | ND | 21984 | ND | ND | ND |
| 6:36 | 126.0502 | Isopropyl(trimethylsilyl)acetylene | C_8_H_16_Si | ND | ND | ND | ND | 33472 | ND | ND | ND |
| 6:38 | 127.0756 | L-2-Aminobutyric acid, N-dimethylaminomethylene-, butyl ester | C_11_H_22_N_2_O_2_ | ND | ND | ND | ND | ND | ND | ND | ND |
| 6:39 | 142.0444 | Diglycolic acid, isohexyl 2-isopropylphenyl ester | C_19_H_28_O_5_ | ND | ND | ND | ND | ND | ND | ND | ND |
| 6:39 | 142.0439 | Benzene, [1-[[1-(1-methylethyl)-3-butenyl]oxy]ethyl]-, [S-(R*,R*)]- | C_15_H_22_O | ND | ND | ND | ND | 86055 | ND | ND | ND |
| 6:40 | 213.9375 | Vardenafil | C_23_H_32_N_6_O_4_S | ND | ND | ND | ND | ND | 146126 | ND | ND |
| 6:41 | 150.1403 | Propyl Ethylphosphonofluoridate | C_5_H_12_FO_2_P | ND | ND | 41916 | ND | ND | ND | ND | ND |
| 6:42 | 221.0794 | Benserazide | C_10_H_15_N_3_O_5_ | ND | ND | ND | ND | ND | ND | 446768 | ND |
| 6:42 | 197.0343 | Methyl d-glycero-á-d-gulo-heptoside | C_8_H_16_O_7_ | ND | ND | ND | ND | ND | ND | ND | ND |
| 6:44 | 142.9915 | 1,2-Ethanediol, dipropanoate | C_8_H_14_O_4_ | ND | ND | 1168971 | ND | ND | ND | ND | ND |
| 6:46 | 122.0364 | 4-Pyridinecarboxamide | C_6_H_6_N_2_O | ND | ND | ND | ND | ND | ND | 61720 | ND |
| 6:46 | 136.0883 | Oxirane, (bromomethyl)- | C_3_H_5_BrO | ND | ND | ND | ND | ND | 1332502 | ND | ND |
| 6:46 | 160.9087 | Ethylene dipropionate | C_8_H_14_O_4_ | ND | ND | ND | ND | ND | 1526103 | ND | ND |
| 6:47 | 363.9799 | 7-Chloro-1,3,4,10-tetrahydro-10-hydroxy-1-[[2-[1-pyrrolidinyl]ethyl]imino]-3-[3-(trifluoromethyl)phenyl]-9(2H)-acridinone | C_26_H_25_ClF_3_N_3_O_2_ | ND | ND | ND | ND | 187208 | 58095 | 235803 | ND |
| 6:47 | 150.0672 | 6,8-Dichloro-1,2,3,4-tetrahydro-2-methyl-4-[hydroxy-[2-hexahydropyridyl]methyl]quinoline | C_16_H_22_Cl_2_N_2_O | ND | ND | ND | ND | 186952 | ND | 283107 | ND |
| 6:48 | 138.0673 | Dibenzoyl-L-tartaric acid | C_18_H_14_O_8_ | ND | ND | ND | ND | ND | ND | 55236 | ND |
| 6:50 | 169.1907 | Acetamide, 2,2,2-trifluoro-N-(1-methyl-1H-imidazol-2-yl)- | C_6_H_6_F_3_N_3_O | ND | ND | 404907 | ND | ND | ND | ND | ND |
| 6:51 | 98.1092 | 1-Pentyn-1-ol, 4-methyl- | C_6_H_10_O | ND | ND | 1796846 | ND | ND | ND | ND | ND |
| 6:56 | 88.0075 | Phosphorus trifluoride | F_3_P | ND | ND | 635581 | ND | ND | ND | ND | ND |
| 6:56 | 147.0658 | 2-(Trimethylsilylperoxy)oxolane | C_7_H_16_O_3_Si | ND | ND | ND | ND | 364037 | ND | ND | ND |
| 6:56 | 155.1067 | trans-Verbenyl decanoate | C_20_H_34_O_2_ | ND | ND | ND | ND | ND | ND | 1560231 | ND |
| 6:56 | 145.0781 | Trimethyl(tetrahydrofuran-2-ylperoxy)silane | C_7_H_16_O_3_Si | ND | ND | ND | ND | 405008 | ND | ND | ND |
| 6:58 | 170.2028 | Dichloroacetic acid, 2,2-dimethylpropyl ester | C_7_H_12_Cl_2_O_2_ | ND | ND | 1823311 | ND | ND | 1148261 | ND | ND |
| 6:58 | 100.1197 | Pentane, 3,3-dimethyl- | C_7_H_16_ | ND | ND | 2645730 | ND | ND | ND | ND | ND |
| 6:58 | 139.1112 | 6-Hepten-2-one, 5,7,7-trichloro- | C_7_H_9_Cl_3_O | ND | ND | ND | ND | ND | 905306 | ND | ND |
| 6:58 | 161.8413 | Valeric acid, 2-tetrahydrofurylmethyl ester | C_10_H_18_O_3_ | ND | ND | ND | ND | ND | 272491 | ND | ND |
| 6:58 | 199.7913 | Ethylenediamine, N,N'-dimethyl-N-phenyl-N'-p-tolyl- | C_17_H_22_N_2_ | ND | ND | ND | ND | ND | ND | 276514 | ND |
| 7:04 | 141.0782 | Decanal | C_10_H_20_O | ND | ND | ND | ND | 376959 | ND | N D | ND |
| 7:05 | 152.0831 | 4-(7-Methoxy-7-methyloxepan-2-ylidene)butan-2-one | C_12_H_20_O_3_ | ND | ND | ND | ND | ND | 198416 | ND | ND |
| 7:05 | 137.0934 | 3-Methyl-2,4,10-trioxatricyclo[3.3.1.1alanan3,7alanan]decane peak 2 | C_8_H_12_O_3_ | ND | ND | ND | ND | ND | ND | 4227136 | ND |
| 7:10 | 69.0700 | 2-Propynenitrile, 3-fluoro- | C_3_FN | 56825 | ND | ND | ND | ND | ND | ND | ND |
| 7:10 | 119.0492 | 2-Propenoic acid, 2-methyl-, octyl ester | C_12_H_22_O_2_ | 66098 | ND | ND | ND | ND | ND | ND | ND |
| 7:14 | 153.1020 | Carbamic acid, (2,6-dichlorophenyl)-, 2-furanylmethyl ester | C_12_H_9_Cl_2_NO_3_ | 224117 | ND | 7418027 | ND | ND | ND | ND | ND |
| 7:14 | 82.0414 | Fomepizole | C_4_H_6_N_2_ | ND | ND | ND | ND | ND | ND | 417229 | ND |
| 7:14 | 138.1041 | 2,4-Nonadienal | C_9_H_14_O | ND | ND | ND | ND | ND | 6199650 | ND | ND |
| 7:14 | 120.0935 | 2,4-Octadienal, (E,E)- | C_8_H_12_O | ND | ND | ND | ND | ND | 5291553 | ND | ND |
| 7:15 | 185.0992 | Acetamide, N-(5-chloro-2-methoxyphenyl)-2-(4-methyl-1-piperidinyl)- | C_15_H_21_ClN_2_O_2_ | ND | ND | 39825 | ND | ND | ND | ND | ND |
| 7:16 | 134.1093 | Dimethyl fumarate | C_6_H_8_O_4_ | ND | ND | ND | 24064 | ND | ND | ND | ND |
| 7:18 | 142.9897 | 2-Butyldimethylsilyloxybut-3-yne | C_10_H_20_OSi | ND | ND | 464942 | ND | ND | ND | ND | ND |
| 7:18 | 138.0674 | Tetradonium Bromide | C_17_H_38_BrN | ND | ND | ND | ND | ND | ND | 411841 | ND |
| 7:18 | 141.1273 | 3,5,5-Trimethylhexanal | C_9_H_18_O | ND | ND | ND | ND | ND | 504798 | ND | ND |
| 7:20 | 140.0789 | Cyclohexanecarboxylic acid, 2-tetrahydrofurylmethyl ester | C_12_H2_0_O_3_ | ND | ND | ND | ND | ND | ND | 464978 | ND |
| 7:20 | 139.0754 | 3-t-Pentylcyclopentanone | C_10_H_18_O | ND | ND | ND | ND | ND | ND | 374921 | ND |
| 7:23 | 120.0571 | Benzofuran, 2,3-dihydro- | C_8_H_8_O | ND | ND | ND | 1089138 | ND | ND | ND | ND |
| 7:25 | 128.0709 | Proline ethyl ester | C_7_H_13_NO_2_ | ND | ND | ND | ND | 88199 | ND | ND | ND |
| 7:25 | 144.0571 | 1-Butanone, 4-chloro-1-(4-hydroxyphenyl)- | C_10_H_11_ClO_2_ | ND | ND | ND | ND | ND | ND | 284388 | ND |
| 7:26 | 120.0571 | Pyrazine, 2-ethenyl-6-methyl- | C_7_H_8_N_2_ | ND | ND | 916360 | ND | ND | ND | ND | ND |
| 7:27 | 132.8828 | 4-Pyrrolidin-1-ylmethyl-thiazol-2-ylamine | C_8_H_13_N_3_S | ND | ND | ND | ND | ND | ND | 162948 | ND |
| 7:27 | 120.0571 | Catecholborane | C_6_H_5_BO_2_ | ND | ND | 747387 | 1065986 | ND | ND | ND | ND |
| 7:27 | 166.8316 | N-Cyclohexylpiperidine | C1_1_H_21_N | ND | ND | ND | ND | ND | 9241 | ND | ND |
| 7:28 | 140.0661 | 1H-[1,2,3]Triazole-4-carboxylic acid, 1-(4-aminofurazan-3-yl)-5-pyrrolidin-1-ylmethyl- | C_10_H_13_N_7_O_3_ | ND | ND | ND | ND | ND | 55779 | ND | ND |
| 7:30 | 139.0629 | 1H-Pyrrole-2,5-dione, 3-ethyl-4-methyl- | C_7_H_9_NO_2_ | ND | ND | ND | 1467368 | 1036893 | ND | 1384173 | ND |
| 7:32 | 110.0603 | Ethyl tetrolate | C_6_H_8_O_2_ | ND | ND | ND | ND | ND | 1625083 | ND | ND |
| 7:32 | 151.1117 | Cyclopentene, 3-(bromomethyl)- | C_6_H_9_Br | ND | ND | 6231557 | ND | ND | ND | ND | ND |
| 7:32 | 140.0661 | Cardol monoene | C2_1_H_34_O2 | ND | ND | ND | ND | ND | 123165 | ND | ND |
| 7:33 | 138.1039 | 3-Amino-4,6-dimethylpyridone-2(1H) | C_7_H_10_N_2_O | ND | ND | ND | 36353 | ND | ND | ND | ND |
| 7:34 | 131.0154 | Levacetylmethadol | C_23_H_31_NO_2_ | ND | ND | ND | 8852 | ND | ND | ND | ND |
| 7:35 | 96.0807 | Ethylmethylpropylamine | C_6_H_15_N | ND | ND | ND | ND | 203082 | ND | ND | ND |
| 7:35 | 117.0785 | Phenethylamine, N-ethyl-à-methyl-m-(trifluoromethyl)- | C_12_H_16_F_3_N | ND | ND | ND | ND | 199027 | ND | ND | ND |
| 7:35 | 117.0784 | L-Alanine, N-formyl- | C_4_H_7_NO_3_ | ND | ND | ND | ND | ND | ND | 279746 | ND |
| 7:36 | 143.0524 | Cyclohexanone, 2-(1-methylheptyl)- | C_14_H_26_O | ND | ND | 1128803 | ND | ND | ND | ND | ND |
| 7:36 | 159.0575 | 3-Decen-5-one | C_10_H_18_O | ND | ND | 1456753 | 1353228 | ND | 2524935 | ND | ND |
| 7:36 | 139.0755 | Pyrazole, 3-(trimethylsilyl)- | C_6_H_12_N_2_Si | ND | ND | ND | 933731 | ND | ND | ND | ND |
| 7:38 | 100.0521 | 2H-Thiopyran, 3,4-dihydro- | C_5_H_8_S | ND | ND | ND | ND | ND | 1596258 | ND | ND |
| 7:38 | 154.1351 | 4-Oxononanal | C_9_H_16_O_2_ | ND | ND | ND | 3640387 | 506764 | ND | ND | ND |
| 7:38 | 134.0731 | 2,7-Octanedione, 4,4,5,5-tetramethyl- ; 4,4,5,5-Tetramethyl-2,7-octanedione | C_12_H_22_O_2_ | ND | ND | ND | 3420311 | ND | ND | ND | ND |
| 7:39 | 166.8322 | Undecane, 3-methylene- | C_12_H_24_ | 175088 | ND | ND | ND | ND | ND | ND | ND |
| 7:39 | 100.0521 | Dihydrothiapyran | C_5_H_8_S | ND | ND | 2309794 | ND | ND | ND | ND | ND |
| 7:39 | 118.9030 | Acetamide, N-tetrahydrofurfuryl-2,2,2-trichloro- | C_7_H_10_Cl_3_NO_2_ | ND | ND | ND | ND | ND | 48811 | ND | ND |
| 7:39 | 135.0129 | Hexane, 1-(hexyloxy)-4-methyl- | C_13_H_28_O | 220656 | ND | ND | ND | ND | ND | ND | ND |
| 7:42 | 160.9086 | Bis(2-ethylhexyl) methylphosphonate | C_17_H_37_O_3_P | ND | ND | 1056300 | ND | ND | ND | ND | ND |
| 7:43 | 153.0904 | 2-Furanacetaldehyde, à-propyl- | C_9_H_12_O_2_ | ND | ND | 4999619 | ND | ND | ND | ND | ND |
| 7:44 | 280.9606 | Isophytol | C_20_H_40_O | ND | ND | ND | ND | ND | 346301 | 1066025 | ND |
| 7:44 | 190.1716 | Benzene, 1,3-bis(1,1-dimethylethyl)- | C_14_H_22_ | ND | ND | ND | ND | ND | ND | ND | 225233 |
| 7:45 | 138.0677 | 4'-Fluorovalerophenone | C_11_H_13_FO | ND | ND | ND | ND | ND | ND | 322815 | ND |
| 7:50 | 139.1120 | 2-Decenal, (E)- | C_10_H_18_O | 1408124 | ND | ND | ND | 839967 | ND | 783694 | ND |
| 7:50 | 153.1273 | 2-Decenal, (Z)- | C_10_H_18_O | 1790009 | 216822 | 20251457 | 13533636 | ND | 386397 | ND | ND |
| 7:50 | 138.0507 | 2-Nonenal, (E)- | C_9_H_16_O | ND | ND | ND | ND | ND | ND | 655419 | ND |
| 7:50 | 138.0506 | 2-Decenal, (E)- ; trans-2-Decenal | C_10_H_18_O | ND | ND | ND | ND | 966006 | ND | ND | ND |
| 7:52 | 110.0562 | 3-[1-Methylethyl]-1-cyclopentene | C_8_H_14_ | ND | ND | ND | ND | 2586 | ND | ND | ND |
| 7:53 | 143.1063 | Tetrahydropyran Z-10-dodecenoate | C_17_H_30_O_3_ | ND | ND | 4179499 | ND | 1194629 | 305490 | 1636840 | ND |
| 7:54 | 154.0989 | Dihexyverine | C_20_H_35_NO_2_ | 56138 | ND | 671842 | 3072164 | ND | ND | ND | ND |
| 7:55 | 155.1023 | 3(2H)-Furanone, 5-methyl-2-octyl- | C_13_H_22_O_2_ | ND | ND | ND | ND | 391366 | ND | ND | ND |
| 7:55 | 154.0989 | 1-Oxaspiro(4,5)decan-2-one | C_9_H_14_O_2_ | ND | ND | ND | ND | 337710 | ND | ND | ND |
| 7:56 | 209.0293 | 3(2H)-Furanone, 2-hexyl-5-methyl- | C_11_H_18_O_2_ | ND | ND | 560104 | 852681 | ND | 533384 | ND | ND |
| 7:57 | 145.0234 | Trimethyl(n-pentyl)silane | C_8_H_2_0Si | ND | ND | ND | 162678 | ND | ND | ND | ND |
| 7:57 | 151.1117 | 3-Butene-1,2-diol, 1-(2-furanyl)- | C_8_H_10_O_3_ | ND | ND | ND | ND | 249793 | ND | ND | ND |
| 8:03 | 220.9222 | Ethyl 2-(methylamino)-1-phenyl-3-cyclohexene-1-carboxylate | C_16_H_21_NO_2_ | ND | ND | ND | 439750 | ND | ND | ND | ND |
| 8:03 | 131.0496 | 4,5-Dipropenyldihydro-furan-2-one | C_10_H_14_O_2_ | ND | ND | ND | ND | ND | 696100 | ND | ND |
| 8:04 | 114.9999 | Ethyl 2-cyano-3-methylbutanoate | C_8_H_13_NO_2_ | ND | ND | ND | ND | ND | 608534 | ND | ND |
| 8:04 | 86.0362 | Dimethylvinylcarbinol | C_5_H_10_O | ND | ND | 304703 | ND | ND | ND | ND | ND |
| 8:04 | 221.0837 | Dichloroacetic acid, 2-tetrahydrofurylmethyl ester | C_7_H_10_Cl_2_O_3_ | ND | ND | 309418 | ND | ND | ND | ND | ND |
| 8:07 | 198.9445 | Cyclopropane, 1-(1-methylethyl)-2-nonyl- | C_15_H_30_ | ND | ND | ND | 581951 | ND | ND | 480297 | ND |
| 8:07 | 127.0747 | Cyclopentanecarboxylic acid, 2-amino-, cis- | C_6_H_11_NO_2_ | ND | ND | ND | ND | ND | ND | 355195 | ND |
| 8:07 | 136.0634 | 2-Methylthiolane, S,S-dioxide | C_5_H_10_O_2_S | 192897 | ND | ND | ND | ND | ND | ND | ND |
| 8:07 | 139.0392 | 2-Hydroperfluoroisobutanoic acid-N,N-di(2-propenyl)amide | C_10_H_11_F_6_NO | ND | ND | ND | ND | ND | ND | 252543 | ND |
| 8:07 | 182.9498 | Propionic acid, thio-, S-undecyl ester | C_14_H_28_OS | ND | ND | 46464 | ND | ND | ND | ND | ND |
| 8:07 | 98.0475 | Succinic anhydride | C_4_H_4_O_3_ | ND | ND | 994740 | ND | ND | ND | ND | ND |
| 8:07 | 146.9389 | Cyclopentyl bromide | C_5_H_9_Br | 372956 | ND | ND | ND | ND | ND | ND | ND |
| 8:10 | 183.2064 | Spiro[bicyclo[2.2.1]heptane-2,2'-[1,3]dioxolan]-3-one, 4,7,7-trimethyl- | C_12_H_18_O_3_ | ND | ND | 150172 | ND | ND | ND | ND | ND |
| 8:11 | 125.1325 | 1,2-Octadecanediol ; 1,2-Dihydroxyoctadecane | C_18_H_38_O_2_ | ND | ND | ND | ND | 81476 | ND | ND | ND |
| 8:11 | 275.9874 | 7-Chloro-3-(4-methyl-1-piperazinyl)-4H-1,2,4-benzothiadiazine 1,1-dioxide | C_12_H_15_ClN_4_O_2_S | 73729 | ND | 314986 | ND | ND | ND | ND | ND |
| 8:11 | 144.1017 | 1-Dodecene | C_12_H_24_ | ND | ND | ND | ND | 106903 | ND | ND | ND |
| 8:12 | 139.1479 | 3,3,7,7-Tetramethyl-5-oxa-1,9-diazabicyclo[4.3.0]non-8-ene | C10H18N2O | ND | ND | ND | 259801 | ND | ND | ND | ND |
| 8:13 | 187.1570 | dl-Aspartic acid, N-acetyl-, dimethyl ester | C_8_H_13_NO_5_ | ND | ND | ND | 202943 | ND | ND | ND | ND |
| 8:13 | 145.1047 | N-Hydroxy-N-ethylcarbamic acid, 2-(methoxycarbonylamino)ethyl ester | C7H_14_N_2_O_5_ | ND | ND | ND | 197374 | ND | ND | ND | ND |
| 8:14 | 144.1019 | l-Alanyl-l-leucine, N-methoxycarbonyl-, 2,2,2-trifluoroethyl ester | C_13_H_21_F_3_N_2_O_5_ | ND | ND | ND | ND | ND | ND | 29071 | ND |
| 8:14 | 269.0489 | Sarcosylsarcosine, N-methoxycarbonyl-, propyl ester | C_11_H_20_N_2_O_5_ | ND | ND | 525954 | ND | ND | ND | ND | ND |
| 8:14 | 144.1020 | Ethanamine, 2-methoxy-N-(2-methoxyethyl)-N-methyl- | C_7_H_17_NO_2_ | ND | ND | ND | ND | ND | ND | 18889 | ND |
| 8:16 | 151.0755 | 1-Deoxy-1-[3-(3-nitrophenyl)-2-thioureido]-á-d-glucopyranose 2,3,4,6-tetraacetate | C_21_H_25_N_3_O_11_S | ND | ND | ND | ND | ND | ND | 1267036 | ND |
| 8:16 | 168.1146 | 3-Ureidopropionic acid, N-dimethylaminomethylene-, butyl ester | C_11_H_21_N_3_O_3_ | ND | ND | ND | 273379 | 55030 | ND | ND | ND |
| 8:16 | 152.1197 | 2,4-Decadienal, (E,E)- | C_10_H_1_6O | 7712614 | 1495429 | 115133269 | 19293885 | ND | 32196387 | 3052970 | ND |
| 8:17 | 152.1195 | 2'-Desoxyuridine, 3',5'-bis-O-(chloroacetyl)- | C_13_H_14_Cl_2_N_2_O_7_ | ND | ND | ND | ND | ND | ND | 1546506 | ND |
| 8:24 | 152.0830 | 8-(2-Acetyloxiran-2-yl)-6,6-dimethylocta-3,4-dien-2-one | C_14_H_20_O_3_ | ND | ND | ND | ND | ND | ND | 3020105 | ND |
| 8:26 | 104.0025 | Ethyl thiolacetate | C_4_H_8_OS | ND | ND | ND | ND | ND | 544582 | ND | ND |
| 8:26 | 104.0024 | 2-Chloro-1-buten-3-one | C_4_H_5_ClO | ND | ND | ND | ND | ND | 491441 | ND | ND |
| 8:26 | 151.0753 | Dibutylformamide | C_9_H_19_NO | ND | ND | ND | ND | ND | ND | 114934 | ND |
| 8:26 | 128.1071 | Formamide, N,N-dibutyl- | C_9_H_19_NO | ND | ND | ND | 169773 | ND | ND | 92033 | ND |
| 8:27 | 166.8210 | N,N-Dibutylformamide | C_9_H_19_NO | ND | ND | 374209 | ND | ND | ND | ND | ND |
| 8:27 | 150.0129 | Dibutylformamide | C_9_H_19_NO | ND | ND | 365493 | ND | ND | ND | ND | ND |
| 8:30 | 154.1718 | 1-Octanol, 2-butyl- | C_12_H_26_O | ND | ND | ND | ND | ND | ND | ND | 227188 |
| 8:30 | 325.9842 | Tetrazole-5-(4H)-thione, 1-benzyl-4-(4-morpholylmethyl)- | C_13_H_17_N5OS | ND | ND | 994688 | ND | ND | ND | ND | ND |
| 8:31 | 218.9184 | Heptyl methyl ethylphosphonate | C_10_H_23_O_3_P | ND | ND | 61384 | ND | 262634 | ND | ND | ND |
| 8:35 | 152.1196 | 2-n-Heptylfuran | C_11_H_18_O | ND | ND | ND | ND | 345665 | ND | ND | ND |
| 8:35 | 152.1198 | 2,4-Decadienal | C_10_H_16_O | ND | 2389442 | 94057492 | ND | ND | 67361289 | ND | ND |
| 8:37 | 138.1006 | 2,4-Nonadienal, (E,E)- | C_9_H_14_O | ND | ND | 116204140 | 7301578 | 1093117 | ND | ND | ND |
| 8:37 | 150.0678 | 2,7-Dithiaoctane | C_6_H_14_S_2_ | ND | ND | ND | 254184 | ND | ND | ND | ND |
| 8:37 | 150.0675 | Phenyl-1,2-diamine, N,4,5-trimethyl- | C_9_H_14_N_2_ | ND | ND | ND | ND | 108634 | ND | ND | ND |
| 8:37 | 151.0749 | [10]-Gingerdione | C_21_H_32_O_4_ | ND | ND | ND | ND | ND | ND | 42345 | ND |
| 8:37 | 151.0711 | Benzeneethanamine, N-[[4-hydroxyphenyl]acetyl]-4-hydroxy-3-methoxy- | C_17_H_19_NO_4_ | ND | ND | ND | ND | 113909 | ND | ND | ND |
| 8:38 | 111.0442 | 6-Azabicyclo[3,2,0]heptan-7-one | C_6_H_9_NO | ND | ND | ND | ND | ND | 9412263 | ND | ND |
| 8:38 | 152.0832 | 1,5-Diazabicyclo[4.4.0]dec-5-en-2-one | C_8_H_12_N_2_O | ND | ND | ND | 151505 | ND | ND | ND | ND |
| 8:38 | 152.0834 | Cyclohexanone, 2-methylene-5-(1-methylethyl)- | C_10_H_16_O | ND | ND | ND | 135506 | ND | ND | ND | ND |
| 8:39 | 125.0598 | 2-Pyrrolidinone, 4,4-dimethyl-5-methylidene- | C_7_H_11_NO | ND | ND | ND | ND | ND | 6638 | ND | ND |
| 8:40 | 230.9853 | 17-(N-Methylimidazolin-2-yl)heptadecanoic acid,methyl ester | C_22_H_42_N_2_O_2_ | ND | ND | ND | 792652 | ND | ND | ND | ND |
| 8:45 | 35.9762 | Hydrogen chloride | ClH | ND | ND | 5506395 | 7541169 | ND | 4633717 | 26327653 | ND |
| 8:47 | 166.0625 | Piperidine-2-carboxamide, 1-amino-N-mesityl- | C_15_H_23_N_3_O | ND | ND | ND | ND | ND | ND | 222724 | ND |
| 8:51 | 152.0469 | Benzaldehyde, 2,4-dihydroxy-6-methyl- | C_8_H_8_O_3_ | ND | ND | ND | 89595 | ND | ND | ND | ND |
| 8:57 | 139.1119 | 3,5-Difluorobenzonitrile | C_7_H_3_F_2_N | ND | ND | ND | 36799 | ND | ND | ND | ND |
| 8:58 | 139.0751 | 3-Nonen-2-one ; 3-Non-3-en-2-one | C_9_H_16_O | ND | ND | ND | ND | 1607024 | ND | ND | ND |
| 8:58 | 137.0838 | 4,4,6-Trimethyl-cyclohex-2-en-1-ol | C_9_H_16_O | 114453 | ND | 1224639 | 5735733 | 1439568 | ND | ND | ND |
| 8:58 | 155.0532 | 1,3-Dithiolo[4,5-b]furan, tetrahydro-3a-methyl- | C_6_H_10_OS_2_ | ND | ND | ND | ND | ND | ND | 676429 | ND |
| 8:58 | 149.0959 | Acetate, 4-hydroxy-3-methyl-2-butenyl- | C_7_H_12_O_3_ | ND | ND | ND | ND | ND | ND | 500731 | ND |
| 8:58 | 153.0910 | Propanamide, 3-(1-pyrrolidinyl)-N-(2-thiazolyl)- | C_10_H_15_N_3_OS | ND | ND | ND | ND | ND | 2060195 | ND | ND |
| 8:59 | 154.0502 | 2-[p-Chlorobenzyl]piperidine | C_12_H_16_ClN | ND | ND | 1368416 | ND | ND | ND | ND | ND |
| 8:59 | 193.1222 | (4-Methylsulfanylphenyl)carbamic acid, 2,6-dimethoxyphenyl ester | C_16_H_17_NO_4_S | ND | ND | ND | ND | ND | ND | 78993 | ND |
| 8:59 | 255.8908 | 2,5-Heptadien-4-ol, 3,5-dimethyl-4-[2-(pyrrolidin-1-yl)ethyl]- | C_15_H_27_NO | 81949 | ND | ND | ND | ND | ND | ND | ND |
| 9:00 | 154.0501 | Phenol, 3-methyl-4-(methylthio)- | C_8_H_10_OS | ND | ND | ND | ND | ND | ND | 56610 | ND |
| 9:01 | 166.0989 | (2-Fluorophenyl) methanol, 2-methylbutyl ether | C12H17FO | ND | ND | ND | ND | ND | 334560 | ND | ND |
| 9:01 | 110.0363 | 2-Furancarboxaldehyde, 5-methyl- | C_6_H_6_O_2_ | ND | ND | ND | 703390 | ND | ND | ND | ND |
| 9:01 | 208.1461 | 2,4,4-Trimethyl-3-(3-methylbutyl)cyclohex-2-enone | C_14_H_24_O | ND | ND | 282168 | ND | ND | ND | ND | ND |
| 9:02 | 114.0671 | sec-butyl ethyl ketone | C_7_H_14_O | ND | ND | ND | 3508623 | ND | ND | ND | ND |
| 9:03 | 154.0990 | 4,6-Dimethyloctane-3,5-dione | C_10_H_18_O_2_ | ND | ND | ND | 3597699 | ND | ND | ND | ND |
| 9:03 | 98.9994 | 1-Bromo-2-methylpropane | C_4_H_9_Br | ND | ND | ND | ND | ND | 318 | ND | ND |
| 9:05 | 128.1153 | 1-Octen-3-ol | C_8_H_16_O | ND | ND | ND | ND | ND | 10670081 | ND | ND |
| 9:10 | 173.1177 | Cyclopentanecarboxylic acid, 2-tetrahydrofurylmethyl ester | C_11_H_18_O_3_ | ND | ND | 353599 | ND | ND | ND | ND | ND |
| 9:13 | 164.0832 | 2-Dodecenal, (E)- | C_12_H_22_O | ND | ND | ND | ND | 423311 | 1691360 | ND | ND |
| 9:14 | 167.1432 | 2-Undecenal | C_11_H_20_O | ND | ND | 1533398 | 905728 | ND | 1844344 | ND | ND |
| 9:16 | 101.0597 | Silicon tetrafluoride | F_4_Si | ND | ND | ND | 574828 | ND | ND | 614831 | ND |
| 9:17 | 161.0448 | ç-Nonalactone | C_9_H_16_O_2_ | ND | ND | 455414 | ND | ND | ND | ND | ND |
| 9:17 | 177.1272 | 2-(1-Methylcyclohexyloxy)-tetrahydropyran | C_12_H_22_O_2_ | ND | ND | ND | ND | ND | ND | 421047 | ND |
| 9:17 | 101.0235 | Ethanone, 1-(3-methyloxiranyl)- | C_5_H_8_O_2_ | ND | ND | ND | ND | ND | ND | 1521662 | ND |
| 9:20 | 179.1797 | 6-Dodecanone | C1_2_H_24_O | ND | ND | 189896 | ND | ND | ND | ND | ND |
| 9:22 | 153.0909 | 1-Propylheptyl ethylphosphonofluoridate | C_12_H_26_FO_2_P | ND | ND | ND | ND | 435714 | ND | ND | ND |
| 9:22 | 125.0837 | (1-Propoxy-pentyl)-cyclopropane | C_11_H_22_O | ND | ND | ND | ND | ND | ND | 80973 | ND |
| 9:22 | 127.1483 | 5-Oxohexanethioic acid, S-t-butyl ester | C_10_H_18_O_2_S | ND | ND | ND | ND | ND | ND | 97463 | ND |
| 9:23 | 177.0547 | 26-Methyl-octacosanoic acid, pyrrolidide | C_33_H_65_NO | ND | ND | ND | 93870 | 528683 | ND | ND | ND |
| 9:23 | 113.0235 | 5-Ethylthiazole | C_5_H_7_NS | ND | ND | ND | 192415 | ND | ND | ND | ND |
| 9:23 | 111.0441 | 2,4-Heptadienal, (E,E)- | C_7_H_10_O | ND | ND | 562185 | ND | ND | ND | ND | ND |
| 9:23 | 168.1142 | cis-4,5-Epoxy-(E)-2-decenal | C_10_H_16_O_2_ | ND | ND | 1977341 | 2649322 | ND | ND | ND | ND |
| 9:24 | 122.1090 | N'-(But-2-enylidene)-2,2,3,3,4,4,5,5,6,6,7,7,7-tridecafluoroheptanohydrazide | C_11_H_7_F_13_N_2_O | ND | ND | ND | ND | ND | 2008198 | ND | ND |
| 9:27 | 128.0705 | Pentanenitrile, 3,5-dihydroxy-3-methyl | C_6_H_11_NO_2_ | ND | ND | ND | ND | ND | ND | 792272 | ND |
| 9:27 | 262.7217 | Propanoic acid, 2-methyl-, 2-ethyl-3-hydroxyhexyl ester | C_12_H_24_O_3_ | ND | ND | 583219 | ND | 98224 | ND | ND | ND |
| 9:27 | 140.0817 | Silane, (2-methoxyethyl)trimethyl- | C_6_H_16_OSi | ND | ND | 228254 | ND | ND | ND | ND | ND |
| 9:28 | 161.1328 | trans-4,5-Epoxy-(E)-2-decenal | C_10_H_16_O_2_ | ND | ND | 5956263 | 2033068 | DN | 5971155 | ND | ND |
| 9:30 | 149.0599 | Mefloquine | C_17_H_16_F_6_N2O | ND | ND | ND | ND | ND | ND | ND | ND |
| 9:31 | 128.0468 | 2-Methyl-5-(methylthio)furan | C_6_H_8_OS | ND | ND | ND | ND | ND |  | ND | ND |
| 9:33 | 129.0523 | Succinic acid, but-3-yn-2-yl 3-methylbut-2-yl ester | C_13_H_20_O_4_ | ND | ND | ND | ND | ND | 140962 | ND | ND |
| 9:34 | 197.7940 | .alfa.-Copaene | C_15_H_24_ | ND | ND | 99586 | ND | ND | ND | ND | ND |
| 9:34 | 157.0733 | 1-Ethyl-2-pyrrolidinecarboxylic acid, methyl ester | C_8_H_15_NO_2_ | ND | ND | ND | ND | 216607 | ND | 664046 | ND |
| 9:36 | 113.0598 | 3-Buten-2-one, 4-(dimethylamino)- | C_6_H_11_NO | ND | ND | ND | ND | ND | 65127 | ND | ND |
| 9:36 | 138.0550 | n-Decanol | C_10_H_22_O | ND | ND | ND | ND | ND | ND | 119136 | ND |
| 9:37 | 179.1795 | 3-Tetradecene, (Z)- | C_14_H_28_ | ND | ND | 1658558 | ND | ND | ND | ND | ND |
| 9:37 | 197.2220 | 7-Hexadecene, (Z)- | C_16_H_32_ | ND | ND | 1124160 | 263971 | ND | ND | ND | ND |
| 9:43 | 137.0594 | Dinitrodiglycol | C_4_H_8_N_2_O_7_ | ND | ND | 1466 | ND | ND | ND | ND | ND |
| 9:44 | 198.2342 | Hexadecane | C_16_H_34_ | 1992097,5 | 195503 | 42030177 | 18722407 | 3024489 | 4847631 | 573827 | ND |
| 9:45 | 131.0334 | 2-(Chloromethyl)tetrahydropyran | C_6_H_11_ClO | ND | ND | ND | 11495 | ND | ND | ND | ND |
| 9:45 | 133.1015 | 1,3-Dioxolane, 2-(2-bromoethyl)- | C_5_H_9_BrO_2_ | ND | ND | ND | ND | ND | 276211 | ND | ND |
| 9:45 | 153.0918 | 2-(2-Bromoethyl)-1,3-dioxolane ; 1,3-Dioxolane, 2-(2-bromoethyl) | C_5_H_9_BrO_2_ | ND | ND | ND | ND | ND | 268427 | ND | ND |
| 9:47 | 161.1329 | Jasmone, dihydro- | C_11_H_18_O | ND | ND | ND | 195622 | ND | 1901 | ND | ND |
| 9:47 | 183.9505 | 1-Isopropenyl-3,3-dimethyl-5-(3-methyl-1-oxo-2-butenyl)cyclopentane | C_15_H_24_O | ND | ND | ND | 420581 | ND | ND | ND | ND |
| 9:49 | 159.0473 | Cyclohexanecarboxylic acid, 4-formylphenyl ester | C_14_H_16_O_3_ | ND | ND | 584052 | 3155703 | ND | 576573 | ND | ND |
| 9:50 | 126.1038 | 4-Hexen-2-one, 3,4-dimethyl- | C_8_H_14_O | ND | ND | 504278 | ND | ND | ND | ND | ND |
| 9:50 | 166.0984 | Cyclohexanecarboxylic acid, (1H-tetrazol-5-yl)amide | C_8_H_13_N_5_O | ND | ND | ND | ND | ND | 683309 | ND | ND |
| 9:50 | 112.0759 | Butanenitrile, 4-(dimethylamino)- | C_6_H_12_N_2_ | ND | ND | ND | 58500 | ND | ND | ND | ND |
| 9:53 | 132.8830 | 1,3-Dioxolane, 2-(1-methylpropyl)- | C_7_H_14_O_2_ | ND | ND | ND | ND | ND | 264442 | ND | ND |
| 9:535 | 104.0026 | 1,3-Dioxolane, 2-sec-butyl- | C_7_H_14_O_2_ | ND | ND | ND | ND | ND | 266300 | ND | ND |
| 9:54 | 150.0673 | Glutaric acid, naphth-2-ylmethyl 2,3,4-trifluorophenyl ester | C_22_H_17_F_3_O_4_ | ND | ND | ND | ND | ND | ND | 4429 | ND |
| 9:55 | 153.0503 | Pyrazole-3-carboxylic acid, 4,5-dihydro-5,5-di-t-butyl-, ethyl ester | C_14_H_26_N_2_O_2_ | ND | ND | ND | ND | ND | ND | 5508 | ND |
| 9:55 | 165.1635 | 1-Pyridineacetic acid, 4-(aminocarbonyl)hexahydro-, decyl ester | C_18_H_34_N_2_O_3_ | ND | ND | ND | 55074 | ND | ND | 31864 | ND |
| 9:56 | 152.0469 | Vanillin | C_8_H_8_O_3_ | ND | ND | ND | ND | 150982 | ND | ND | ND |
| 9:56 | 152.0467 | 1H-Indene, 4,7-difluoro- | C_9_H_6_F_2_ | ND | ND | ND | 67336 | ND | ND | ND | ND |
| 9:57 | 167.1073 | 2-Hydroxy-4-methoxybenzaldehyde, acetate | C_10_H_10_O_4_ | ND | ND | ND | ND | ND | ND | 71913 | ND |
| 9:57 | 151.0391 | Pyrolo[3,2-d]pyrimidin-2,4(1H,3H)-dione | C_6_H_5_N_3_O_2_ | ND | ND | ND | ND | 111386 | ND | ND | ND |
| 9:57 | 152.0471 | Benzaldehyde, 3-hydroxy-4-methoxy- | C_8_H_8_O_3_ | ND | ND | 152933 | ND | 45640 | ND | ND | ND |
| 10:02 | 151.1115 | iso-Propyl levulinate | C_8_H_14_O_3_ | ND | ND | ND | ND | ND | 856708 | ND | ND |
| 10:02 | 138.1039 | 2,3-Octanedione | C_8_H_14_O_2_ | ND | ND | ND | ND | ND | 880579 | ND | ND |
| 10:06 | 175.1356 | Carbonic acid, ethyl 3-(1-methylethoxy)phenyl ester | C_12_H_16_O_4_ | ND | ND | ND | ND | ND | ND | 64522 | ND |
| 10:07 | 127.0394 | Fumaric acid, ethyl 2,3,5-trichlorophenyl ester | C_12_H_9_Cl_3_O_4_ | 44584 | 45299 | 946930 | 367286 | 189385 | 72201 | 189793 | ND |
| 10:07 | 167.1064 | 1,1-Cyclopropanedicarboxylic acid, 2,6-bis(1,1-dimethylethyl)-4-methylphenyl methyl ester | C_21_H_30_O_4_ | ND | ND | ND | ND | ND | ND | 236798 | ND |
| 10:07 | 139.1118 | à-Bromo-2,4-difluorotoluene | C_7_H_5_BrF_2_ | ND | ND | ND | ND | ND | 64561 | ND | ND |
| 10:09 | 195.1334 | 6-Bromomethyl-5-methyl-bicyclo[3.1.0]hexan-2-one | C_8_H_11_BrO | ND | ND | 1257530 | ND | ND | 1072825 | ND | ND |
| 10:09 | 153.1266 | Imidazole-2-hydrazide-1-carboxylic acid, methyl ester | C_5_H_6_N_4_O_2_ | ND | ND | 1155406 | ND | ND | ND | ND | ND |
| 10:18 | 131.0679 | 5-Decyne | C_10_H_18_ | ND | ND | 426001 | ND | ND | ND | ND | ND |
| 10:18 | 119.9109 | 4,5-Nonadiene | C_9_H_16_ | ND | ND | 353820 | ND | ND | ND | ND | ND |
| 10:22 | 158.1302 | Nonanoic acid, 9-oxo-, methyl ester | C_10_H_18_O_3_ | ND | ND | ND | 246734 | 93835 | 70768 | 191379 | ND |
| 10:22 | 155.1067 | Azelaaldehydic acid, methyl ester | C_10_H_18_O_3_ | ND | ND | ND | ND | ND | ND | 151163 | ND |
| 10:23 | 199.7916 | Azelaaldehydic acid, methyl ester | C_10_H_18_O_3_ | ND | ND | ND | ND | 113265 | ND | ND | ND |
| 10:26 | 151.1485 | 1,7-Nonadiene, 4,8-dimethyl- | C_11_H_20_ | 149838 | ND | ND | ND | ND | ND | ND | ND |
| 10:26 | 151.1483 | Cyclooctyl bromide | C_8_H_15_Br | 145686 | ND | ND | ND | ND | ND | ND | ND |
| 10:26 | 138.1400 | 2,6-Octadiene, 4,5-dimethyl- | C_10_H_18_ | ND | ND | 899068 | ND | ND | ND | ND | ND |
| 10:26 | 179.1795 | Cyclooctane, 1-(diethylboryl)- | C_12_H_25_B | ND | ND | 769230 | ND | ND | ND | ND | ND |
| 10:27 | 152.1161 | Dibutyl propanephosphonate | C_11_H_25_O_3_P | ND | ND | ND | ND | ND | 67292 | ND | ND |
| 10:29 | 112.0759 | Methyl 1-acetylpyrrolidine-2-carboxylate | C_8_H_13_NO_3_ | ND | ND | ND | ND | 141389 | ND | 626906 | ND |
| 10:30 | 131.0852 | 1-(2-Hydroxymethylpyrrolidin-1-yl)ethanone | C_7_H_13_NO_2_ | ND | ND | ND | ND | 142961 | ND | ND | ND |
| 10:33 | 198.0805 | 2,6-Difluoro-à-methylbenzyl alcohol, n-pentyl ether | C_13_H_18_F_2_O | ND | ND | ND | ND | ND | 37109 | ND | ND |
| 10:34 | 208.0332 | L-Proline, 1-(chlorocarbonyl)-, methyl ester | C_7_H_10_ClNO_3_ | ND | ND | ND | ND | ND | 350878 | ND | ND |
| 10:35 | 194.2035 | Ether, 1-dodecenyl methyl | C_13_H_26_O | ND | ND | 340863 | ND | ND | ND | ND | ND |
| 10:38 | 168.0780 | Dimethyl,fluoromethyl,phenylsilane | C_9_H_13_FSi | ND | ND | ND | ND | 91342 | ND | ND | ND |
| 10:39 | 168.0783 | Fumaric acid, dicyclobutyl ester | C_12_H_16_O_4_ | ND | ND | ND | ND | 144561 | ND | ND | ND |
| 10:40 | 194.1305 | O-2-Methylpentyl, methylphosphonic acid, trimethylsilyl ester | C1_0_H_25_O_3_PSi | ND | ND | ND | 95970 | ND | ND | ND | ND |
| 10:40 | 168.0782 | 2,4,5-Trihydroxyphenyl-p-chlorobenzylketone | C_14_H1_1_ClO_4_ | ND | ND | ND | 86056 | ND | ND | ND | ND |
| 10:40 | 255.0143 | Homocapsaicin | C_19_H_29_NO_3_ | ND | ND | 208701 | ND | ND | ND | ND | ND |
| 10:40 | 195.7960 | Pyridine-3-carbonitrile, 2-[2-(3,4-dihydroxyphenyl)-2-oxoethylthio]-4-methoxymethyl-6-methyl- | C_17_H_16_N2O_4_S | ND | ND | ND | ND | ND | 212076 | ND | ND |
| 10:40 | 195.1339 | (E)-N-(4-Hydroxy-3-methoxybenzyl)-9-methyldec-7-enamide | C_19_H_29_NO_3_ | ND | ND | ND | ND | ND | 229853 | ND | ND |
| 10:40 | 195.1334 | Capsaicin | C_18_H_27_NO_3_ | ND | ND | ND | ND | ND | ND | ND | ND |
| 10:41 | 162.0675 | Benzo[b]thiophene, 3,5-dimethyl- | C_10_H_10_S | ND | ND | ND | 950257 | ND | ND | ND | ND |
| 10:44 | 154.1716 | Cyclopentaneacetic acid, 2-(hydroxymethyl)-3-methyl-, ë-lactone | C_9_H_14_O_2_ | ND | ND | 653203 | ND | ND | ND | ND | ND |
| 10:44 | 136.1244 | Dibutylacetylene | C_10_H_18_ | ND | ND | 559944 | ND | ND | ND | ND | ND |
| 10:45 | 199.7916 | Carbonic acid, dodecyl vinyl ester | C_15_H_28_O_3_ | ND | ND | ND | ND | 276527 | ND | ND | ND |
| 10:45 | 287.8786 | Octadecane, 5,14-dibutyl- | C_26_H_54_ | ND | ND | ND | ND | 198714 | ND | ND | ND |
| 10:45 | 141.0910 | 2,2-Dimethyl-3-heptanone | C_9_H_18_O | ND | ND | ND | ND | ND | ND | 451095 | ND |
| 10:46 | 113.0961 | Neomenthylamine | C_10_H_21_N | ND | ND | ND | ND | ND | 309285 | ND | ND |
| 10:46 | 113.1326 | L-Proline, N-(cyclohexanecarbonyl)-, butyl ester | C_16_H_27_NO_3_ | ND | ND | ND | ND | ND | ND | ND | ND |
| 10:47 | 169.1941 | Decane, 3,3,6-trimethyl- | C_13_H_28_ | ND | ND | ND | 629124 | ND | 265350 | ND | ND |
| 10:47 | 169.1948 | Tridecane, 4-methyl- | C_14_H_30_ | ND | ND | 1063654 | ND | ND | ND | ND | ND |
| 10:48 | 161.1323 | Pregna-5,9(11)-dien-20-ol-3-one ethylene ketal | C_23_H_34_O_3_ | ND | ND | 162427 | ND | ND | ND | ND | ND |
| 10:48 | 171.1386 | Glutaric acid, 3-hexyl propyl ester | C_14_H_26_O_4_ | ND | ND | ND | ND | ND | 190620 | ND | ND |
| 10:50 | 158.1257 | 1-Ethyl-1-isopropoxy-1-silacyclohexane | C1_0_H_22_OSi | ND | ND | ND | ND | ND | 176744 | ND | ND |
| 10:50 | 163.0393 | Benzeneacetamide, N,N-dimethyl- | C_10_H1_3_NO | ND | ND | ND | 64158 | ND | ND | ND | ND |
| 10:51 | 141.1640 | Nonadecane | C_19_H_4_0 | 309475 | ND | ND | 824887 | ND | ND | ND | ND |
| 10:52 | 194.0573 | Sulfurous acid, butyl dodecyl ester | C_16_H_34_O_3_S | ND | ND | 424892 | ND | ND | ND | ND | ND |
| 10:53 | 137.0961 | Ethyl 2-(2-chloroacetamido)-3,3,3-trifluoro-2-(4-fluorobenzylamino)propionate | C_14_H_15_ClF_4_N_2_O_3_ | ND | ND | ND | ND | ND | 78977 | ND | ND |
| 10:56 | 150.1040 | 3-Undecen-5-yne, (E)- | C_11_H_18_ | ND | ND | 811996 | ND | ND | ND | ND | ND |
| 10:56 | 152.1162 | Hexanoic acid, tridec-2-ynyl ester | C_19_H_34_O_2_ | ND | ND | ND | ND | ND | ND | ND | ND |
| 10:57 | 150.1037 | Xanthinin | C_17_H_22_O_5_ | ND | ND | ND | ND | ND | 629705 | ND | ND |
| 10:59 | 147.1170 | Undecanal, 2-methyl- | C_12_H_24_O | ND | ND | ND | ND | 283883 | ND | ND | ND |
| 10:59 | 127.1482 | Cyclobutanol, 1-butyl- | C_8_H_16_O | ND | ND | 315991 | ND | ND | ND | ND | ND |
| 10:59 | 140.1561 | Dimethylaminoethylbenzene | C_10_H_15_N | ND | ND | ND | 238929 | ND | ND | ND | ND |
| 10:59 | 195.7961 | Pyrido[2,3-d]pyrimidin-4(3H)-one, 3-(3-dimethylaninopropyl)- | C_12_H_16_N_4_O | ND | ND | ND | 340639 | ND | ND | 189747 | ND |
| 10:59 | 176.1194 | 4-Heptanol, 1-dimethylamino-4,6-dimethyl- | C_11_H_25_NO | ND | ND | 218616 | ND | ND | ND | ND | ND |
| 11:01 | 190.1713 | Benzene, octyl- | C_14_H_22_ | ND | ND | ND | ND | ND | 16287 | ND | ND |
| 11:06 | 285.8764 | 2,4-Epoxymenthanophenanthren-12-one, 1,2,3,4,4a,4b,5,6,7,8,10,10a-dodecahydro-1-hydroxy-1-(2-ethoxyvinyl-2-propyl-4b-methyl-7,7-(2,5-dioxocyclopentyl)- | C_25_H_36_O_6_ | ND | ND | 81301 | ND | ND | ND | ND | ND |
| 11:06 | 375.9795 | Captafol | C_10_H_9_Cl_4_NO_2_S | ND | ND | 695660 | ND | ND | ND | ND | ND |
| 11:08 | 221.1537 | 2,5-cyclohexadien-1-one, 2,6-bis(1,1-dimethylethyl)-4-hydroxy-4-methyl- | C_15_H_24_O_2_ | ND | ND | ND | 136339 | ND | ND | ND | ND |
| 11:08 | 180.1147 | Phenol, 3-tert-butyl-4-methoxy- | C_11_H_16_O_2_ | ND | ND | ND | 51668 | ND | ND | ND | ND |
| 11:08 | 221.1535 | Cyclopentanone, 2-acetyl-3,3-dimethyl-2-(3-oxo-1-butenyl)-, (E)- | C_13_H_18_O_3_ | ND | ND | 79387 | ND | ND | ND | ND | ND |
| 11:08 | 221.1540 | 2,5-cyclohexadien-1-one, 2,6-bis(1,1-dimethylethyl)-4-hydroxy-4-methyl- | C_15_H_24_O_2_ | ND | ND | 78939 | ND | ND | ND | ND | ND |
| 11:10 | 157.0782 | Phenyl trans-2-phenyl-1-cyclopropane sulfonate | C_15_H_14_O_3_S | ND | ND | ND | ND | ND | 123208 | ND | ND |
| 11:14 | 264.7194 | Heptane, 1,1,1,3-tetrachloro- | C_7_H_12_Cl_4_ | ND | ND | ND | ND | ND | 146862 | ND | ND |
| 11:18 | 182.2030 | 4-Tridecene, (Z)- | C_13_H_26_ | ND | ND | ND | 421491 | ND | ND | ND | ND |
| 11:18 | 193.1222 | Cyclobutane, 1,1-dimethyl-2-octyl- | C_14_H_28_ | ND | ND | ND | ND | ND | ND | 229344 | ND |
| 11:18 | 138.0915 | 1-(3-Methoxy-5-methylphenyl)-N-methylpropan-2-amine | C_12_H_19_NO | ND | ND | ND | ND | 232851 | ND | ND | ND |
| 11:18 | 234.9134 | 3-[(2-Dimethylamino-ethylamino)-methyl]-8a-methyl-5-methylene-decahydro-naphtho[2,3-b]furan-2-one | C_19_H_32_N_2_O_2_ | ND | ND | ND | 125230 | ND | ND | ND | ND |
| 11:18 | 154.1717 | Isothujol | C_10_H_18_O | ND | ND | 1092596 | ND | ND | ND | ND | ND |
| 11:18 | 210.2342 | 3-Tetradecyn-1-ol | C_14_H_26_O | ND | ND | 753482 | ND | ND | ND | ND | ND |
| 11:18 | 166.0624 | N-[3-[N-Aziridyl]propylidene]-3-dimethylaminopropylamine | C_10_H_21_N_3_ | ND | ND | ND | ND | ND | ND | 270751 | ND |
| 11:19 | 139.0753 | 2H-Pyran-2-one, 5,6-dihydro-6-pentyl- | C_10_H_16_O_2_ | ND | ND | ND | ND | ND | ND | ND | 579656 |
| 11:19 | 139.0756 | 2H-Pyran-2-one, 5,6-dihydro-6-propyl- | C_8_H_12_O_2_ | ND | ND | ND | ND | ND | ND | ND | 703515 |
| 11:19 | 150.1039 | (1S,3S,4S,5R)-1-Isopropyl-4-methylbicyclo[3.1.0]hexan-3-ol ; Bicyclo[3.1.0]hexan-3-ol, 4-methyl-1-(1-methylethyl)-, (1S,3S,4S,5R)- | C_10_H_18_O | ND | ND | ND | ND | ND | 254333 | ND | ND |
| 11:19 | 150.1039 | 3-Thujanol, (1S,3S,4S,5R)-(+)- | C_10_H_18_O | ND | ND | ND | ND | ND | 290055 | ND | ND |
| 11:24 | 285.8762 | N-Carboxy-1-[4-chlorophenyl]-2-[4-piperidyl]-1,2-epoxypropane | C_15_H_18_ClNO_3_ | ND | ND | ND | ND | ND | 51302 | ND | ND |
| 11:25 | 127.0390 | 1H-Imidazole, 1-methyl-5-nitro- | C_4_H_5_N_3_O_2_ | ND | ND | ND | ND | 37738 | ND | ND | ND |
| 11:25 | 127.0391 | Phosphoric acid, dimethyl pentyl ester | C_7_H_17_O_4_P | ND | ND | ND | ND | ND | ND | ND | ND |
| 11:27 | 171.0890 | l-Proline, N-ethoxycarbonyl-, butyl ester | C_12_H_21_NO_4_ | ND | ND | ND | ND | 93902 | ND | ND | ND |
| 11:27 | 177.1273 | d-Proline, N-ethoxycarbonyl-, ethyl ester | C_10_H_17_NO_4_ | ND | ND | ND | ND | ND | ND | 527224 | ND |
| 11:29 | 415.0367 | 3-Isopropoxy-1,1,1,7,7,7-hexamethyl-3,5,5-tris(trimethylsiloxy)tetrasiloxane | C_18_H_52_O_7_Si_7_ | 81934 | 974164 | 177148 | 126823 | 125636 | 93002 | ND | 102176 |
| 11:30 | 415.0358 | 3-Butoxy-1,1,1,7,7,7-hexamethyl-3,5,5-tris(trimethylsiloxy)tetrasiloxane | C_19_H_54_O_7_Si_7_ | ND | ND | 159243 | ND | ND | ND | ND | 93628 |
| 11:31 | 139.0754 | cis-8-Isopropylbicyclo[4.3.0]non-3-ene | C_12_H_20_ | ND | ND | 45755 | ND | ND | ND | ND | ND |
| 11:32 | 150.1039 | Undecane | C_11_H_24_ | ND | ND | ND | ND | ND | 448130 | ND | ND |
| 11:34 | 328.0361 | 1,1,2,2(H)-Heptadecafluorodecyl acrylate | C_13_H_7_F1_7_O_2_ | ND | ND | 55438 | ND | ND | ND | ND | ND |
| 11:34 | 166.1717 | Cyclopropanecarboxylic acid, oct-3-en-2-yl ester | C_12_H_20_O_2_ | 871372,5 | 320689 | 5366708 | ND | ND | 300814 | N D | ND |
| 11:34 | 287.8796 | 2-Propenoic acid, 2-methyl-, 2-ethyl-2-[[(2-methyl-1-oxo-2-propenyl)oxy]methyl]-1,3-propanediyl ester | C_18_H_26_O_6_ | ND | ND | ND | ND | ND | 292621 | ND | ND |
| 11:37 | 99.0442 | 2H-Pyran-2-one, tetrahydro-6-propyl- | C_8_H_14_O_2_ | ND | ND | ND | ND | ND | ND | ND | 36305 |
| 11:38 | 181.1219 | 2,5-Difluorobenzoic acid, 4-chloro-2-methylphenyl ester | C_14_H_9_ClF_2_O_2_ | ND | 293668 | ND | ND | ND | ND | ND | ND |
| 11:39 | 181.1231 | 2,5-Difluorobenzoic acid, 2-formyl-4,6-dichlorophenyl ester | C_14_H_6_Cl_2_F_2_O_3_ | 231424 | ND | ND | 1291470 | ND | ND | ND | ND |
| 11:39 | 182.1260 | 2,4-Difluorobenzoic acid, 2-formyl-4,6-dichlorophenyl ester | C_14_H_6_Cl_2_F_2_O_3_ | ND | ND | 2462639 | 1275128 | 676970 | 398202 | ND | ND |
| 11:39 | 181.1224 | 2,6-Difluorobenzoic acid, 4-chlorophenyl ester | C_13_H_7_ClF_2_O_2_ | ND | ND | ND | ND | ND | 352880 | ND | ND |
| 11:40 | 208.1093 | Benzamide, 2,6-difluoro-N-heptyl- | C_14_H_19_F_2_NO | ND | ND | ND | ND | ND | ND | 2915148 | ND |
| 11:40 | 204.1870 | à-Cubebene | C_15_H_24_ | 38228 | ND | ND | ND | ND | ND | ND | ND |
| 11:40 | 161.1329 | 2-Chlorobenzoic acid, 3-methylbutyl-2 ester | C_12_H1_5_ClO_2_ | ND | ND | ND | ND | 34648 | ND | ND | ND |
| 11:40 | 163.0386 | Ethyl hexadecanoate | C_18_H_36_O_2_ | ND | ND | ND | 29760 | ND | ND | ND | ND |
| 11:48 | 197.0782 | 2,2-Dimethylpropanoic acid, tridec-2-ynyl ester | C_18_H_32_O_2_ | ND | ND | ND | 166666 | ND | ND | ND | ND |
| 11:48 | 165.1276 | 2-Propanone, 1,1,3,3-tetrachloro- | C_3_H_2_Cl_4_O | ND | ND | ND | ND | ND | 200601 | ND | ND |
| 11:48 | 165.1272 | Valeric acid, tridec-2-ynyl ester | C1_8_H_32_O_2_ | ND | ND | ND | ND | ND | 284201 | ND | ND |
| 11:49 | 206.1659 | Phenol, 2,5-bis(1,1-dimethylethyl)- | C_14_H_22_O | ND | ND | 129599 | ND | ND | ND | ND | ND |
| 11:49 | 206.1661 | 2,4-Di-tert-butylphenol | C_14_H_22_O | ND | ND | 130559 | ND | ND | ND | ND | ND |
| 11:49 | 100.0521 | Propanal, dimethylhydrazone | C_5_H_12_N_2_ | ND | ND | ND | ND | 62168 | 362642 | ND | ND |
| 11:50 | 100.0520 | 2-Piperazinone | C_4_H_8_N_2_O | ND | ND | ND | ND | ND | ND | ND | ND |
| 11:55 | 122.1087 | 1,2-Ethanediamine, N'-ethyl-N,N-dimethyl- | C_6_H_16_N_2_ | ND | ND | ND | ND | 152927 | ND | 291417 | ND |
| 11:55 | 140.1194 | 2,5-Diethylthiophene | C_8_H_12_S | ND | ND | ND | 131821 | ND | ND | ND | ND |
| 11:56 | 140.1197 | N,N'-Dipropylidene-1,1-diaminopropane | C_9_H_18_N_2_ | ND | ND | 165774 | ND | ND | ND | ND | ND |
| 11:56 | 164.0469 | Nickel, [ethylenebis(dimethylamine)][(E)-á-phenylsulfonylmethylacrylate] | C_16_H_26_N_2_NiO_4_S | ND | ND | ND | 176676 | ND | ND | ND | ND |
| 11:56 | 173.0962 | Carbonic acid, 6-chlorohexyl 2-methoxyethyl ester | C_10_H_19_ClO_4_ | ND | ND | ND | ND | ND | ND | 307848 | ND |
| 11:57 | 185.1537 | Dodecanoic acid, methyl ester | C_13_H_26_O_2_ | ND | ND | ND | 129213 | ND | ND | ND | ND |
| 11:58 | 185.1525 | Undecanoic acid, methyl ester | C_12_H_24_O_2_ | ND | ND | 157762 | 109585 | ND | ND | 183710 | ND |
| 11:59 | 99.0440 | 2-Buten-1-ol, propanoate | C_7_H_12_O_2_ | ND | ND | ND | ND | ND | 63392 | ND | ND |
| 12:00 | 155.0856 | 2-Thiophenecarboxamide, N-heptyl-N-(2-thiophenecarbonyl)- | C_17_H_21_NO_2_S_2_ | ND | ND | 56362 | ND | ND | ND | ND | ND |
| 12:02 | 204.1872 | á-copaene | C_15_H_24_ | 159706 | ND | ND | ND | ND | ND | ND | ND |
| 12:02 | 207.1749 | (3R,3aR,3bR,4S,7R,7aR)-4-Isopropyl-3,7-dimethyloctahydro-1H-cyclopenta[1,3]cyclopropa[1,2]benzen-3-ol | C_15_H_26_O | 138466 | ND | ND | ND | ND | ND | ND | ND |
| 12:02 | 204.1867 | Bicyclo[4.4.0]dec-1-ene, 2-isopropyl-5-methyl-9-methylene- | C_15_H_24_ | ND | ND | 69280 | ND | ND | ND | ND | ND |
| 12:02 | 207.1740 | (3R,3aR,3bR,4S,7R,7aR)-4-Isopropyl-3,7-dimethyloctahydro-1H-cyclopenta[1,3]cyclopropa[1,2]benzen-3-ol | C_15_H_26_O | ND | ND | ND | 96666 | ND | ND | ND | ND |
| 12:05 | 194.0946 | Benzoic acid, 4-ethoxy-, ethyl ester | C_11_H_14_O_3_ | 45688 | ND | 61373 | ND | ND | ND | ND | ND |
| 12:05 | 194.0945 | Propan-2-ol, 1-(2-isopropyl-5-methylcyclohexyloxy)-3-(4-morpholyl)- | C_17_H_33_NO_3_ | ND | ND | ND | ND | ND | 447330 | ND | ND |
| 12:07 | 181.1179 | 2-Butyl-6-methyl-4-pyrimidinol | C_9_H_14_N_2_O | ND | ND | 109605 | ND | ND | ND | 403840 | ND |
| 12:07 | 180.1146 | 2,3-Bis(1-methylallyl)pyrrolidine | C_12_H_21_N | ND | ND | ND | 174007 | ND | ND | ND | ND |
| 12:07 | 180.1144 | Olivetol | C_11_H_16_O_2_ | ND | ND | 123037 | 182348 | ND | ND | ND | ND |
| 12:07 | 180.1148 | Olivetol | C_11_H_16_O_2_ | ND | ND | ND | ND | ND | ND | 332756 | ND |
| 12:08 | 178.0625 | Toluene, 4-(1,1-dimethyl-2-propynyloxy)- | C_12_H_14_O | ND | ND | ND | 51633 | DN | ND | ND | ND |
| 12:09 | 178.0625 | 2,3,4-Trifluorobenzoic acid, 2-methylbutyl ester | C_12_H_13_F_3_O_2_ | ND | ND | ND | 39188 | ND | ND | ND | ND |
| 12:12 | 167.0518 | N-Isopropyl-1-piperazineacetamide | C_9_H_19_N_3_O | ND | ND | ND | 43041 | ND | ND | ND | ND |
| 12:12 | 117.0183 | 5à-Androstan-3-one, 17á-hydroxy-4à-methyl-, cyclic ethylene acetal | C_22_H_36_O_3_ | ND | ND | 81804 | 41496 | ND | ND | ND | ND |
| 12:12 | 375.9797 | Cholestan-3-one, 4-methyl-, cyclic 1,2-ethanediyl acetal, (4à,5à)- | C_30_H_52_O_2_ | ND | ND | 92174 | ND | ND | ND | ND | ND |
| 12:14 | 144.0410 | 4-Fluoro-2-methoxyphenol, pentyl ether | C_12_H_17_FO_2_ | ND | ND | ND | ND | ND | 77744 | ND | ND |
| 12:15 | 142.0446 | 1-Aminocyclopentanecarboxylic acid, N-methoxycarbonyl-, methyl ester | C_9_H_15_NO_4_ | ND | ND | ND | ND | ND | 81664 | ND | ND |
| 12:17 | 222.0388 | 1-Chloromethyl-1-isopentyloxy-1-silacyclohexane | C_11_H_23_ClOSi | ND | ND | 357883 | ND | ND | 847247 | ND | ND |
| 12:18 | 197.0786 | 1-Ethyl-1-hexyloxy-1-silacyclopentane | C_12_H_26_OSi | ND | ND | ND | 48111 | ND | ND | ND | ND |
| 12:19 | 180.1146 | 2(4H)-Benzofuranone, 5,6,7,7a-tetrahydro-4,4,7a-trimethyl-, (R)- | C_11_H_16_O_2_ | 318105 | ND | ND | ND | 406161 | ND | 1083958 | ND |
| 12:19 | 180.1146 | 2(4H)-Benzofuranone, 5,6,7,7a-tetrahydro-4,4,7a-trimethyl- | C_11_H_16_O_2_ | ND | ND | ND | ND | 476445 | ND | 1180997 | ND |
| 12:20 | 211.2421 | 2-Hexyl-1-octanol | C_14_H_30_O | ND | ND | ND | 1745665 | ND | ND | ND | ND |
| 12:20 | 221.1901 | Pentadecane, 7-methyl- | C_16_H_34_ | 168230,5 | ND | 3330412 | 1609402 | 210654 | ND | ND | ND |
| 12:20 | 177.0341 | Quinoline, 2-chloro-4-methyl- | C_10_H_8_ClN | 142843 | 44652 | ND | ND | ND | ND | ND | ND |
| 12:22 | 167.0852 | 2,2-Dimethylpropanoic acid, 3-methylbut-2-enyl ester | C_10_H1_8_O_2_ | ND | ND | 270704 | ND | ND | ND | ND | ND |
| 12:25 | 287.8778 | Eicosane, 2,4-dimethyl- | C_22_H_46_ | ND | ND | ND | 345360 | ND | ND | ND | ND |
| 12:25 | 169.1943 | Heptane, 2,2,3,3,5,6,6-heptamethyl- | C_14_H_30_ | ND | ND | 833788 | ND | ND | ND | ND | ND |
| 12:25 | 170.1988 | Tetradecane, 4-ethyl- | C_16_H_34_ | ND | ND | 783132 |  | ND | ND | ND | ND |
| 12:29 | 152.1191 | Cyclohexanamine, N-(2,3-dichloro-2-propen-1-ylidene)-, N-oxide | C_9_H_13_Cl_2_NO | ND | ND | ND | ND | ND | 513223 | ND | ND |
| 12:29 | 142.0776 | 2-Butyl-3,4,5,6-tetrahydropyridine | C_9_H_17_N | ND | ND | ND | ND | ND | ND | 30269 | ND |
| 12:33 | 131.0499 | Fumaric acid, decyl 2-phenylethyl ester | C_22_H_32_O_4_ | ND | ND | ND | ND | ND | 337795 | ND | ND |
| 12:33 | 115.1116 | Cyclohexanecarboxylic acid, 2-phenylethyl ester | C1_5_H_20_O_2_ | ND | ND | ND | ND | ND | 398311 | ND | ND |
| 12:33 | 208.1088 | 2,3-Butanediol, 2,3-bis(5-methyl-2-furanyl)- | C_14_H_18_O_4_ | ND | ND | ND | ND | ND | ND | 304364 | ND |
| 12:33 | 125.0598 | 4N-Methylcytosine | C_5_H_7_N_3_O | ND | ND | ND | ND | ND | ND | 133968 | ND |
| 12:33 | 169.1224 | Ethyl 4-(ethyloxy)-2-oxobut-3-enoate | C_8_H_12_O_4_ | ND | ND | 263212 | ND | ND | ND | ND | ND |
| 12:38 | 250.9072 | Benzoic acid, 2-hydroxy-4-[(2-hydroxy-4-methoxy-6-methylbenzoyl)oxy]-6-methyl-, methyl ester | C_18_H_18_O_7_ | ND | ND | ND | 45194 | ND | ND | ND | ND |
| 12:39 | 165.0785 | 6-Methoxy-2-(methylamino)tropone | C_9_H_11_NO_2_ | ND | ND | ND | ND | ND | ND | 72042 | ND |
| 12:40 | 329.0321 | 4-Methylimidazole-5-butyric acid, 2,2,2-trifluoroethyl(ester) | C_10_H_13_F_3_N_2_O_2_ | ND | ND | ND | 44443 | ND | ND | ND | ND |
| 12:41 | 134.0729 | 3-Methylbut-2-enoic acid, 2-methylpentyl ester | C_11_H_20_O_2_ | ND | ND | ND | ND | ND | 212044 | ND | ND |
| 12:41 | 176.0462 | Dimethylamine (N-B)bis(trifluoromethyl)(cyano)borane | C_5_H_7_BF_6_N_2_ | ND | ND | ND | 25404 | ND | ND | ND | ND |
| 12:41 | 123.0441 | 6-Undecanol | C_11_H_24_O | ND | ND | ND | ND | ND | 204147 | ND | ND |
| 12:41 | 136.0153 | 1,6-Hexanediamine, N,N'-dimethyl- | C_8_H_20_N_2_ | ND | ND | ND | 67231 | ND | ND | ND | ND |
| 12:42 | 169.1585 | (Octadecyl E)-2-methylbut-2-enoate | C_23_H_44_O_2_ | ND | ND | 57866 | ND | ND | ND | ND | ND |
| 12:43 | 141.1644 | Decane, 3,8-dimethyl- | C_12_H_26_ | 88546 | ND | ND | 449120 | 603360 | ND | ND | ND |
| 12:43 | 197.2261 | Octadecane, 2-methyl- | C_19_H_40_ | ND | ND | ND | 726815 | ND | ND | ND | ND |
| 12:43 | 196.2189 | Tetracontane, 3,5,24-trimethyl- | C_43_H_88_ | ND | ND | ND | ND | 156801 | ND | ND | ND |
| 12:43 | 328.0363 | Sulfurous acid, octadecyl pentyl ester | C_23_H_48_O_3_S | ND | ND | ND | ND | 158826 | ND | ND | ND |
| 12:44 | 153.1275 | 3-Heptene, 2,2,3,5,5,6,6-heptamethyl- | C_14_H_28_ | ND | ND | ND | ND | ND | 98204 | ND | ND |
| 12:45 | 180.0782 | Ether, 5-bromopentyl methyl | C_6_H_13_BrO | ND | ND | ND | ND | ND | ND | 88692 | ND |
| 12:46 | 165.0548 | 6,11-Dimethyl-2,6,10-dodecatrien-1-ol | C_14_H_24_O | ND | 351614 | ND | ND | ND | ND | 537392 | ND |
| 12:47 | 139.1440 | Cyclooctanemethanol | C_9_H_18_O | 1701609 | ND | ND | ND | ND | ND | ND | ND |
| 12:47 | 208.1458 | Acetophenone, 2'-(trimethylsiloxy)- | C_11_H_16_O_2_Si | ND | ND | ND | ND | ND | ND | 243881 | ND |
| 12:47 | 204.1873 | Phenylacetic acid, 3-methylbut-2-enyl ester | C_13_H_16_O_2_ | ND | ND | ND | ND | ND | 317075 | ND | ND |
| 12:47 | 165.1641 | Cyclopropanecarboxylic acid, undec-2-enyl ester | C_15_H_26_O_2_ | 1664472 | 302939 | 11585172 | ND | ND | 300814 | N D | ND |
| 12:51 | 155.0852 | Glutaric acid, isobutyl 2-pentyl ester | C_14_H_26_O_4_ | ND | ND | ND | 49814 | ND | ND | ND | ND |
| 12:51 | 170.1090 | Butylphosphonic acid, di(5-methoxy-3-methylpentyl) ester | C_18_H_39_O_5_P | ND | ND | ND | 55023 | ND | ND | ND | ND |
| 12:52 | 180.1143 | 3-Hexanoyltetrahydrofuran-2,4-dione | C_10_H_14_O_4_ | ND | ND | ND | ND | 69619 | ND | ND | ND |
| 12:52 | 180.1144 | Diglycolic acid, 4-methoxyphenyl nonyl ester | C_20_H_30_O_6_ | 45650 | ND | 133752 | 123364 | ND | ND | 91044 | ND |
| 12:52 | 180.1146 | Resorcinol, pentadecyl- | C_21_H_36_O_2_ | ND | ND | 173453 | ND | ND | ND | ND | ND |
| 12:54 | 181.1184 | Acetic acid, 3-acetylcyclohexyl ester | C_10_H_16_O_3_ | ND | ND | ND | ND | ND | ND | 889649 | ND |
| 12:54 | 180.1148 | Acetic acid, [4-[(acetyloxy)methyl]-1-(4-amino-1,2,5-oxadiazol-3-yl)-1H-1,2,3-triazol-5-yl]methyl ester | C_10_H_12_N_6_O_5_ | ND | ND | ND | ND | ND | ND | 714567 | ND |
| 12:58 | 207.1748 | Tetradecadien-3-one,1,13- | C_14_H_24_O | ND | ND | 415948 | ND | ND | ND | ND | ND |
| 12:58 | 204.1875 | 1,13-Tetradecadien-3-one | C_14_H_24_O | ND | ND | 432405 | ND | ND | ND | 342410 | ND |
| 12:59 | 208.1774 | (3S,3aR,3bR,4S,7R,7aR)-4-Isopropyl-3,7-dimethyloctahydro-1H-cyclopenta[1,3]cyclopropa[1,2]benzen-3-ol | C_15_H_26_O | ND | ND | ND | 152791 | ND | ND | ND | ND |
| 13:03 | 155.1758 | 2-Pyrrolidinecarboxylic acid-5-oxo-, ethyl ester | C_7_H_11_NO_3_ | ND | ND | 57422 | ND | ND | ND | ND | ND |
| 13:04 | 150.1390 | 1-Butanol, 2-methyl-, propionate | C_8_H_16_O_2_ | ND | ND | ND | ND | 163855 | ND | ND | ND |
| 13:04 | 207.1655 | (-)-Spathulenol | C_15_H_24_O | ND | ND | ND | ND | 430630 | ND | 637453 | ND |
| 13:04 | 206.1623 | 7-Heptadecene, 1-chloro- | C_17_H_33_Cl | ND | ND | ND | 1440066 | ND | 652401 | ND | ND |
| 13:04 | 206.1622 | Cetene | C_16_H_32_ | ND | ND | ND | 1283653 | ND | ND | ND | ND |
| 13:04 | 205.1591 | 5-Octadecene, (E)- | C_18_H_36_ | ND | ND | 2863342 | ND | ND | ND | ND | ND |
| 13:04 | 207.1653 | Cholesta-8,24-dien-3-ol, 4-methyl-, (3á,4à)- | C_28_H_46_O | ND | ND | ND | ND | ND | ND | 1191384 | ND |
| 13:08 | 166.0989 | 9-Bromononanoic acid, methyl(ester) | C_10_H_19_BrO_2_ | ND | ND | ND | 171762 | ND | ND | ND | ND |
| 13:08 | 166.0991 | Methyl 10-oxo-8-decenoate | C_11_H_18_O_3_ | ND | ND | ND | 178838 | ND | ND | ND | ND |
| 13:09 | 220.1460 | Methyl 16-hydroxy-hexadecanoate | C_17_H_34_O_3_ | ND | ND | ND | ND | ND | ND | 140649 | ND |
| 13:09 | 220.1457 | Tridecanedioic acid, dimethyl ester | C_15_H_28_O_4_ | ND | ND | ND | ND | ND | ND | 119118 | ND |
| 13:10 | 202.1710 | Pentadecane | C_15_H_32_ | 6808748 | ND | 942776 | 4529977 | 3722399 | ND | ND | ND |
| 13:11 | 252.9117 | Eicosane | C_20_H_42_ | 6541817,5 | 522996 | 16105081 | 9950235 | 7426897 | 2971426 | 4048830 | ND |
| 13:12 | 177.0545 | Phthalic acid, monoamide, N-ethyl-N-(3-methylphenyl)-, ethyl ester | C_19_H_21_NO_3_ | ND | 33348 | ND | ND | ND | ND | ND | ND |
| 13:12 | 203.1443 | Sulfurous acid, butyl pentyl ester | C_9_H_20_O_3_S | 0 | ND | 50140454 | ND | ND | ND | ND | ND |
| 13:12 | 202.1710 | Diethyl Phthalate | C_12_H_14_O_4_ | 47657 | ND | ND | ND | 143230 | ND | 83288 | ND |
| 13:12 | 243.1603 | Fumaric acid, nonyl tetrahydrofurfuryl ester | C_18_H_30_O_5_ | ND | ND | ND | 200795 | ND | ND | ND | ND |
| 13:12 | 187.0699 | Bicyclo[4.2.0]octan-7-one, 8,8-dichloro- | C_8_H_10_Cl_2_O | ND | ND | ND | ND | ND | 190200 | ND | ND |
| 13:12 | 173.1173 | 2-Undecen-4-ol | C_11_H_22_O | 74481 | ND | 17105342 | 742288 | 106030 | 4587981 | ND | ND |
| 13:13 | 114.9950 | 3-Methoxyhex-1-ene | C_7_H_14_O | ND | ND | ND | 411193 | ND | ND | ND | ND |
| 13:14 | 225.2533 | Fumaric acid, ethyl tetrahydrofurfuryl ester | C_11_H_16_O_5_ | ND | ND | 563256 | ND | ND | ND | ND | ND |
| 13:16 | 218.2027 | 1-Isobutyladamantane | C_14_H_24_ | ND | ND | 131902 | ND | ND | ND | ND | ND |
| 13:16 | 149.1324 | Alanine, 3,3,3-trifluoro-2-[(4-methoxybenzoyl)amino]-N-[3-(trifluoromethyl)-2-quinoxalinyl]-, ethyl ester | C_22_H_18_F_6_N_4_O_4_ | ND | ND | 109702 | ND | ND | ND | ND | ND |
| 13:17 | 160.1226 | Propane, 1,1,3,3-tetramethoxy- | C_7_H_16_O_4_ | ND | ND | ND | ND | ND | ND | 79336 | ND |
| 13:17 | 191.1433 | 1,1-Dimethoxy-2-phenylpropane | C_11_H_16_O_2_ | ND | ND | ND | ND | ND | ND | 74778 | ND |
| 13:20 | 197.0771 | 5-tert-Butyl-2-[[(2,5-dimethyl-furan-3-carbonyl)-amino]-methyl]-furan-3-carboxylic acid | C_17_H_21_NO_5_ | ND | ND | ND | ND | ND | ND | 87375 | ND |
| 13:21 | 196.0732 | Pentamethylbenzyl chloride | C_12_H_17_Cl | 20507 | ND | ND | ND | ND | ND | ND | ND |
| 13:26 | 153.1272 | 2-Propenylcyclopropanecarboxylic acid, ethyl ester | C_9_H_14_O_2_ | ND | ND | ND | 168090 | ND | ND | ND | ND |
| 13:26 | 138.0547 | 7-Azabicyclo[4.1.0]heptane, 2,4,4-trimethyl- | C_9_H_17_N | ND | ND | ND | 155367 | ND | ND | ND | ND |
| 13:27 | 194.1302 | Hexanoic acid, tridec-2-ynyl ester | C_19_H_34_O_2_ | ND | ND | ND | ND | ND | 252904 | ND | ND |
| 13:30 | 172.9328 | Sulfurous acid, dipentyl ester | C_10_H_22_O_3_S | ND | ND | ND | ND | ND | 550324 | ND | ND |
| 13:30 | 153.0466 | Tetrahydrofuran, 2-hexyl- | C_10_H_20_O | ND | ND | ND | ND | ND | ND | ND | ND |
| 13:31 | 177.1275 | 1-Propanone, 1-[2-(1,1-dimethylethyl)cyclopropyl]-2,2-dimethyl-, trans- | C_12_H_22_O | ND | ND | 140590 | ND | ND | ND | ND | ND |
| 13:31 | 154.1343 | Cyclobutylcarboxamide, N-methallyl- | C_9_H_15_NO | ND | ND | 109518 | ND | ND | ND | ND | ND |
| 13:35 | 201.1846 | Dodecanoic acid methylethyl ester | C_15_H_30_O_2_ | ND | ND | 212837 | ND | ND | ND | ND | ND |
| 13:35 | 222.2341 | Dodecanoic acid, 1-methylethyl ester | C_15_H_30_O_2_ | ND | ND | 223965 | 106004 | ND | ND | ND | ND |
| 13:40 | 169.0886 | 2-Aminobiphenyl | C_12_H_11_N | ND | ND | 38204 | ND | ND | ND | ND | ND |
| 13:43 | 182.0725 | N,N Dimethyl-3,4-methylenedioxyamphetamine | C_12_H_17_NO_2_ | ND | ND | 128995 | ND | ND | ND | ND | ND |
| 13:43 | 254.0179 | 2-Propanol, 1-(isopropylamino)-3-(p-tolylthio)- | C_13_H_21_NOS | ND | ND | 143354 | ND | ND | ND | ND | ND |
| 13:48 | 182.0726 | Benzophenone | C_13_H_10_O | ND | ND | 156958 | ND | ND | ND | ND | ND |
| 13:48 | 178.0624 | 6-Methoxychromanone | C_10_H_10_O_3_ | ND | ND | ND | 166080 | ND | ND | ND | ND |
| 13:48 | 146.9762 | Butyric acid, 3-tridecyl ester | C_17_H_34_O_2_ | ND | ND | ND | ND | ND | ND | ND | ND |
| 13:48 | 199.0888 | Hexano-dibutyrin | C_17_H_30_O_6_ | ND | ND | ND | ND | ND | 149049 | ND | ND |
| 13:49 | 196.0916 | Acetoxyacetic acid, 1-adamantylmethyl ester | C_15_H_22_O_4_ | ND | ND | ND | ND | ND | ND | 40793 | ND |
| 13:50 | 196.2180 | Tridecane | C_13_H_28_ | ND | ND | 744650 | ND | 351512 | 4115173 | 4859732 | ND |
| 13:52 | 193.1225 | Pregna-5,8-diene-3á,11à-diol-20-one diacetate | C_25_H_34_O_5_ | ND | ND | ND | ND | ND | ND | 1127685 | ND |
| 13:53 | 181.1226 | Peak 69Tricyclo[4.3.1.1(3,8)]undecane, 1-methoxy- | C_12_H_20_O | ND | ND | ND | ND | ND | ND | 905285 | ND |
| 13:57 | 205.1583 | 10,10-Dimethyl-2,6-dimethylenebicyclo[7.2.0]undecan-5á-ol | C_15_H_24_O | ND | ND | ND | ND | ND | ND | 352358 | ND |
| 13:57 | 190.1345 | 11,11-Dimethyl-4,8-dimethylenebicyclo[7.2.0]undecan-3-ol | C_15_H_24_O | ND | ND | ND | ND | ND | ND | 273045 | ND |
| 14:01 | 165.1024 | Octacosane | C_28_H_58_ | 511386,5 | ND | 1306328 | 1193479 | 2653485 | 392471 | 266030 | ND |
| 14:02 | 211.2426 | Heneicosane, 11-(1-ethylpropyl)- | C_26_H_54_ | ND | ND | 1715201 | 999054 | 136020 | ND | ND | ND |
| 14:02 | 141.1637 | Nonane, 2-methyl- | C_10_H_22_ | ND | ND | ND | ND | ND | ND | 379775 | ND |
| 14:02 | 113.1326 | Hexane, 3,3-dimethyl- | C_8_H_18_ | ND | ND | ND | ND | ND | ND | 284412 | ND |
| 14:03 | 208.0336 | 1-Naphthalenol, 1,2,3,4,4a,7,8,8a-octahydro-1,6-dimethyl-4-(1-methylethyl)-, [1S-(1à,4à,4aá,8aá)]- | C_15_H_26_O | 101344 | ND | ND | ND | ND | ND | ND | ND |
| 14:07 | 195.0943 | Ylangenol | C_15_H_24_O | 86212 | ND | ND | ND | ND | ND | ND | ND |
| 14:10 | 458.9919 | Cyclooctasiloxane, hexadecamethyl- | C_16_H_48_O_8_Si_8_ | ND | 993464 | 181003 | ND | 136738 | ND | ND | ND |
| 14:13 | 206.1302 | 4,4,5,8-Tetramethylchroman-2-ol | C_13_H_18_O_2_ | ND | ND | ND | 35301 | ND | ND | ND | ND |
| 14:13 | 188.1194 | 1-[4-(2-Hydroxybut-3-yn-2-yl)phenyl]ethanone | C_12_H_12_O_2_ | ND | ND | ND | ND | ND | ND | 626816 | ND |
| 14:13 | 206.1303 | 4,4,5,8-Tetramethylchroman-2-ol | C_13_H_18_O_2_ | ND | ND | ND | ND | ND | ND | 97086 | ND |
| 14:13 | 200.1559 | 4-Isopropyl-6-methyl-1-methylene-1,2,3,4-tetrahydronaphthalene | C_15_H_20_ | 64740 | ND | ND | ND | ND | ND | ND | ND |
| 14:14 | 236.2497 | 7-Hexadecyne | C_16_H_30_ | ND | ND | ND | ND | ND | 532226 | ND | ND |
| 14:14 | 182.0957 | 8-Dodecen-1-ol, (Z)- | C_12_H_24_O | ND | ND | 1504936 | ND | ND | ND | ND | ND |
| 14:14 | 182.0574 | Benzaldehyde, 4-hydroxy-3,5-dimethoxy- | C_9_H_10_O_4_ | ND | ND | ND | ND | ND | ND | 62715 | ND |
| 14:14 | 249.1105 | 11,14-Eicosadienoic acid, methyl ester | C_21_H_38_O_2_ | ND | ND | 2931244 | ND | ND | ND | ND | ND |
| 14:14 | 197.1196 | Isothiazole-4-carbonitrile, 3,5-bis[(2-dimemhylamino)ethylthio]- | C_12_H_20_N_4_S_3_ | ND | ND | ND | 172276 | ND | ND | ND | ND |
| 14:14 | 182.0574 | 6,8-Dodecadien-1-ol (6Z,8E) | C_12_H_22_O | ND | ND | ND | ND | 123692 | ND | ND | ND |
| 14:14 | 142.0776 | 5-Hepten-2-amine, N,6-dimethyl- | C_9_H_19_N | ND | ND | ND | 209351 | ND | ND | ND | ND |
| 14:15 | 168.0415 | Cyclobutanecarboxylic acid, 2-dimethylaminoethyl ester | C_9_H_17_NO_2_ | ND | ND | ND | ND | 160093 | ND | ND | ND |
| 14:15 | 249.1113 | Dimantine | C_20_H_43_N | ND | ND | ND | ND | ND | ND | ND | ND |
| 14:19 | 218.2024 | Octacosyl heptafluorobutyrate | C_32_H_57_F_7_O_2_ | ND | ND | ND | ND | ND | 161031 | ND | ND |
| 14:19 | 238.2658 | 3-Eicosene, (E)- | C_20_H_40_ | ND | ND | 250728 | 840857 | 200915 | ND | ND | ND |
| 14:19 | 262.7224 | Nonacos-1-ene | C_29_H_58_ | ND | ND | ND | 287075 | ND | ND | ND | ND |
| 14:19 | 200.1555 | 9,12,15-Octadecatrienoic acid, 2,3-bis(acetyloxy)propyl ester, (Z,Z,Z)- | C_25_H_40_O_6_ | 42761 | ND | 6182566 | ND | ND | ND | ND | ND |
| 14:20 | 92.0623 | Toluene | C_7_H_8_ | ND | 41223 | ND | ND | DN | ND | ND | ND |
| 14:23 | 199.9374 | Fluorene, 9-chloro- | C_13_H_9_Cl | ND | ND | 34581 | ND | ND | ND | ND | ND |
| 14:25 | 213.1597 | Adipic acid, cyclohexyl isobutyl ester | C_16_H_28_O_4_ | ND | ND | ND | 69779 | ND | ND | ND | ND |
| 14:25 | 191.1795 | Adipic acid, isobutyl 3-pentyl ester | C_15_H_28_O_4_ | ND | ND | ND | ND | ND | ND | ND | ND |
| 14:25 | 212.1564 | 1,3-di-iso-propylnaphthalene | C_16_H_20_ | 28059 | ND | 190662 | 42199 | ND | ND | ND | ND |
| 14:26 | 212.1563 | 1,7-di-iso-propylnaphthalene | C_16_H_20_ | ND | ND | 67366 | ND | ND | ND | ND | ND |
| 14:27 | 145.1222 | Acetic acid, 5-hydroxy-pent-3-ynyl ester | C_7_H_10_O_3_ | ND | ND | ND | ND | ND | ND | 980128 | ND |
| 14:29 | 196.1207 | Pyridine, 2-[2-(4-aminophenyl)ethenyl]-, trans- | C_13_H_12_N_2_ | ND | ND | 70223 | ND | ND | ND | ND | ND |
| 14:30 | 156.0791 | p-Menthan-1-ol, trans- | C_10_H_20_O | ND | ND | ND | ND | ND | 120826 | ND | ND |
| 14:30 | 156.0796 | p-Menthan-4-ol | C_10_H_20_O | ND | ND | ND | ND | ND | 130923 | ND | ND |
| 14:30 | 212.1561 | 4a-Methyl-1-methylene-1,2,3,4,4a,9,10,10a-octahydrophenanthrene | C_16_H_20_ | 41804 | ND | ND | ND | ND | ND | ND | ND |
| 14:35 | 260.7236 | 4-Fluorobenzoic acid, tridec-2-ynyl ester | C_20_H_27_FO_2_ | ND | ND | ND | ND | ND | ND | 471042 | ND |
| 14:35 | 191.1062 | 1-Benzoxirene, 5a-[3-oxo-1-butenyl]perhydro-2-hydroxy-1a,5,5-trimethyl-, acetate | C_15_H_22_O_4_ | ND | ND | ND | ND | ND | ND | 367501 | ND |
| 14:36 | 127.1480 | Carbonic acid, decyl prop-1-en-2-yl ester | C_14_H_26_O_3_ | ND | ND | ND | ND | ND | 275523 | ND | ND |
| 14:3 | 240.2809 | Carbonic acid, prop-1-en-2-yl tridecyl ester | C_17_H_32_O_3_ | ND | ND | ND | 2141782 | ND | ND | ND | ND |
| 14:36 | 193.1598 | Carbonic acid, prop-1-en-2-yl tetradecyl ester | C_18_H_34_O_3_ | 171775 | ND | ND | ND | ND | ND | ND | ND |
| 14:37 | 226.2292 | 2-[2-Benzyloxy-1-(1-methoxy-ethoxy)-ethyl]-oxirane | C_14_H_20_O_4_ | ND | ND | ND | 570373 | ND | ND | ND | ND |
| 14:37 | 193.1223 | 2-Dodecanone | C_12_H_24_O | ND | ND | ND | ND | 252561 | ND | ND | ND |
| 14:37 | 193.1289 | O-Methyl S-2-dimethylaminoethyl ethylphosphonothioate | C_7_H_18_NO_2_PS | 118681 | ND | ND | ND | ND | ND | ND | ND |
| 14:3 | 220.1823 | (1R,7S,E)-7-Isopropyl-4,10-dimethylenecyclodec-5-enol | C_15_H_24_O | ND | ND | ND | ND | ND | ND | 583091 | ND |
| 14:41 | 183.2105 | Butanoic acid, 2-methyl-5-oxo-1-cyclopenten-1-yl ester | C_10_H_14_O_3_ | ND | ND | 484807 | ND | ND | ND | ND | ND |
| 14:41 | 183.2101 | Undecane, 3,6-dimethyl- | C_13_H_28_ | ND | ND | ND | 244794 | ND | ND | ND | ND |
| 14:50 | 131.0160 | Benzaldehyde, 3-chloro-5-methoxy-4-[(4-methylphenyl)methoxy]- | C_16_H_15_ClO_3_ | ND | ND | ND | 90028 | ND | ND | ND | ND |
| 14:50 | 162.1398 | Benzoic acid, 2-ethylhexyl ester | C_15_H_22_O_2_ | ND | ND | 170326 | ND | ND | ND | ND | ND |
| 14:50 | 180.1874 | Benzoic acid, 2-methylbutyl ester | C_12_H_16_O_2_ | ND | ND | 181968 | ND | ND | ND | ND | ND |
| 14:50 | 287.8776 | Ethylhexyl benzoate | C_15_H_22_O_2_ | ND | ND | ND | 87925 | ND | ND | ND | ND |
| 14:53 | 210.1405 | 1,1'-Biphenyl, 2,2',5,5'-tetramethyl- | C_16_H_18_ | ND | ND | 87083 | 44491 | ND | ND | ND | ND |
| 14:58 | 234.1975 | Methyl tetradecanoate | C_15_H_30_O_2_ | ND | ND | ND | 523301 | ND | 334884 | ND | ND |
| 14:59 | 222.1974 | 2-Adamantanol, 2-(bromomethyl)- | C_11_H_17_BrO | ND | ND | 326679 | ND | ND | ND | ND | ND |
| 14:59 | 186.0432 | Dimethyl-(allyl)-silyloxybenzene | C_11_H_16_OSi | ND | ND | 291765 | ND | ND | ND | ND | ND |
| 15:01 | 212.1561 | 1,4-di-iso-propylnaphthalene | C_16_H_20_ | ND | ND | 136489 | ND | ND | ND | ND | ND |
| 15:02 | 169.1014 | Decane, 5-methyl-6-methylene- | C_12_H_24_ | ND | ND | 164480 | ND | ND | ND | ND | ND |
| 15:02 | 140.1561 | 2-(Diethylamino)butyronitrile | C_8_H_16_N_2_ | ND | ND | ND | ND | ND | ND | 318024 | ND |
| 15:02 | 140.1561 | Boraneamine, N-ethyl-1,1-dipropyl- ; | C_8_H_20_BN | ND | ND | ND | ND | ND | ND | 365507 | ND |
| 15:02 | 175.1248 | Heptacosane, 1-chloro- | C_27_H_55_Cl | ND | ND | ND | ND | 497130 | ND | ND | ND |
| 15:02 | 175.1249 | Sulfurous acid, hexadecyl pentyl ester | C_21_H_44_O_3_S | ND | ND | ND | ND | 606299 | ND | ND | ND |
| 15:02 | 57.0700 | Isocyanatomethane | C_2_H_3_NO | ND | ND | ND | ND | ND | ND | ND | ND |
| 15:02 | 203.0702 | Sulfurous acid, 2-ethylhexyl heptadecyl ester | C_25_H_52_O_3_S | ND | ND | ND | 295061 | ND | ND | 740968 | ND |
| 15:03 | 183.2103 | Sulfurous acid, 2-ethylhexyl pentadecyl ester | C_23_H4_8_O_3_S | ND | ND | ND | ND | ND | ND | 979945 | ND |
| 15:03 | 184.0843 | Benzene, (1-ethylhexyl)- | C_14_H_22_ | ND | ND | 156742 | ND | ND | ND | ND | ND |
| 15:04 | 222.1618 | 1,4-Benzenediol, 2,5-bis(1,1-dimethylethyl)- | C_14_H_22_O_2_ | ND | ND | ND | 160551 | ND | ND | ND | ND |
| 15:05 | 166.0966 | 8-Oxabicyclo[3.2.1]oct-6-en-2-one, 1,4,4-trimethyl- | C_10_H_14_O_2_ | 0 | ND | ND | ND | ND | ND | 2159997 | ND |
| 15:06 | 191.1432 | 3-n-Hexylthiane, S,S-dioxide | C_11_H_22_O_2_S | ND | ND | ND | ND | 146889 | ND | ND | ND |
| 15:06 | 223.1647 | Pentadecafluorooctanoic acid, dodecyl ester | C_20_H_25_F_1_5O_2_ | ND | ND | ND | ND | ND | ND | 238940 | ND |
| 15:07 | 212.1571 | 1,4-di-iso-propylnaphthalene | C_16_H_20_ | 80586 | ND | 210542 | ND | ND | ND | ND | ND |
| 15:09 | 166.0962 | 2-Fluoroimidazole-5-propenoic acid, methyl(ester) | C_7_H_7_FN_2_O_2_ | ND | ND | ND | ND | ND | ND | 69541 | ND |
| 15:11 | 198.1364 | L-Alanine, N-(trifluoroacetyl)-, butyl ester | C_9_H_14_F_3_NO_3_ | ND | ND | 55299 | ND | ND | ND | ND | ND |
| 15:12 | 225.2572 | Heptadecane, 2,6-dimethyl- | C_19_H_40_ | ND | ND | 1986220 | ND | ND | ND | ND | ND |
| 15:12 | 287.8793 | Sulfurous acid, decyl 2-ethylhexyl ester | C_18_H_38_O_3_S | ND | ND | ND | 958276 | ND | ND | ND | ND |
| 15:12 | 183.2106 | Undecane, 2,5-dimethyl- | C_13_H_28_ | ND | ND | 1974384 | ND | ND | ND | ND | ND |
| 15:12 | 181.0493 | Undecane, 6-ethyl- | C_13_H_28_ | ND | ND | ND | ND | ND | ND | 327013 | ND |
| 15:19 | 220.1815 | 1,1,4,7-Tetramethyldecahydro-1H-cyclopropa[e]azulene-4,7-diol | C_15_H_26_O_2_ | ND | ND | ND | ND | ND | ND | 256511 | ND |
| 15:19 | 220.1821 | Aromadendrane-4,10-diol | C_15_H_26_O_2_ | ND | ND | ND | ND | 222790 | ND | 203030 | ND |
| 15:20 | 181.0860 | 9-Desoxo-9-x-acetoxy-3-desoxy-7.8.12-tri-O-acetylingol-3-one | C_28_H_38_O_1_0 | ND | ND | ND | ND | 48104 | ND | ND | ND |
| 15:20 | 192.0422 | 2(4H)-Benzofuranone, 5,6,7,7a-tetrahydro-6-hydroxy-4,4,7a-trimethyl- | C_11_H_16_O_3_ | ND | ND | ND | 51984 | ND | ND | ND | ND |
| 15:29 | 171.1378 | Nonanoic acid | C_9_H_18_O_2_ | ND | ND | ND | ND | ND | ND | 117544 | ND |
| 15:32 | 225.2578 | Eicosane, 2-methyl- | C_21_H_44_ | ND | ND | 1326043 | 690240 | ND | ND | ND | ND |
| 15:32 | 226.2614 | Heptadecane, 2,6,10,15-tetramethyl- | C_21_H_44_ | ND | ND | ND | 863554 | ND | ND | ND | ND |
| 15:32 | 225.2574 | Nonadecane, 2-methyl- | C_20_H_42_ | ND | ND | 1334580 | ND | ND | ND | ND | ND |
| 15:35 | 223.0836 | Pregnan-20-one, 3-(acetyloxy)-5,6-epoxy-6-methyl-, cyclic 20-(1,2-ethanediyl acetal), (3á,5à,6à)- | C_26_H_40_O_5_ | ND | ND | ND | ND | ND | ND | 122128 | ND |
| 15:36 | 234.1616 | 3,5-di-tert-Butyl-4-hydroxybenzaldehyde | C_15_H_22_O_2_ | ND | ND | 43038 | 31315 | ND | ND | ND | ND |
| 15:37 | 212.0835 | Benzyl Benzoate | C_14_H_12_O_2_ | 3138954,5 | ND | 1028475 | 985360 | 237274 | ND | 375800 | ND |
| 15:37 | 213.0864 | (+-)-N-Benzyl-alpha-methyl-N-nitrosobenzylamine | C_15_H_1_6N_2_O | ND | ND | ND | ND | ND | ND | 285436 | ND |
| 15:38 | 166.1344 | 2-Undecenal, E- | C_11_H_20_O | ND | ND | 824136 | ND | 421738 | ND | ND | ND |
| 15:42 | 196.1086 | 6-Hydroxy-4,4,7a-trimethyl-5,6,7,7a-tetrahydrobenzofuran-2(4H)-one | C_11_H_16_O_3_ | 44534 | ND | ND | 357686 | 358266 | ND | 2474206 | ND |
| 15:46 | 262.7221 | Pentadecanoic acid, methyl ester | C_16_H_32_O_2_ | ND | ND | ND | 73310 | ND | ND | ND | ND |
| 15:47 | 154.1716 | Acethydrazide, N2-(4-dimethylaminobenzylidene)-2-(1-piperidyl)- | C_16_H_24_N_4_O | 5477 | ND | ND | ND | ND | ND | ND | ND |
| 15:48 | 192.1259 | 5-Eicosene, (E)- | C_20_H_40_ | 307289 | ND | ND | 827248 | 247164 | ND | ND | ND |
| 15:48 | 161.0730 | 9-Eicosene, (E)- | C_20_H_40_ | 190102 | ND | ND | 1360810 | ND | ND | ND | ND |
| 15:48 | 154.1719 | E-15-Heptadecenal | C_17_H_32_O | ND | ND | ND | ND | ND | ND | ND | ND |
| 15:48 | 181.1948 | 3-Octadecene, (E)- | C_18_H_36_ | ND | ND | 2248903 | 1554346 | ND | ND | 544719 | ND |
| 15:49 | 146.1073 | Cycloeicosane | C_20_H_40_ | ND | ND | ND | ND | ND | ND | 482053 | ND |
| 15:53 | 178.0779 | 9H-Fluorene, 9-methylene- | C_14_H_10_ | ND | ND | 168971 | ND | ND | ND | 41560 | ND |
| 15:53 | 178.0781 | Diphenylacetylene | C_14_H_10_ | ND | ND | 185665 | ND | ND | ND | ND | ND |
| 15:53 | 178.0779 | Phenanthrene | C_14_H_10_ | ND | ND | ND | 117881 | 64712 | ND | ND | ND |
| 15:56 | 194.1298 | 3,10-Dioxatricyclo[4.3.1.0(2,4)]decan-7-ol, acetate, (1à,2à,4à,6à,7á)- | C_10_H_14_O_4_ | ND | ND | ND | ND | ND | 241139 | ND | ND |
| 15:57 | 194.0928 | 1-[3-(2-Bromophenyl)-2-thioureido]-1-deoxy-á-d-glucopyranose 2,3,4,6-tetraacetate | C_21_H_25_BrN_2_O_9_S | ND | ND | ND | ND | ND | ND | 1448345 | ND |
| 15:58 | 214.0987 | (3S,5R,7aS)-3-Heptyl-5-methylhexahydro-1H-pyrrolizine | C1_5_H_29_N | ND | ND | ND | ND | ND | ND | 368124 | ND |
| 15:59 | 181.0679 | 3-Methyl-5-nonylpyrrolizidine | C_17_H_33_N | ND | ND | ND | ND | ND | ND | 312082 | ND |
| 16:13 | 230.2196 | Isopropyl myristate | C_17_H_34_O_2_ | 75609 | ND | 433491 | 465668 | 139143 | ND | 116083 | ND |
| 16:13 | 475.0038 | Octasiloxane, 1,1,3,3,5,5,7,7,9,9,11,11,13,13,15,15-hexadecamethyl- | C_16_H_50_O_7_Si_8_ | ND | 627730 | ND | ND | ND | ND | ND | 81804 |
| 16:13 | 431.0870 | Heptasiloxane, 1,1,3,3,5,5,7,7,9,9,11,11,13,13-tetradecamethyl- | C_14_H_44_O_6_Si_7_ | ND | ND | ND | 128163 | 142662 | ND | ND | ND |
| 16:27 | 250.2662 | 2-Pentadecanone, 6,10,14-trimethyl- | C_18_H_36_O | 489273,5 | 78570 | 2687860 | 4045852 | 4765580 | 309381 | ND | ND |
| 16:27 | 194.2029 | 2-Undecanone, 6,10-dimethyl- | C_13_H_26_O | ND | ND | ND | ND | ND | ND | 3840793 | ND |
| 16:30 | 140.1197 | 2,3-Dimethyl-2-octene | C_10_H_20_ | ND | ND | 242537 | ND | ND | ND | ND | ND |
| 16:30 | 140.1198 | 4-Cyclopentene-1,3-dione, 4-methoxy-5-methyl- | C_7_H_8_O_3_ | ND | ND | 219274 | ND | ND | ND | ND | ND |
| 16:33 | 219.2107 | Dihydroobscurinervidinediol | C_24_H_34_N_2_O_5_ | ND | ND | 252794 | ND | ND | ND | ND | ND |
| 16:45 | 223.0971 | 1,2-Benzenedicarboxylic acid, bis(2-methylpropyl) ester | C_16_H_22_O_4_ | 242114 | ND | ND | ND | ND | ND | ND | ND |
| 16:45 | 150.0272 | Phthalic acid, 4-cyanophenyl heptyl ester | C_22_H_23_NO_4_ | ND | 50392 | ND | ND | ND | ND | ND | ND |
| 16:46 | 224.0998 | 1,2-Benzenedicarboxylic acid, diheptyl ester | C_22_H_34_O_4_ | ND | ND | ND | 2214310 | ND | ND | ND | ND |
| 16:46 | 224.1006 | Phthalic acid, hex-2-yn-4-yl isobutyl ester | C_18_H_22_O_4_ | ND | ND | 1446889 | ND | 446171 | ND | ND | ND |
| 16:46 | 223.0978 | Phthalic acid, 8-bromoctyl isobutyl ester | C_20_H_29_BrO_4_ | 237016 | ND | 1701665 | 1857134 | 488495 | ND | ND | ND |
| 16:46 | 150.0267 | Phthalic acid, hex-2-yn-4-yl nonyl ester | C_23_H_32_O_4_ | ND | ND | ND | ND | ND | 68379 | ND | ND |
| 16:47 | 223.0962 | Phthalic acid, heptyl 4-nitrophenyl ester | C_21_H_23_NO_6_ | ND | ND | ND | ND | ND | ND | 332684 | ND |
| 16:50 | 164.8233 | Hexanamide, N-(2-hydroxyethyl)- | C_8_H_17_NO_2_ | ND | ND | ND | 35843 | ND | ND | ND | ND |
| 16:50 | 157.1012 | Z-10-Tetradecen-1-ol acetate | C_16_H_30_O_2_ | ND | ND | 95104 | ND | ND | ND | ND | ND |
| 16:50 | 164.1188 | 1-Undecanol | C_11_H_24_O | ND | ND | ND | ND | ND | ND | ND | ND |
| 16:52 | 278.2970 | Neophytadiene | C_20_H_38_ | ND | ND | ND | ND | ND | ND | 332721 | 380250 |
| 16:55 | 227.2003 | 2,2,3,3,6,6-Hexafluoro-5-(2,2,2-trifluoro-1-trifluoromethylethoxy)-3,6-dihydro-2H-[1,4]oxazine | C_7_HF_12_NO_2_ | ND | ND | ND | 48977 | ND | ND | ND | ND |
| 16:55 | 227.2003 | 3-(([5-(2-Chloroethoxy)-1,1,3,3,5,5-hexamethyltrisiloxanyl]oxy)methyl)pyridine | C_14_H_28_ClNO_4_Si_3_ | ND | ND | ND | 33378 | ND | ND | 81408 | ND |
| 16:57 | 255.9602 | Oxalic acid, 6-ethyloct-3-yl ethyl ester | C_14_H_26_O_4_ | ND | ND | ND | 227297 | ND | ND | ND | ND |
| 16:58 | 84.0570 | Amitrol | C_2_H_4_N_4_ | ND | ND | ND | 330448 | ND | ND | ND | ND |
| 16:58 | 222.0886 | Butanoic acid, 6-ethyl-3-octyl ester | C_14_H_28_O_2_ | ND | ND | ND | ND | ND | ND | 288193 | ND |
| 16:58 | 165.1276 | Hexane, 1-(pentyloxy)- | C_11_H_24_O | ND | ND | 179102 | ND | ND | ND | ND | ND |
| 17:02 | 156.0930 | Heptadecane | C_17_H_36_ | 295808 | ND | ND | 270223 | ND | ND | ND | ND |
| 17:03 | 135.1173 | Bicyclo[3.1.1]heptan-3-one, 2-hydroxy-2,6,6-trimethyl- | C_10_H_16_O_2_ | 128138 | ND | ND | ND | ND | ND | ND | ND |
| 17:04 | 235.1524 | Acetamide, 2-chloro-N-(ethoxymethyl)-N-(2-ethyl-6-methylphenyl)- | C_14_H_20_ClNO_2_ | ND | ND | 169229 | 91267 | 45408 | ND | ND | ND |
| 17:05 | 111.1171 | 2-Tetradecanone | C_14_H_28_O | ND | 51405 | ND | ND | ND | ND | 288395 | ND |
| 17:05 | 254.2603 | 2-Nonadecanone | C_19_H_38_O | ND | ND | ND | ND | 1107354 | ND | 603944 | ND |
| 17:05 | 239.2374 | 2-Pentadecanone | C_15_H_30_O | 489259,5 | ND | 4717467 | 3378603 | ND | 227362 | 731326 | ND |
| 17:05 | 234.1491 | Acetochlor | C_14_H_20_ClNO_2_ | ND | ND | ND | ND | ND | ND | 7464 | ND |
| 17:15 | 172.1239 | O-(2-(Dimethylamino)ethyl)-2-chlorophenyl-3-chlorophenylketoxime | C_17_H_18_Cl_2_N_2_O | ND | ND | ND | ND | ND | ND | 268530 | ND |
| 17:15 | 164.0825 | 4-fluoromethamphetamine | C_10_H_14_FN | ND | ND | ND | ND | ND | ND | 242731 | ND |
| 17:19 | 189.1026 | Succinic acid, tridec-2-yn-1-yl 3-methylbut-2-yl ester | C_22_H_38_O_4_ | 225675 | ND | ND | ND | ND | ND | ND | ND |
| 17:19 | 163.1484 | Succinic acid, tridec-2-yn-1-yl 3-methylbut-2-yl ester | C_22_H_38_O_4_ | ND | ND | ND | ND | ND | 119163 | ND | ND |
| 17:19 | 249.2582 | 1-Decanol, 2-hexyl- | C_16_H_34_O | ND | ND | ND | ND | 2872859 | ND | 5743408 | ND |
| 17:19 | 223.2381 | 1-Dodecanol, 3,7,11-trimethyl- | C_15_H_32_O | ND | ND | ND | 5105984 | ND | ND | 7073935 | ND |
| 17:20 | 270.2560 | Hexadecanoic acid, methyl ester | C_17_H_34_O_2_ | 2230202 | 674773 | 6491377 | 12299726 | 4291414 | 1251499 | ND | ND |
| 17:22 | 262.1522 | 7,9-Di-tert-butyl-1-oxaspiro(4,5)deca-6,9-diene-2,8-dione | C_17_H_24_O_3_ | ND | ND | 38613 | 65047 | 37549 | ND | 71631 | ND |
| 17:29 | 190.0767 | Heptacosane | C_27_H_56_ | ND | ND | 537246 | 558054 | ND | ND | 705939 | ND |
| 17:31 | 196.2181 | 3,4-dimethyl-5-(3,4-methylenedioxy)phenyloxazolidine | C_12_H_15_NO_3_ | ND | ND | ND | 629324 | ND | ND | ND | ND |
| 17:31 | 196.2185 | Dodecane, 4,6-dimethyl- | C_14_H_30_ | ND | ND | ND | 696677 | ND | ND | ND | ND |
| 17:35 | 292.2028 | Benzenepropanoic acid, 3,5-bis(1,1-dimethylethyl)-4-hydroxy-, methyl ester | C_18_H_28_O_3_ | 78336,5 | 33753 | 767884 | 355448 | ND | ND | 154491 | ND |
| 17:38 | 194.1056 | 5,10-Diethoxy-2,3,7,8-tetrahydro-1H,6H-dipyrrolo[1,2-a:1',2'-d]pyrazine | C_14_H_22_N_2_O_2_ | 347922 | ND | ND | ND | 295993 | 139013 | 479637 | ND |
| 17:38 | 166.1102 | 5-Hydroxy-2,2,6,6-tetramethyl-4-cyclohexene-1,3-dione | C_10_H_14_O_3_ | ND | ND | ND | ND | ND | 150077 | ND | ND |
| 17:43 | 123.0807 | Glutaric acid, di(3-heptyl) ester | C_19_H_36_O_4_ | ND | 32302 | ND | ND | ND | 41043 | ND | ND |
| 17:43 | 205.0868 | Glutaric acid, 2-methylphenyl octadecyl ester | C_30_H_50_O_4_ | ND | 29422 | ND | ND | ND | ND | ND | ND |
| 17:43 | 221.1168 | Glutaric acid, hept-2-yl 3-octyl ester | C_20_H_38_O_4_ | ND | ND | ND | ND | 207877 | ND | 327036 | ND |
| 17:44 | 287.8767 | 1,3-Dioxolane, 4,4,5-trimethyl-2-pentadecyl- | C_21_H_42_O_2_ | 92866 | ND | ND | 629151 | ND | ND | ND | ND |
| 17:44 | 252.1361 | Glutaric acid, 2-chloro-6-fluorophenyl decyl ester | C_21_H_30_ClFO_4_ | ND | ND | 781530 | ND | ND | ND | ND | ND |
| 17:44 | 179.1440 | Glutaric acid, di(but-3-en-2-yl) ester | C_13_H_20_O_4_ | 125743 | ND | 807162 | ND | ND | ND | ND | ND |
| 17:46 | 227.2009 | n-Hexadecanoic acid | C_16_H_32_O_2_ | ND | ND | ND | ND | ND | 365620 | 6691449 | ND |
| 17:47 | 223.0968 | Dibutyl phthalate | C_16_H_22_O_4_ | 1031636 | 163645 | 6770864 | 98696 | ND | 62921 | ND | ND |
| 17:47 | 223.0972 | 1,2-Benzenedicarboxylic acid, butyl 2-ethylhexyl ester | C_20_H_30_O_4_ | ND | 252338 | ND | ND | ND | ND | ND | ND |
| 17:47 | 223.0964 | Phthalic acid, 2,7-dimethyloct-7-en-5-yn-4-yl nonyl ester | C_27_H_38_O_4_ | ND | ND | ND | ND | ND | 321080 | ND | ND |
| 17:47 | 224.0999 | Phthalic acid, 8-chlorooctyl heptyl ester | C_23_H_35_ClO_4_ | ND | ND | ND | ND | 2745947 | ND | ND | ND |
| 17:47 | 225.1028 | Phthalic acid, 8-chlorooctyl nonyl ester | C_25_H_39_ClO_4_ | 1376222 | ND | ND | ND | ND | ND | 1501541 | ND |
| 17:48 | 252.9111 | Phthalic acid, 2-chloropropyl heptyl ester | C_18_H_25_ClO_4_ | ND | ND | ND | ND | 3192901 | ND | ND | ND |
| 17:48 | 236.1047 | Phthalic acid, pentyl tridec-2-yn-1-yl ester | C_26_H_38_O_4_ | ND | ND | 7508833 | ND | ND | ND | ND | ND |
| 17:48 | 224.0997 | Phthalic acid, 8-chlorooctyl decyl ester | C_26_H_41_ClO_4_ | ND | ND | ND | ND | ND | ND | 1083793 | ND |
| 17:48 | 279.1555 | Phthalic acid, butyl hexyl ester | C_18_H_26_O_4_ | ND | ND | ND | 26341641 | ND | ND | ND | ND |
| 17:49 | 252.2812 | Nonane, 2,2,4,4,6,8,8-heptamethyl- | C_16_H_34_ | ND | ND | 1006922 | ND | ND | ND | ND | ND |
| 17:49 | 297.6906 | Tetradecane, 1-iodo- | C_14_H_29_I | ND | ND | 1177244 | ND | ND | ND | ND | ND |
| 17:51 | 200.1738 | Laurinlactam, N-(diethylboryl)- | C_12_H_24_O_2_ | ND | ND | ND | ND | ND | ND | ND | ND |
| 17:51 | 154.0657 | 1,2,3,4,5-Cyclopentanepentol | C_5_H_10_O_5_ | ND | ND | ND | ND | 1152556 | ND | ND | ND |
| 17:51 | 210.1153 | 3-[1-(4-Cyano-1,2,3,4-tetrahydronaphthyl)]propanenitrile | C_14_H_14_N_2_ | ND | ND | 161146 | ND | ND | ND | ND | ND |
| 17:56 | 126.1400 | Hexahydronerolidol | C_15_H_32_O | 39634 | ND | ND | ND | ND | ND | ND | ND |
| 17:56 | 208.1221 | 8-Methoxy-5,5,8-trimethyl-3-nonen-2-one | C_13_H_24_O_2_ | 78237 | ND | ND | ND | ND | ND | ND | ND |
| 17:57 | 154.0738 | Silane, (1-cyclopenten-1-ylmethyl)trimethyl- | C_9_H_18_Si | ND | ND | ND | ND | ND | ND | 292148 | ND |
| 18:01 | 255.2317 | Heptadecanoic acid, methyl ester | C_18_H_36_O_2_ | ND | ND | ND | 186005 | ND | ND | ND | ND |
| 18:02 | 221.2264 | Isoquinoline, 1-[(3,5-dihydroxy)benzyl]-1,2,3,4-tetrahydro-6-methoxy- | C_17_H_19_NO_3_ | ND | ND | ND | 42106 | ND | ND | ND | ND |
| 18:02 | 172.0882 | 1,2,3,4-Tetrahydroisoquinoline, 1-[3-hydroxybenzyl]-6-methoxy- | C_17_H_19_NO_2_ | ND | ND | ND | 24786 | ND | ND | ND | ND |
| 18:02 | 210.1161 | Quinolinium, 1-ethyl-, iodide | C_11_H_12_IN | ND | ND | 223952 | ND | ND | ND | ND | ND |
| 18:03 | 241.2162 | Octadecanoic acid, ethyl ester | C_20_H_40_O_2_ | 296681 | ND | 1286003 | ND | ND | ND | ND | 197612 |
| 18:03 | 172.0888 | 1-[6,8-Dichloro-2-phenyl-4-quinolyl]hexahydro-3H-oxazolo[3,4-a]pyridine | C_22_H_20_Cl_2_N_2_O | ND | ND | 607805 | ND | ND | ND | ND | ND |
| 18:03 | 284.2711 | Eicosanoic acid, ethyl ester | C_22_H_44_O_2_ | 361956 | ND | ND | ND | ND | ND | ND | ND |
| 18:03 | 284.2705 | Docosanoic acid, ethyl ester | C_24_H_48_O_2_ | ND | ND | 754931 | ND | ND | ND | ND | ND |
| 18:03 | 199.1696 | Decanoic acid, ethyl ester | C_12_H_24_O_2_ | ND | ND | ND | 534776 | ND | ND | ND | ND |
| 18:04 | 162.1279 | 1-Methyl -3,4-dihydro-2H-quinolin-7-amine | C_10_H_14_N_2_ | ND | ND | ND | 2997 | ND | ND | ND | ND |
| 18:05 | 240.0973 | Metolachlor | C_15_H_22_ClNO_2_ | ND | ND | 69359 | 50126 | ND | ND | 35682 | ND |
| 18:06 | 286.8744 | Tetracosane | C_24_H_50_ | 2570347 | ND | 5288732 | 4256419 | 2775995 | 1154621 | ND | ND |
| 18:10 | 315.9547 | Chlorpyrifos | C_9_H_11_Cl_3_NO_3_PS | ND | ND | 48958 | 78017 | ND | ND | ND | ND |
| 18:21 | 257.2478 | Palmitic acid, isopropyl ester | C_19_H_38_O_2_ | ND | ND | 163434 | 128063 | ND | ND | ND | ND |
| 18:22 | 257.2468 | Deltyl | C_19_H_38_O_2_ | ND | ND | ND | 68415 | ND | ND | ND | ND |
| 18:31 | 275.2368 | Cyclohexanol, 1-[3-di(3-methylbuthyl)aminoprop-1-ynyl | C_19_H_35_NO | ND | ND | ND | 276656 | ND | ND | ND | ND |
| 18:31 | 258.2329 | 1-Cyclopropyl-4-(4-methoxycarbonyl-phenyl)-1,4-dihydro-pyridine-3,5-dicarboxylic acid dimethyl ester | C_20_H_21_NO_6_ | ND | ND | ND | 268904 | 69591 | ND | ND | ND |
| 18:41 | 253.2878 | 3,3-Dimethylnonadecane | C_21_H_44_ | ND | ND | ND | 498920 | ND | ND | ND | ND |
| 18:52 | 214.1882 | 4-Heptanone, 5,5-diethyl-2,2,3,3-tetramethyl- | C_15_H_30_O | ND | ND | ND | ND | 142208 | ND | ND | ND |
| 18:57 | 136.1243 | Methylphenidate | C_14_H_19_NO_2_ | ND | ND | ND | ND | ND | ND | ND | ND |
| 18:58 | 145.0646 | 1-Pyrrolidineacetic acid, 2-methyl-5-oxo-, ethyl ester | C_9_H_15_NO_3_ | ND | ND | ND | ND | ND | ND | ND | ND |
| 18:58 | 285.8761 | Behenic alcohol | C_22_H_46_O | ND | ND | ND | ND | 2714258 | ND | ND | ND |
| 18:59 | 165.1269 | Octadecanoic acid, 2-oxo-, methyl ester | C_19_H_36_O_3_ | ND | ND | ND | 448464 | ND | ND | ND | ND |
| 18:59 | 272.2497 | Succinic acid, 2-methylpent-3-yl tetrahydrofurfuryl ester | C_15_H_26_O_5_ | ND | ND | ND | 478789 | ND | ND | ND | ND |
| 19:00 | 201.0691 | 2,3-Dimethyl-undec-1-en-3-ol | C_13_H_26_O | ND | ND | ND | ND | ND | ND | 1322950 | ND |
| 19:00 | 255.2321 | 2H-Tetrahydropyran, 2-[(1-ethenyl-1,5-dimethylhex-4-enyl)oxy]- | C_15_H_26_O_2_ | ND | ND | ND | ND | ND | ND | 1004350 | ND |
| 19:03 | 236.1565 | cyclohexane, [1,1'-biphenyl]-4-yl- | C_18_H_20_ | ND | ND | 49890 | ND | ND | ND | ND | ND |
| 19:04 | 236.1559 | 4-cyclohexylbiphenyl | C_18_H_20_ | ND | ND | ND | 194769 | ND | ND | ND | ND |
| 19:03 | 236.1560 | cyclohexane, [1,1'-biphenyl]-4-yl- ; 4-cyclohexylbiphenyl | C_18_H_20_ | ND | ND | ND | 202343 | ND | ND | ND | ND |
| 19:07 | 244.1091 | 4H-Thiazolo[5,4-b]indole, 4,7-diethyl-2-methyl- | C_14_H_16_N_2_S | ND | ND | ND | 46112 | ND | ND | ND | ND |
| 19:08 | 266.2564 | 11-Octadecenoic acid, methyl ester | C_19_H_36_O_2_ | 252742 | ND | 2533197 | ND | ND | ND | ND | ND |
| 19:08 | 266.2565 | trans-13-Octadecenoic acid, methyl ester | C_19_H_36_O_2_ | 408320 | ND | ND | ND | DN | ND | ND | ND |
| 19:08 | 267.2597 | (Z)-Methyl hexadec-11-enoate | C_17_H_32_O_2_ | ND | ND | 2442730 | ND | ND | ND | ND | ND |
| 19:09 | 198.1055 | 2,6-Pyridinedicarboxaldehyde, 4-hydroxy-, bis[methyl(2-pyridyl)hydrazone] | C_19_H_19_N_7_O | 290502 | ND | ND | ND | ND | ND | ND | ND |
| 19:10 | 152.1556 | Isovaleric acid, 3-methylbutyl-2 ester | C_10_H_20_O_2_ | ND | ND | ND | 680811 | ND | ND | ND | ND |
| 19:10 | 295.2626 | Succinic acid, 3-methylbut-2-yl 2-methylpent-3-yl ester | C_15_H_28_O_4_ | ND | ND | ND | 719600 | ND | ND | ND | ND |
| 19:10 | 59.0494 | Methylamine, N,N-dimethyl- | C_3_H_9_N | ND | ND | 325216 | ND | ND | ND | ND | ND |
| 19:10 | 295.2640 | Sulfurous acid, hexyl nonyl ester | C_15_H_32_O_3_S | ND | ND | ND | ND | DN | ND | 1490909 | ND |
| 19:11 | 112.1248 | Butyronitrile, 4-(dimethylamino)- | C_6_H_12_N_2_ | ND | ND | 330312 | ND | ND | ND | ND | ND |
| 19:13 | 192.1874 | 2(3H)-Furanone, 5-heptyldihydro- | C_11_H_20_O_2_ | ND | ND | ND | ND | ND | 269853 | ND | ND |
| 19:13 | 192.1866 | 2(3H)-Furanone, 5-dodecyldihydro- | C_16_H_30_O_2_ | ND | ND | ND | ND | ND | 259261 | ND | ND |
| 19:14 | 139.8589 | 2(3H)-Furanone, 5-butyldihydro- | C_8_H_14_O_2_ | ND | ND | 402089 | ND | ND | ND | 199266 | ND |
| 19:14 | 164.1541 | 1-Benzoxepin-2(3H)-one, octahydro- | C_10_H_16_O_2_ | ND | ND | ND | 501367 | ND | 113802 | ND | ND |
| 19:14 | 264.2443 | Benzamide, N-(2-piperidinylmethyl)-2,5-bis(2,2,2-trifluoroethoxy)- | C_17_H_20_F_6_N_2_O_3_ | ND | ND | ND | ND | ND | ND | 652762 | ND |
| 19:14 | 179.1796 | 2-n-Heptylcyclopentanone | C_12_H_22_O | ND | ND | ND | ND | ND | ND | 588754 | ND |
| 19:16 | 179.1796 | Phytol | C_20_H_40_O | ND | ND | ND | ND | ND | ND | 902142 | ND |
| 19:22 | 298.2873 | Methyl stearate | C_19_H_38_O_2_ | 825634,5 | 191139 | ND | 4360100 | 1892907 | ND | ND | ND |
| 19:22 | 270.2513 | Pentadecanoic acid, 14-methyl-, methyl ester | C_17_H_34_O_2_ | ND | ND | 3038135 | ND | ND | ND | 182375 | ND |
| 19:22 | 298.2868 | Hexadecanoic acid, 15-methyl-, methyl ester | C_18_H_36_O_2_ | ND | ND | 3245745 | ND | ND | 374502 | ND | ND |
| 19:24 | 430.0890 | Heptasiloxane, hexadecamethyl- | C_16_H_48_O_6_Si_7_ | ND | 144274 | ND | ND | ND | ND | ND | ND |
| 19:24 | 356.0701 | 3,4-Dihydroxyphenylglycol, 4TMS derivative | C_20_H_42_O_4_Si_4_ | ND | ND | ND | 40741 | ND | ND | ND | ND |
| 19:28 | 168.1874 | 2-Cyclohexen-1-ol, 4-amino-5,6-dimethoxy- | C_8_H_15_NO_3_ | ND | ND | 371888 | ND | ND | ND | ND | ND |
| 19:28 | 197.2262 | Fumaric acid, isobutyl tetrahydrofurfuryl ester | C_13_H_20_O_5_ | ND | ND | 520547 | 1129680 | ND | ND | ND | ND |
| 19:30 | 224.2504 | Decanamide, N-(2-hydroxyethyl)- | C_12_H_25_NO_2_ | ND | ND | 158794 | 84680 | 131193 | ND | 141378 | ND |
| 19:31 | 254.2028 | Dodecanamide, N-(2-hydroxyethyl)- | C_14_H_29_NO_2_ | ND | ND | 158794 | ND | ND | ND | ND | ND |
| 19:32 | 187.1123 | 2H-Pyran-2-one, 6-hexyltetrahydro- | C_11_H_20_O_2_ | ND | ND | 127031 | ND | ND | ND | ND | D |
| 19:33 | 149.1326 | cyclopentanone, 2-octadecyl- | C_23_H_44_O | ND | ND | ND | ND | ND | 164330 | 1236016 | ND |
| 19:33 | 202.0781 | 1-Hexadecyn-3-ol, 3,7,11,15-tetramethyl- | C_20_H_38_O | ND | ND | ND | 1215883 | 317138 | ND | ND | ND |
| 19:34 | 203.0817 | cyclopentanone, 2-decyl- | C_15_H_28_O | ND | ND | ND | ND | ND | ND | 1438993 | ND |
| 19:42 | 264.2440 | 9,12-Octadecadienoic acid, methyl ester, (E,E)- | C_19_H_34_O_2_ | 286963,5 | 727671 | 1698384 | ND | ND | 328880 | ND | ND |
| 19:45 | 266.2560 | E-11-Hexadecenoic acid, ethyl ester | C_18_H_34_O_2_ | ND | ND | ND | ND | ND | ND | ND | 209308 |
| 19:46 | 230.1089 | Methyl 2-hydroxy-octadeca-9,12,15-trienoate | C_19_H_32_O_3_ | 198986 | ND | ND | ND | ND | ND | ND | ND |
| 19:47 | 227.2008 | Myristic acid | C_14_H_28_O_2_ | ND | ND | ND | ND | ND | ND | 681479 | ND |
| 19:47 | 228.2042 | Tetradecanoic acid | C_14_H_28_O_2_ | ND | ND | ND | ND | ND | ND | 918665 | ND |
| 19:50 | 178.0981 | 3-Aza-2-oxabicyclo[2.2.2]oct-5-ene, 3-acetyl-7-endo-dimethylamino-8-exo-acetoxy- | C_12_H_18_N_2_O_4_ | ND | ND | ND | ND | ND | ND | 94180 | ND |
| 19:54 | 223.0632 | Palmitamide | C_16_H_33_NO | 86807 | ND | ND | ND | ND | ND | ND | ND |
| 19:55 | 150.1034 | N-[3,3'-Dimethoxy-4'-(2-piperidin-1-yl-acetylamino)-biphenyl-4-yl]-2-piperidin-1- yl-acetamide | C_28_H_38_N_4_O_4_ | ND | ND | ND | ND | 27303 | ND | ND | ND |
| 19:55 | 287.8787 | Fenpipramide | C_21_H_26_N_2_O | ND | ND | ND | ND | 24630 | ND | ND | ND |
| 19:56 | 146.0723 | 6,8-Dichlorooctanamide | C_8_H_15_Cl_2_NO | ND | ND | 40098 | ND | ND | ND | ND | ND |
| 19:57 | 128.1070 | Nonanamide | C_9_H_19_NO | ND | ND | ND | ND | 125869 | ND | 131617 | ND |
| 19:58 | 287.8787 | Dodecane, 1,2-dibromo- | C_12_H_24_Br_2_ | ND | ND | ND | ND | 1534219 | ND | ND | ND |
| 19:58 | 279.2682 | 1-Bromo-3-(2-bromoethyl)-nonane | C_11_H_22_Br_2_ | ND | ND | ND | ND | 1727597 | ND | 1059173 | ND |
| 19:58 | 180.1862 | 4,4,8-Trimethyl-non-5-enal | C_12_H_22_O | ND | ND | ND | 651632 | ND | ND | ND | ND |
| 19:58 | 279.2673 | Ethyl 4,8,12-trimethyl-tridecanoate | C_18_H_36_O_2_ | ND | ND | ND | 444741 | ND | ND | ND | ND |
| 19:59 | 269.2476 | Octadecanoic acid, 17-methyl-, methyl ester | C_20_H_40_O_2_ | 202833 | ND | ND | ND | ND | ND | ND | ND |
| 19:59 | 279.2682 | Hexadecanoic acid, (3-bromoprop-2-ynyl) ester | C_19_H_33_BrO_2_ | ND | ND | ND | ND | ND | ND | 1222426 | ND |
| 20:07 | 167.0856 | Hexadecyl propyl ether | C_19_H_40_O | ND | ND | ND | 93092 | ND | ND | ND | ND |
| 20:14 | 213.1301 | 2-Isobutoxy-N-[2-(2-methyl-1H-indol-3-yl)-ethyl]-benzamide | C_22_H_26_N_2_O_2_ | ND | ND | ND | ND | 239990 | ND | ND | ND |
| 20:14 | 172.0905 | 2-Pentenoic acid, trimethylsilyl ester | C_8_H_16_O_2_Si | ND | ND | ND | ND | ND | 355291 | ND | ND |
| 20:15 | 223.1332 | cis-3-Hexen-1-ol, tert-butyldimethylsilyl ether | C_12_H_26_OSi | ND | ND | ND | ND | ND | ND | 177232 | ND |
| 20:15 | 223.1323 | Triisopropylsilyloxycyclobutane | C_13_H_28_OSi | ND | ND | ND | ND | ND | ND | 515652 | ND |
| 20:33 | 202.0782 | Propylphosphonic acid | C_9_H_20_FO_2_P | ND | ND | 29384 | ND | ND | ND | ND | ND |
| 20:34 | 180.1137 | L-5-Propylthiomethylhydantoin | C_7_H_12_N_2_O_2_S | ND | ND | ND | ND | ND | ND | 65028 | ND |
| 20:34 | 154.1710 | 8-Azabicyclo[3.2.1]octane, 3-chloro-8-methyl- | C_8_H_14_ClN | ND | ND | ND | ND | 36806 | 89906 | ND | ND |
| 20:35 | 142.1226 | N,N-Dimethylhexanamide | C_8_H_17_NO | ND | ND | ND | ND | 126375 | ND | ND | ND |
| 20:35 | 172.0891 | Octanamide, N,N-dimethyl- | C_10_H_21_NO | ND | ND | ND | ND | ND | ND | 104187 | ND |
| 20:35 | 162.1032 | 3-Cyclopentylpropionamide, N,N-dimethyl- | C_10_H_19_NO | ND | ND | ND | ND | ND | ND | 98340 | ND |
| 20:38 | 225.1150 | Undec-10-ynoic acid, dodecyl ester | C_23_H_42_O_2_ | ND | ND | ND | ND | ND | ND | 473268 | ND |
| 20:41 | 155.0734 | Succinic acid, 2,4,6-trichlorophenyl phenethyl ester | C_18_H_15_Cl_3_O_4_ | ND | ND | 334075 | ND | ND | ND | ND | ND |
| 20:42 | 288.2695 | Benzene, (1-methylhexadecyl)- | C_23_H_40_ | ND | ND | ND | ND | 251659 | ND | ND | ND |
| 20:46 | 209.0872 | Pyrrolo[1,2-a]pyrazine-3-propanamide, 2,3,6,7,8,8a-hexahydro-1,4-dioxo- | C_10_H_15_N_3_O_3_ | 413283 | ND | ND | ND | ND | ND | ND | ND |
| 20:46 | 132.0929 | Carbonic acid, 2-dimethylaminoethyl methyl ester | C_6_H_13_NO_3_ | 71727 | ND | ND | ND | ND | ND | ND | ND |
| 20:47 | 238.1452 | Fumaric acid, 2-dimethylaminoethyl hexadecyl ester | C_24_H_45_NO_4_ | 129462 | ND | ND | ND | ND | ND | ND | ND |
| 20:47 | 261.1497 | Dibenzothiophen-1-one, 1,2,3,4,6,7,8,9-octahydro-, o-(2-dimethylaminoethyl)oxime | C_16_H_24_N_2_OS | ND | ND | 92574 | ND | ND | ND | ND | ND |
| 20:47 | 204.0930 | Octanoic acid, 2-dimethylaminoethyl ester | C_12_H2_5_NO_2_ | ND | ND | ND | 109727 | ND | ND | ND | ND |
| 20:48 | 180.0918 | Fumaric acid, 2-dimethylaminoethyl dodecyl ester | C_20_H_37_NO_4_ | ND | ND | ND | ND | 91487 | ND | ND | ND |
| 20:48 | 283.0481 | 3-Cyclopentylpropionic acid, 2-dimethylaminoethyl ester | C_12_H_23_NO_2_ | ND | ND | ND | ND | ND | ND | 310058 | ND |
| 20:49 | 210.2334 | 16,28-Secosolanid-5-en-3-ol, (3á)- | C_27_H_45_NO | ND | ND | ND | ND | 27408 | ND | ND | ND |
| 20:50 | 208.2180 | 1-Docosene | C_22_H_44_ | ND | ND | ND | ND | 1235509 | ND | ND | ND |
| 20:50 | 287.8782 | n-Tetracosanol-1 | C_24_H_50_O | ND | ND | ND | ND | 1453509 | ND | ND | ND |
| 20:54 | 202.0987 | Acrylic acid, tridecyl ester | C_16_H3_0_O_2_ | 289487 | ND | ND | ND | ND | ND | ND | ND |
| 20:54 | 211.1301 | Tritetracontane | C_43_H_88_ | 622967 | ND | ND | ND | ND | ND | ND | ND |
| 20:55 | 222.2336 | 4-(2-Chloro-phenyl)-1-methyl-1,4-dihydro-pyridine-3,5-dicarboxylic acid dimethyl ester | C_16_H_16_ClNO_4_ | ND | ND | 41942 | ND | ND | ND | ND | ND |
| 20:56 | 273.1514 | benzenamine, 2,6-dimethyl-N,N-diphenyl- | C_20_H_19_N | ND | ND | 33574 | ND | ND | ND | ND | ND |
| 21:04 | 248.2032 | Tetrahydroverazine B | C_27_H_47_NO | 42463 | ND | ND | ND | ND | ND | ND | ND |
| 21:05 | 262.2175 | 16,28-Secosolanidan-3-ol, (3á,5à)- | C_27_H_47_NO | 101344 | ND | ND | ND | ND | ND | ND | ND |
| 21:05 | 276.2339 | Palmidrol | C_18_H_37_NO_2_ | ND | ND | 412763 | ND | ND | ND | ND | ND |
| 21:06 | 192.1391 | Octanamide, N-(2-hydroxyethyl)- | C_10_H2_1_NO_2_ | ND | ND | 480044 | ND | ND | ND | ND | ND |
| 21:07 | 264.2324 | Octadecanamide, N-(2-hydroxyethyl)- | C_20_H_41_NO_2_ | 79816 | ND | ND | ND | 159659 | ND | 513263 | ND |
| 21:15 | 226.1098 | 9H-Thioxanthen-9-one, 2-methyl- | C_14_H_10_OS | 139719 | ND | ND | ND | ND | ND | ND | ND |
| 21:23 | 200.1159 | 4,5-Dihydro-á,á,4,4-tetramethyl-1H-pyrazole-1-propanol | C_10_H_20_N_2_O | ND | ND | ND | ND | ND | ND | 137003 | ND |
| 21:23 | 164.1197 | Thiophene-2-carboxylic acid, 2,6-dimethyl-4-(2,2,2-trifluoro-1-hydroxy-1-trifluoromethylethyl)phenyl ester | C_16_H_12_F_6_O_3_S | ND | ND | ND | ND | 151883 | ND | ND | ND |
| 21:23 | 154.0864 | 2-Thiophenecarboxylic acid, 1-naphthyl ester | C_15_H_10_O_2_S | ND | ND | ND | ND | 131170 | ND | ND | ND |
| 21:26 | 168.1490 | 1H-imidazole-2-methanol, 1-pentyl- | C_9_H_16_N_2_O | ND | ND | ND | 177561 | ND | ND | ND | ND |
| 21:26 | 262.0821 | trans-Oleic acid | C_18_H_34_O_2_ | ND | ND | ND | 267765 | DN | ND | ND | ND |
| 21:27 | 137.0963 | 2-Octen-4-one, 2-methoxy- | C_9_H_16_O_2_ | 64111 | ND | 429846 | 263110 | ND | ND | ND | ND |
| 21:27 | 315.0328 | Ergotaman-3',6',18-trione, 9,10-dihydro-12'-hydroxy-2'-methyl-5'-(phenylmethyl)-, (5'à,10à)- | C_33_H_37_N_5_O_5_ | 2669515 | ND | ND | ND | ND | ND | ND | ND |
| 21:27 | 166.1352 | Tetrahydrofuran-2-one, 3-[2-pentenyl]-4-methyl- | C_10_H_16_O_2_ | ND | ND | ND | 420376 | ND | ND | ND | ND |
| 21:27 | 246.1271 | 4,8,12,16-Tetramethylheptadecan-4-olide | C_21_H_40_O_2_ | 309026 | ND | 486847 | 724570 | 858767 | 135574 | 1270848 | ND |
| 21:28 | 192.0937 | 2-Propanol, 3-(1-piperidinyl)-1-propoxy- | C_11_H_23_NO_2_ | ND | ND | 27433 | ND | ND | ND | ND | ND |
| 21:29 | 240.1828 | Phthalic acid, isopentyl 3-methylphenyl ester | C_20_H_22_O_4_ | ND | ND | ND | 37160 | ND | ND | ND | ND |
| 21:29 | 240.1838 | 4-(4-Oxo-1,2,3,4,6,7,12,12b-octahydropyrido[2,1-a]-á-carbolin-12b-yl)butanoic acid | C_19_H_22_N_2_O_3_ | ND | ND | 42677 | ND | ND | ND | ND | ND |
| 21:39. | 284.1522 | Ethyl 2,5-dimethyl-9-oxo-1H,6H,9H-pyrrolo[2,3-f]quinoline-7-carboxylate | C_16_H_16_N_2_O_3_ | ND | ND | 68461 | ND | ND | ND | ND | ND |
| 21:41 | 273.2571 | (1S,7'S)-2,2-Dimethyl-7'-nitrohexahydrospiro[cyclopentane-1,1'-pyrrolizine] | C_13_H_22_N_2_O_2_ | ND | ND | 75025 | ND | ND | ND | ND | ND |
| 21:41 | 167.1794 | Cyclohexane, decyl- | C_16_H_32_ | ND | ND | ND | ND | 155802 | ND | ND | ND |
| 21:41 | 166.0774 | n-Heptadecanol-1 | C_17_H_36_O | ND | ND | ND | ND | 148882 | ND | ND | ND |
| 21:43 | 154.0771 | Methyl-6-deoxy-6-fluoro-2,3,4-tri-O-methylád-galactopyranoside | C_10_H_19_FO_5_ | ND | ND | 49688 | ND | ND | ND | ND | ND |
| 21:43 | 228.1258 | Dodecanoic acid, ethyl ester | C14H28O2 | 129866,5 | ND | 553592 | 406075 | ND | ND | 133988 | ND |
| 21:43 | 198.1438 | Decanoic acid, 2,4,6-trimethyl-, methyl ester | C_14_H_28_O_2_ | ND | ND | ND | 47617 | ND | ND | 156602 | ND |
| 21:43 | 157.1226 | Vinyldimethyl(N-methylcarbamoyloxymethyl)silane | C_7_H_15_NO_2_Si | ND | ND | 20151 | 61267 | ND | ND | ND | ND |
| 21:45 | 244.1566 | Hentriacontane | C_31_H_64_ | 878607 | ND | ND | ND | ND | ND | ND | ND |
| 21:45 | 211.2422 | 1,1,1-Trichloro-4,4-dimethyl-4-vinyldisilethylene | C_6_H_13_Cl_3_Si_2_ | ND | ND | ND | 841031 | ND | ND | ND | ND |
| 21:45 | 125.1326 | 4-Methyl-1,6-heptadien-4-ol | C_8_H_14_O | ND | ND | ND | 708440 | ND | ND | ND | ND |
| 21:46 | 140.1561 | 2-Pentanone, 5-(1,2-propadienyloxy)- | C_8_H_12_O_2_ | ND | ND | 1969107 | ND | 251262 | ND | ND | ND |
| 21:46 | 112.1249 | 1-Heptene, 5-methyl- | C_8_H_16_ | ND | 92429 | ND | ND | ND | ND | ND | ND |
| 21:46 | 147.0660 | Hexanedioic acid, mono(2-ethylhexyl)ester | C_14_H_26_O_4_ | 390998 | 61810 | ND | ND | ND | 154528 | ND | ND |
| 21:46 | 242.1837 | Adipic acid, 2-ethylhexyl isohexyl ester | C_20_H_38_O_4_ | 710648 | ND | ND | ND | ND | 140879 | ND | ND |
| 21:47 | 259.1905 | Hexanedioic acid, bis(2-ethylhexyl) ester | C_22_H_42_O_4_ | ND | ND | 2551767 | 2544762 | 1546986 | 263824 | 1267335 | ND |
| 21:56 | 199.0736 | Fumaric acid, 4-chlorophenyl propyl ester | C_13_H_13_ClO_4_ | ND | ND | ND | ND | ND | ND | 39347 | ND |
| 21:57 | 273.1484 | Piperonyl butoxide | C_19_H_30_O_5_ | ND | ND | 36400 | 30626 | ND | ND | ND | ND |
| 22:00 | 234.1036 | 1-(2-Carboxyethyl)-2H,3H,4H,9H-pyrido[3,4-b]indole-1-carboxylic acid | C_15_H_16_N_2_O_4_ | 293338 | ND | ND | ND | ND | ND | ND | ND |
| 22:03 | 340.2400 | Phenol, 2,2'-methylenebis[6-(1,1-dimethylethyl)-4-methyl- | C_23_H_32_O_2_ | ND | ND | 37844 | ND | ND | ND | ND | ND |
| 22:13 | 267.1585 | 2,6-Difluorobenzoic acid, 2-chlorophenyl ester | C_13_H_7_ClF_2_O_2_ | ND | ND | ND | ND | ND | ND | 61337 | ND |
| 22:13 | 267.1592 | 2,4-Difluorobenzoic acid, 2,4-dichloronaphthyl-1 ester | C_17_H_8_Cl_2_F_2_O_2_ | ND | ND | ND | ND | ND | ND | 66823 | ND |
| 22:14 | 124.9963 | 1-(2-Dimethylamino-ethyl)-3,6-dimethyl-1H-pyrimidine-2,4-dione | C_10_H_17_N_3_O_2_ | 172928 | ND | ND | ND | ND | ND | ND | ND |
| 22:14 | 138.1033 | Bis(2-(Dimethylamino)ethyl) ether | C_8_H_20_N_2_O | ND | ND | 257998 | ND | ND | ND | ND | ND |
| 22:14 | 235.1235 | Carbonic acid, 2-dimethylaminoethyl 2-methoxyethyl ester | C_8_H_17_NO_4_ | 269110 | ND | ND | ND | ND | ND | ND | ND |
| 22:15 | 72.0808 | 4-Quinolinecarboxamide, N-[2-(dimethylamino)ethyl]-2-(2-pyridinyl)- | C_19_H_20_N_4_O | ND | ND | ND | 108776 | ND | ND | ND | ND |
| 22:15 | 113.0960 | Carbonic acid, 2-dimethylaminoethyl ethyl ester | C_7_H_15_NO_3_ | ND | ND | 173327 | 93744 | ND | ND | ND | ND |
| 22:16 | 144.0477 | Bis(2-(Dimethylamino)ethyl) ether | C_8_H_20_N_2_O | 110530 | ND | ND | ND | ND | ND | ND | ND |
| 22:19 | 319.1023 | Phosphoramidic acid, (1-methylethyl)-, ethyl 3-methyl-4-(methylsulfinyl)phenyl ester | C1_3_H_22_NO_4_PS | ND | ND | 61632 | ND | ND | ND | 79403 | ND |
| 22:21 | 305.0793 | Nemacur sulfoxide | C_13_H_22_NO_4_PS | ND | ND | ND | ND | ND | ND | 88020 | ND |
| 22:21 | 275.1637 | Succinic acid, diamide, N,N'-diethyl-N,N'-diphenyl- | C_20_H_24_N_2_O_2_ | 57815 | ND | ND | ND | ND | ND | ND | ND |
| 22:25 | 371.2626 | 3,7,11,15,18-Pentaoxa-2,19-disilaeicosane, 2,2,19,19-tetramethyl- | C_17_H_40_O_5_Si_2_ | 76650 | ND | ND | ND | 155078 | ND | ND | ND |
| 22:25 | 315.0345 | Glycyl-L-tryptophylglycine | C_15_H_18_N_4_O_4_ | 28452 | ND | ND | ND | ND | ND | 1736803 | ND |
| 22:26 | 213.0909 | 1-hydroxy-1,2,3,4-tetrahydronaphthalene trifluoroacetate ester | C_12_H_11_F_3_O_2_ | ND | ND | ND | ND | ND | ND | 187351 | ND |
| 22:26 | 239.2373 | 1,2,3,4-Tetrahydro-3-(phenylacetamido)quinoline | C_17_H_18_N_2_O | ND | ND | ND | ND | ND | ND | 161706 | ND |
| 22:28 | 202.0791 | Carbonic acid, 2-dimethylaminoethyl isobutyl ester | C_9_H1_9_NO_3_ | 100668 | ND | ND | ND | ND | ND | ND | ND |
| 22:31 | 125.1327 | Pyridine, 1-acetyl-1,2,3,4-tetrahydro- | C_7_H_11_NO | ND | ND | ND | 139368 | ND | ND | ND | ND |
| 22:33 | 314.2578 | 1H-Indene, 1-hexadecyl-2,3-dihydro- | C_25_H_42_ | ND | ND | ND | ND | ND | 394456 | ND | ND |
| 22:33 | 259.1368 | Octadecane | C_18_H_38_ | ND | ND | 1094533 | 1168453 | 2623371 | ND | ND | ND |
| 22:33 | 314.2581 | Ethyl 3-hydroxyicosanoate ; Eicosanoicacid, 3-hydroxy-, ethyl ester | C_22_H_44_O_3_ | ND | ND | ND | ND | ND | 252163 | ND | ND |
| 22:33 | 203.0738 | Tetradecane ; n-Tetradecane | C_14_H_30_ | ND | ND | 945639 | ND | ND | ND | ND | ND |
| 22:34 | 343.2299 | 1-n-Hexadecylindan | C_25_H_42_ | ND | ND | ND | ND | ND | ND | 2041696 | ND |
| 22:35 | 119.0336 | Isoindoline | C_8_H_9_N | ND | ND | ND | ND | ND | ND | ND | ND |
| 22:41 | 257.2486 | Palmitic acid á-monoglyceride | C_19_H_38_O_4_ | ND | ND | ND | 202558 | ND | ND | ND | ND |
| 22:42 | 299.2582 | Glycerol 1-palmitate | C_19_H_38_O_4_ | ND | ND | ND | ND | ND | ND | 583933 | ND |
| 22:48 | 219.0931 | Tridecanoic acid, methyl ester | C_14_H_28_O_2_ | 140149 | ND | 233636 | 518090 | 229959 | ND | 1792989 | ND |
| 22:48 | 194.2027 | Decanoic acid, methyl ester | C_11_H_22_O_2_ | ND | ND | 154841 | ND | ND | ND | 159811 | ND |
| 22:50 | 208.2184 | 2,2,4,4,5,5,7,7-Octamethyloctane | C_16_H_34_ | ND | ND | 219713 | ND | ND | ND | ND | ND |
| 22:54 | 284.1043 | Homopterocarpin | C_17_H_16_O_4_ | ND | ND | ND | 814585 | ND | ND | ND | ND |
| 23:00 | 279.1606 | Diisooctyl phthalate | C_24_H_38_O_4_ | 198708 | ND | ND | ND | ND | ND | ND | ND |
| 23:01 | 357.0683 | Phthalic acid, di(oct-3-yl) ester | C_24_H_38_O_4_ | ND | ND | ND | ND | ND | 92479 | ND | ND |
| 23:01 | 280.1626 | Dicyclohexyl phthalate | C_20_H_26_O_4_ | ND | ND | 1061740 | ND | ND | ND | ND | ND |
| 23:01 | 279.1586 | Phthalic acid, 2-propylpentyl tetradecyl ester | C_30_H_50_O_4_ | 479736 | ND | ND | ND | ND | ND | ND | ND |
| 23:01 | 167.0340 | Phthalic acid, 4-methylhept-3-yl octadecyl ester | C_34_H_58_O_4_ | ND | ND | ND | ND | ND | 98277 | ND | ND |
| 23:0 | 342.0169 | Phthalic acid, heptadecyl 2-propylpentyl ester | C_33_H_56_O_4_ | ND | ND | 1223681 | 6918906 | ND | ND | ND | ND |
| 23:01 | 281.1662 | Bis(2-ethylhexyl) phthalate | C_24_H_38_O_4_ | ND | ND | ND | 7183382 | 1554457 | ND | 991269 | ND |
| 23:02 | 431.0869 | Tetradecamethylhexasiloxane | C_14_H_42_O_5_Si_6_ | 105223 | ND | ND | ND | ND | ND | ND | ND |
| 23:14 | 298.1203 | Pterocarpin | C_17_H_14_O_5_ | ND | ND | ND | 49277 | ND | ND | ND | ND |
| 23:17 | 132.0810 | 19-Methyl-eicosanoic acid, DMOX derivative | C_25_H_49_NO | ND | ND | ND | ND | 29139 | ND | ND | ND |
| 23:17 | 148.0670 | 4,8,12-Trimethyl-tridecanoic acid, pyrrolidide | C_20_H_39_NO | ND | ND | ND | ND | 35392 | ND | ND | ND |
| 23:17 | 150.0531 | Ethylphoshonic acid, fluoroanhydride, 2-methylbutyl ester | C_7_H_16_FO_2_P | ND | ND | ND | ND | NND | ND | 30705 | ND |
| 23:17 | 136.0515 | 23-Methyl-tetracosanoic acid, DMOX derivative | C_29_H_57_NO | ND | ND | ND | ND | ND | ND | 28356 | ND |
| 23:18 | 165.1278 | Methyl 2,3,4,6,7-penta-O-methyl-à-L-glycero-D-mannoheptopyranoside | C_13_H_26_O7 | ND | ND | 27763 | ND | ND | ND | ND | ND |
| 23:19 | 233.1147 | Hexacosane | C_26_H_54_ | 596158,5 | ND | 869606 | 906257 | 756681 | 541716 | 4607199 | ND |
| 23:20 | 196.2191 | Dodecane, 1-iodo- | C_12_H_25_I | ND | ND | ND | 1080114 | ND | ND | ND | ND |
| 23:26 | 270.0888 | Medicarpin | C_16_H_14_O_4_ | ND | ND | ND | 60108 | 381101 | ND | ND | ND |
| 23:46 | 132.0545 | 4-(3,4-Dihydroxy-2-oxo-butylamino)-benzonitrile | C_11_H_12_N_2_O_3_ | 63532 | ND | ND | ND | ND | ND | ND | ND |
| 23:47 | 222.0667 | 1-Cyclohexyldimethylsilyloxybutane | C_12_H_26_OSi | ND | ND | ND | ND | ND | ND | ND | ND |
| 23:54 | 337.2554 | 17-Octadecynoic acid | C_21_H4_0_O_2_Si | 101344 | ND | ND | ND | ND | ND | ND | ND |
| 23:57 | 294.0888 | 2-Propen-1-one, 1,3-bis[4-(dimethylamino)phenyl]- | C_19_H_22_N_2_O | ND | ND | ND | 84515 | ND | ND | ND | ND |
| 23:57 | 294.0887 | Ethene, 1-(anthracen-9-yl)-2-(p-tolyl)-, (E)- | C_23_H_18_ | ND | ND | ND | 85809 | ND | ND | ND | ND |
| 24:04 | 210.0299 | 3-(Cyclohexylamino)-2-trimethylsilyloxy-1-propanesulfonic acid, trimethylsilyl ester | C_15_H_35_NO_4_SSi_2_ | ND | ND | ND | ND | ND | ND | 13255 | ND |
| 24:05 | 341.2889 | Sulfurous acid, butyl hexadecyl ester | C_20_H_42_O_3_S | 927323 | ND | ND | ND | 671220 | ND | ND | ND |
| 24:05 | 264.2461 | 1-Eicosanol | C_20_H_42_O | ND | ND | 642027 | ND | 447759 | ND | ND | ND |
| 24:05 | 263.2372 | Isobutyl hexadecyl ether | C_20_H_42_O | ND | ND | ND | 155612 | ND | ND | ND | ND |
| 24:20 | 260.1172 | 2-Fluoro-5-nitrobenzyl alcohol, chlorodifluoroacetate | C_9_H_5_ClF_3_NO_4_ | 466797 | ND | ND | ND | ND | ND | ND | ND |
| 24:36 | 229.1078 | Xylitol, 1,5-anhydro-, triacetate | C_11_H_16_O_7_ | ND | ND | 92060 | ND | ND | ND | ND | ND |
| 24:37 | 313.2015 | 1,3-Benzenediol, O,O'-di(2-trifluoromethylbenzoyl)- | C_22_H_12_F6O_4_ | ND | ND | ND | 61838 | ND | ND | ND | ND |
| 24:37 | 271.1548 | 3-Trifluoromethylbenzoic acid, 3-chloroprop-2-enyl ester | C_11_H_8_ClF_3_O_2_ | ND | ND | 104071 | ND | ND | ND | ND | ND |
| 24:37 | 318.0656 | 4-Trifluoromethylbenzoic acid, 2-bromo-4-fluorophenyl ester | C_14_H_7_BrF_4_O_2_ | ND | ND | ND | 88783 | ND | ND | ND | ND |
| 24:38 | 239.2367 | 2-Oxo-2H-chromene-3-carbothioic acid S-phenyl ester | C_16_H_10_O_3_S | ND | ND | ND | ND | ND | ND | 32173 | ND |
| 24:38 | 229.1069 | Eicosanoicacid, 3-hydroxy-, ethyl ester | C_11_H_8_ClF_3_O_2_ | ND | ND | ND | ND | ND | 30385 | ND | ND |
| 24:38 | 229.1078 | 3-Indolethanamine, N-[(á-t-butyramino)propionyl]- | C_19_H_27_N_3_O_4_ | ND | ND | 29447 | ND | ND | ND | ND | ND |
| 24:44 | 276.2697 | 9-Octadecenamide | C_18_H_35_NO | ND | ND | 191703 | ND | ND | ND | ND | ND |
| 24:44 | 276.2708 | Oleic acid amide | C_18_H_35_NO | ND | ND | 173721 | ND | ND | ND | ND | ND |
| 24:46 | 298.0849 | benzene, 1,1'-(1,2-ethynediyl)bis[2,4-dimethoxy- | C_18_H_18_O_4_ | ND | ND | ND | 47408 | ND | ND | ND | ND |
| 24:54 | 191.0558 | Hexadecanamide | C_16_H_33_NO | 32478 | ND | ND | 75469 | ND | ND | ND | ND |
| 24:54 | 186.1210 | 3-[3-(4-Hydroxyphenyl)-3-oxoprop-1-en-1-yl]-6-methylchromen-4-one | C_19_H_14_O_4_ | ND | ND | ND | 43727 | ND | ND | ND | ND |
| 25:15 | 222.2307 | 3',4'-Methylenedioxy-a-pyrrolidinopropiophenone | C_14_H_17_NO_3_ | ND | ND | 15615 | ND | ND | ND | ND | ND |
| 25:19 | 420.3544 | à-Tocospiro A | C_29_H_50_O_4_ | ND | ND | ND | ND | ND | ND | 45113 | ND |
| 25:20 | 420.3561 | à-Tocospiro B | C_29_H_50_O_4_ | ND | ND | ND | ND | ND | ND | 61802 | ND |
| 25:30 | 336.1358 | Silane, dimethyl(4-acetylphenoxy)nonyloxy- | C_19_H_32_O_3_Si | ND | ND | ND | 196398 | ND | ND | ND | ND |
| 25:31 | 224.2503 | 1-Heptacosanol | C_27_H_56_O | ND | ND | ND | 1668140 | ND | ND | ND | ND |
| 25:31 | 210.2338 | Tetratriacontyl pentafluoropropionate | C_37_H_69_F_5_O_2_ | ND | ND | ND | 1563563 | ND | ND | ND | ND |
| 25:57 | 266.0208 | 4-Trifluoromethylbenzoic acid, 1-cyclopentylethyl ester | C_15_H_17_F_3_O_2_ | ND | ND | 78965 | ND | ND | ND | ND | ND |
| 26:02 | 393.3376 | Benzenamine, 4-octyl-N-(4-octylphenyl)- | C_28_H_43_N | 85305 | ND | 106606 | ND | ND | ND | ND | ND |
| 26:02 | 393.3371 | Benzenamine, 4-(1,1,3,3-tetramethylbutyl)-N-[4-(1,1,3,3-tetramethylbutyl)phenyl]- | C_28_H_43_N | ND | ND | 164436 | ND | ND | ND | ND | ND |
| 26:11 | 417.3718 | Tetratetracontane | C_44_H_90_ | 560262,5 | ND | ND | 609819 | ND | 385747 | 812662 | ND |
| 26:16 | 352.1307 | 2-Pyridyl[2-trifluoromethyl-4-benzo[h]quinolyl]ketone | C_20_H_11_F_3_N_2_O | ND | ND | ND | 242946 | ND | ND | ND | ND |
| 26:20 | 176.1519 | Benzeneethanamine, 2-fluoro-á,3,4-trihydroxy-N-isopropyl- | C_11_H_16_FNO_3_ | 54919 | ND | 42711 | 34700 | 62168 | ND | 126159 | ND |
| 26:45 | 212.1512 | Isophthalic acid, di(2-methylprop-2-en-1-yl) ester | C_16_H_18_O_4_ | ND | ND | ND | ND | ND | ND | 94494 | ND |
| 26:52 | 225.2575 | Hexatriacontane | C_36_H_74_ | ND | ND | ND | 1801398 | 402893 | ND | ND | ND |
| 26:52 | 309.3515 | 2-methyloctacosane | C_29_H_60_ | 5785968 | ND | ND | 2076746 | 504084 | 397742 | 2385061 | ND |
| 26:54 | 154.1714 | Dipipanone | C_24_H_31_NO | ND | ND | ND | 23689 | ND | ND | ND | ND |
| 26:59 | 379.3347 | Cholesta-4,6-dien-3-ol, (3á)- | C_27_H_44_O | ND | ND | ND | ND | 215944 | ND | 152671 | ND |
| 27:08 | 396.3734 | Stigmasta-3,5-diene | C_29_H_48_ | 201154 | ND | ND | ND | ND | ND | ND | ND |
| 27:15 | 430.3804 | dl-à-Tocopherol | C_29_H_50_O_2_ | 65650 | ND | ND | ND | ND | ND | ND | ND |
| 27:28 | 395.3304 | Olean-13(18)-ene | C_30_H_50_ | ND | ND | ND | 59985 | ND | ND | ND | ND |
| 27:31 | 434.3762 | Tetracosane, 11-decyl- | C_34_H_70_ | 1127758 | ND | ND | ND | ND | ND | ND | ND |
| 27:56 | 317.0325 | Sulfurous acid, butyl tridecyl ester | C_17_H_36_O_3_S | 745539 | ND | ND | ND | ND | ND | 1100058 | ND |
| 28:27 | 414.3822 | Lanost-8-en-3-ol, (3á)- | C_30_H_52_O | ND | ND | 75265 | ND | 1021475 | ND | ND | ND |
| 28:35 | 391.0512 | 1,2-Dioctanoin | C_19_H_36_O_5_ | ND | ND | 159304 | ND | ND | ND | ND | ND |
| 28:36 | 414.3867 | á-Sitosterol | C_29_H_50_O | 163779 | ND | 987809 | ND | ND | ND | ND | ND |
| 28:36 | 414.3862 | ç-Sitosterol | C_29_H_50_O | ND | ND | ND | ND | ND | ND | 45945 | ND |
| 28:39 | 411.3577 | Cycloartanyl acetate | C_32_H_54_O_2_ | ND | ND | ND | 234399 | ND | ND | ND | ND |
| 28:39 | 281.3202 | Triacontane | C_30_H_62_ | 663029,5 | ND | ND | 1230424 | 1000184 | ND | ND | ND |
| 28:40 | 411.3571 | Cholest-22-ene-21-ol, 3,5-dehydro-6-methoxy-, pivalate | C_33_H_54_O_3_ | ND | ND | ND | ND | ND | ND | 1199188 | ND |
| 28:49 | 414.3819 | 9,19-Cyclolanostan-3-ol, acetate, (3á)- | C_32_H_54_O_2_ | ND | ND | 424412 | 294356 | 553756 | ND | ND | ND |
| 29:03 | 424.3703 | Lup-20(29)-en-3-one | C_30_H_48_O | ND | ND | ND | 450095 | ND | ND | ND | ND |
| 29:12 | 426.3839 | Lupeol | C_30_H_50_O | ND | ND | ND | 256141 | ND | ND | ND | ND |
| 29:16 | 358.0682 | Cyclohex-2-enone, 3-[2-(5-methoxy-2-methyl-1H-indol-3-yl)ethylamino]-2-pentanoyl- | C_23_H_30_N_2_O_3_ | ND | ND | ND | 99416 | 118970 | ND | ND | ND |
| 29:24 | 267.0000 | 1,1,1,2,2,3,3-Heptafluoro-3-(1,2,2,2-tetrafluoroethoxy)propane | C_5_HF_11_O | ND | 50716 | ND | ND | ND | ND | ND | ND |
| 29:30 | 225.2581 | Docosane | C_22_H_46_ | ND | ND | ND | ND | ND | ND | 778301 | ND |
| 29:33 | 425.3784 | 9,19-Cycloergost-24(28)-en-3-ol, 4,14-dimethyl-, acetate, (3á,4à,5à)- | C_32_H_52_O_2_ | 258462 | ND | ND | ND | ND | ND | ND | ND |
| 29:36 | 388.9816 | 9à-3,22-Dihydroxy-dihydrosolasodine | C_27_H_45_NO_3_ | ND | ND | ND | 35998 | ND | ND | ND | ND |
| 30:32 | 647.4213 | benzenamine, 4,4'-[4-[1-[4-[4-(diethylamino)phenyl]-3-cyclohexen-1-yl]-1-methylethyl]cyclohexylidene]bis[N,N-diethyl- | C_45_H_65_N_3_ | ND | ND | ND | 74554 | ND | ND | ND | ND |

R.T. = Retention time, ND = Not detected, *m/z* = Mass spectrum.
